# Intrinsic and Network Mechanisms Constrain Neural Synchrony in the Moth Antennal Lobe

**DOI:** 10.3389/fphys.2016.00080

**Published:** 2016-03-08

**Authors:** Hong Lei, Yanxue Yu, Shuifang Zhu, Aaditya V. Rangan

**Affiliations:** ^1^Department of Neuroscience, The University of ArizonaTucson, AZ, USA; ^2^Institute of Plant Quarantine, Chinese Academy of Inspection and QuarantineBeijing, China; ^3^Department of Mathematics, Courant Institute of Mathematical Sciences, New York UniversityNew York, NY, USA

**Keywords:** antennal lobe, afterhyperpolarization (AHP), projection neuron, local neuron, disinhibition, computational model, synchrony, multiple-firing-event (MFE)

## Abstract

Projection-neurons (PNs) within the antennal lobe (AL) of the hawkmoth respond vigorously to odor stimulation, with each vigorous response followed by a ~1 s period of suppression—dubbed the “afterhyperpolarization-phase,” or AHP-phase. Prior evidence indicates that this AHP-phase is important for the processing of odors, but the mechanisms underlying this phase and its function remain unknown. We investigate this issue. Beginning with several physiological experiments, we find that pharmacological manipulation of the AL yields surprising results. Specifically, (a) the application of picrotoxin (PTX) lengthens the AHP-phase and reduces PN activity, whereas (b) the application of Bicuculline-methiodide (BIC) reduces the AHP-phase and increases PN activity. These results are curious, as both PTX and BIC are inhibitory-receptor antagonists. To resolve this conundrum, we speculate that perhaps (a) PTX reduces PN activity through a disinhibitory circuit involving a heterogeneous population of local-neurons, and (b) BIC acts to hamper certain intrinsic currents within the PNs that contribute to the AHP-phase. To probe these hypotheses further we build a computational model of the AL and benchmark our model against our experimental observations. We find that, for parameters which satisfy these benchmarks, our model exhibits a particular kind of synchronous activity: namely, “multiple-firing-events” (MFEs). These MFEs are causally-linked sequences of spikes which emerge stochastically, and turn out to have important dynamical consequences for all the experimentally observed phenomena we used as benchmarks. Taking a step back, we extract a few predictions from our computational model pertaining to the real AL: Some predictions deal with the MFEs we expect to see in the real AL, whereas other predictions involve the runaway synchronization that we expect when BIC-application hampers the AHP-phase. By examining the literature we see support for the former, and we perform some additional experiments to confirm the latter. The confirmation of these predictions validates, at least partially, our initial speculation above. We conclude that the AL is poised in a state of high-gain; ready to respond vigorously to even faint stimuli. After each response the AHP-phase functions to prevent runaway synchronization and to “reset” the AL for another odor-specific response.

## Introduction

It has long been understood that recurrent connectivity as well as intrinsic cellular properties both play a role in the dynamics of the insect Antennal Lobe (AL) (Hansson and Anton, 2000; Vosshall et al., 2000; Assisi et al., 2007; Galizia and Rössler, 2010). Nevertheless, it is still unclear how these features interact, and to what extent they influence the functional properties of the AL. In this paper we investigate this question within the hawkmoth (*Manduca sexta*) AL.

The *Manduca* AL itself houses many interneurons, including both Local Neurons (LNs) as well as Projection Neurons (PNs) which send information further downstream (Homberg et al., 1989; Lei et al., 2010). These neurons are organized into functional and morphological modules—a.k.a. glomeruli—which are each stimulated by different classes of odorants. In this paper we largely concentrate on two such glomeruli—named the “cumulus” and “toroid” in male moth—which form the so-called Macroglomerular Complex (MGC) (Matsumoto and Hildebrand, 1981; Christensen and Hildebrand, 1987). This MGC serves as the first central stage of detection and processing of conspecific female sex-pheromones, and plays a crucial role in many of the *Manduca*'s mating behaviors (Schneiderman et al., 1986; Hansson et al., 1991).

Our previous work, along with the work of others, has shown that PNs and LNs within the *Manduca*'s MGC respond—with a vigorous depolarization—to brief puffs of odor containing the appropriate chemical components found in the animal's sex-pheromones (Warren and Kloppenburg, 2014; Kim et al., 2015; Lavialle-Defaix et al., 2015). Intriguingly, the response of the PNs also drops precipitously after each brief odor pulse—a phenomenon we refer to as the “After HyperPolarization” (AHP) phase of each response (Lei et al., 2002; Reisenman et al., 2005). Our previous work has shown that this AHP-phase is somehow implicated in odor-processing: pharmacological manipulation which interferes with the AHP-phase also prohibits *Manduca* from reliably detecting and responding to pheromone pulses (see e.g., Lei et al., 2009). Moreover, similar AHP-like phases have been widely reported as important for the sensory systems of many other animals (Wilson and Goldberg, 2006; Saito et al., 2008). Thus, rather than being a mere curiosity, the AHP-phase seems to be a rather general dynamical feature which plays a necessary functional role in sensory processing.

Our goal in this paper is to probe the dynamical mechanisms responsible for the AHP-phase and its associated currents within the *Manduca* AL. As mentioned above, we expect these mechanisms to include both intrinsic cellular properties (see e.g., Pedarzani et al., 2005), as well as recurrent connectivity (see e.g., the role played by GABA-B receptors discussed in Otmakhova and Lisman, 2004; Wilson and Laurent, 2005). Some intrinsic and recurrent mechanisms have also been studied in the modeling work done by Belmabrouk et al. (2011a,b). By clarifying how these mechanisms either compete or assist one another, we hope to reveal some of the computational principles at work in the olfactory system.

The main conclusions of this paper are that the dynamics of the hawkmoth antennal-lobe are consistent with: (a) strong heterogeneous inter- and intra-glomerular synaptic connectivity, and (b) slow inhibitory intrinsic currents acting on the PNs. Feature (a) grants the AL a kind of automatic-gain-control—i.e., allowing the AL to respond very sensitively to faint odor puffs with the robust activation of multiple PNs—involving a kind of synchrony we refer to as “multiple-firing-events.” Feature (b) protects such a sensitive AL from “runaway synchronization,” allowing the AL to respond effectively to sequences of odor-stimuli separated by a few hundred milliseconds.

The Results section of our paper is organized as follows. First, in Section R1 we describe some of our experimental results. These experiments involve the application of various pharmacological agents to the AL, and motivate our computational model, which we discuss in Section R2. We use our computational model to try and understand the kinds of dynamics which underlie the phenomena we observe in experiment. This computational model then informs several predictions (Section R3), some of which we test in Section R4. Finally, we close with a discussion; touching on possible consequences of our investigation, as well as related work.

## Materials and methods

In this section we give an overview of our experimental and computational methods. This section is reinforced by material in the online Supplementary Information.

### Insect preparation

*Manduca sexta* (L.) (Lepidoptera: Sphingidae) were reared in the laboratory on artificial diet under a long-day photoperiod, and adult male moths, 4 days post-emergence, were prepared for experiments as described previously (Hansson et al., 1991). For electrophysiological recordings, the moth was restrained in a plastic tube with its head fully exposed. The labial palps, proboscis and cibarial musculature were then removed to allow access to the brain. To eliminate movement, the head was isolated and pinned to a wax-coated glass Petri dish with the ALs facing upward. Tracheae and a small part of the sheath overlying one AL were then removed with fine forceps. The preparation was continuously superfused with physiological saline solution containing 150 mM NaCl, 3 mM CaCl2, 3 mM KCl, 10 mM TES buffer (pH 6.9), and 25 mM sucrose.

### Electrophysiological recording

To allow long-term recording from single neurons, which is needed for the pharmacological experiments in this study, we used a juxtacellular recording technique modified from Pinault (1996) and tested in Lei et al. (2009). In short, electrodes resembling those used for patch recording were pulled from thin-wall borosilicate glass capillaries using Sutter P-2000 laser puller and filled with physiological saline, resulting in <10 mΩ electrode resistance. An Axoprobe-1A amplifier connected to a 10x DC amplifier (Model FC-23B, WPI, Sarasota, FL) was used to amplify the signal up to 1000x. Calibration pulses from the Axoprobe-1A amplifier were added to the output channels. A Leica micromanipulator was used to advance the electrode into the MGC region of an AL until a contact similar to that used for perforated-patch recording was achieved. A key technique in this configuration is to bring the electrode tip close to a neurite, nearly touching but not impaling it. We found that the amplitude of the recording was affected by the closeness of the electrode tip with the neurite. During the course of an entire experiment the relative position between the juxtacellular-electrode and neurite may drift, causing visible changes in the amplitude of the recorded spikes, but not their frequency or timing.

### Sensory stimulation and characterization of neurons

Olfactory stimuli were delivered to the preparation by injecting odor-laden air puffs onto a constant air flow that was controlled at 1 liter per minute. The flow was directed at the middle of the antenna ipsilateral to the AL from which recordings were made. Trains of 5 air puffs (50 ms) with 2 s inter-pulse intervals were generated by means of a solenoid-activated valve controlled by an electronic stimulator (WPI, Sarasota, FL). Shorter or longer intervals were used in particular experiments to test the effect of intervals on response consistency (**Figure 2**). These air puffs were directed through a glass syringe containing a piece of filter paper, bearing various amounts of a single pheromone component (0.1–100 ng in decadal steps). Not every concentration was used in all experiments. The stimulus compounds used were: (i) E10,Z12-hexadecadiennal (EZ, the primary component of the conspecific female's sex pheromone); (ii) E11,E12,Z14-hexadecatriennal (EEZ, a second essential component of the sex pheromone). MGC-PNs were characterized using 3 physiological criteria: (1) randomly bursting spontaneous firing pattern; (2) response specificity to pheromone components; and (3) multiphasic pattern of responses. In *M. sexta*, uniglomerular MGC-PNs have been shown repeatedly to give predictable responses to the pheromone components according to the MGC glomerulus in which their dendrites arborize (Christensen and Hildebrand, 1987; Heinbockel et al., 1999, 2004; Lei et al., 2002): Cumulus PNs are excited by antennal stimulation with EEZ but inhibited (or not affected) by stimulation with EZ, whereas the Toroid PNs are excited by stimulation with EZ but inhibited (or not affected) by stimulation with EEZ. These types of PNs typically exhibit a biphysic response pattern in juxtacellular recordings, i.e., a depolarization phase followed by a period of afterhyperpolarization (Lei et al., 2009). Finally, the spontaneous activity of MGC-PNs typically is more randomly bursting, while that of LNs is more tonic (Lei et al., 2011).

### Pharmacological manipulation

Picrotoxin, bicuculline methiodide, L-2-4-diaminobutyric acid and nipecotic acid (Sigma-Aldrich, >95%) were diluted in physiological saline solution to 200 μM and then bath-applied to moth preparations as described previously (Lei et al., 2009). In short, pharmacological agents were applied to moth preparations through a syringe drip system. The time when the drugs took effect was determined by observing the change of spontaneous activity of the recorded neuron. Spontaneous activity and odor-evoked responses were first recorded under the normal physiological saline solution and then repeated under the drug treatment, and finally the normal saline wash. Note that the final saline wash was typically applied many minutes after the initial recordings, during which the juxtacellular electrode may drift slightly, reducing the amplitude of the recorded spikes (see e.g., **Figure 4A**).

### Data acquisition and analysis

Spike traces were digitized at 25 kHz sampling rate using Datapack 2k2 software (Run Technologies, Mission Viejo, CA), and the time stamp of each spike was extracted off-line with the event-extraction function within the software package. The spike train data (columns of time stamps) were imported into a custom-written Matlab (The Mathworks Inc, Natick, MA) script, which calculates interspike-interval derived parameters such as mean instantaneous firing-rate and duration of the afterhyperpolarization. To determine the width of the response window, the spike train data were exported into Neuroexplorer (Nex Technologies, Littleton, MA) for plotting the peri-stimulus time histograms (PSTH), which allowed approximate estimation of response duration. Then the average of instantaneous spiking frequency (i.e., inverse of inter-spike interval) within the response window was calculated. We chose a 500 ms period starting from 120 ms after the onset of solenoid opening as response window. We also examined different window size such as 400, 600, and 750 ms and found no significant changes on our quantification of responses. This robustness may be due to the fact that the measurement is derived from averaging individual interspike intervals (ISI). In order to measure the duration of the afterhyperpolarization, we compared the ISI in a sequential manner after the stimulus onset. If an interval is at least 5 times longer than its previous interval, this later interval is considered as the afterhyperpolarization. All statistical comparisons were performed using the Statistics Toolbox of Matlab. To statistically compare the pharmacological effects in a balanced data set (i.e., across the same group of neurons at different stages, such as control vs. drug vs. wash), we selected the non-parametric Friedman's test (**Figures 4, 8**). Where there were only two groups in comparison (**Figures 6, 9**), we selected the non-parametric Mann-Whitney *U*-test. In both tests, the cut-off for type-I error were set at the 5% level (i.e., alpha = 5%). Following the Friedman's test, the Tukey-Kramer multi-comparison method was applied to determine the pairwise significance level.

### Computational model

We constructed a spiking network model of the AL with a modest number of architectural features—allowing it to simulate certain kinds of AL phenomena—while at the same time having few enough parameters to allow for serious benchmarking and subsequent investigation. While we sketch out our model in this section, the full details of our model are contained within the Supplementary Information.

**Figure 1:**
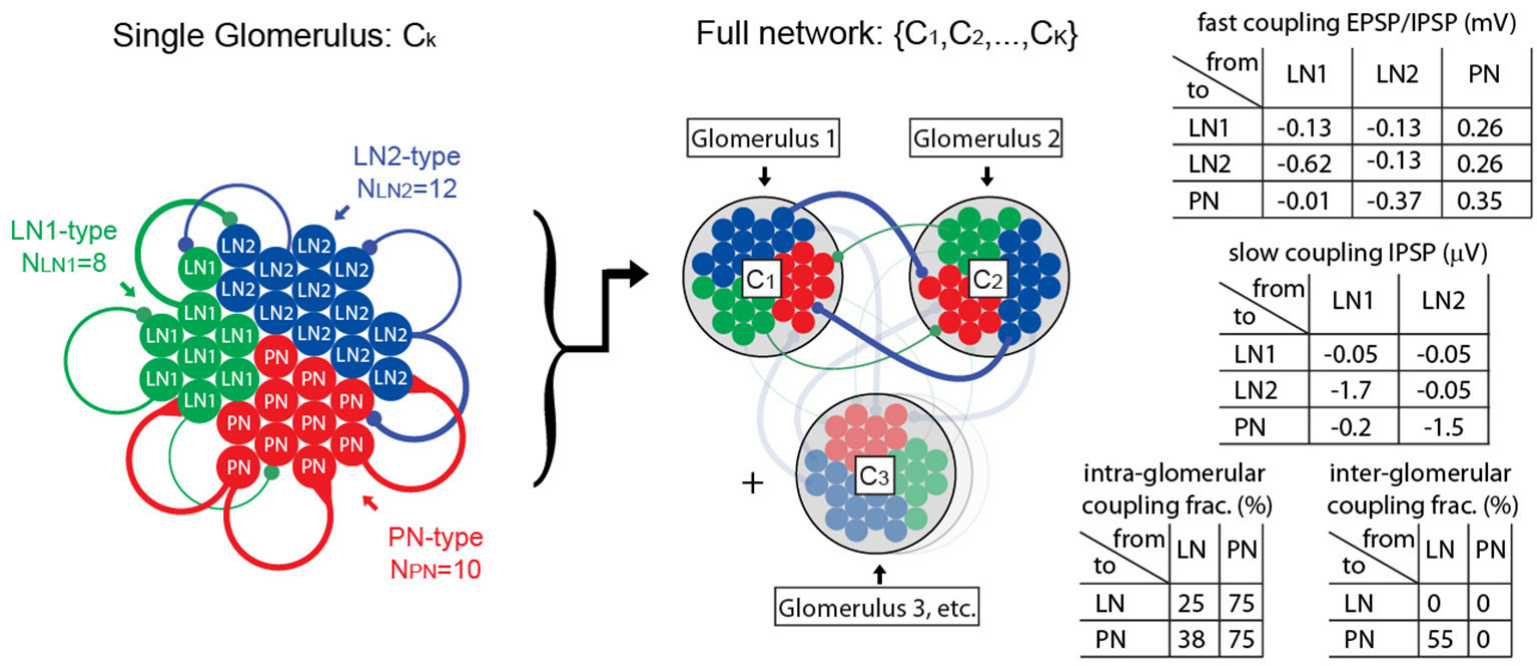
**Illustration of computational network architecture**. On the left we illustrate the anatomy of a single glomerulus, comprising 10 PNs, 8 LN1s, 12 LN2s, each modeled using single-compartment integrate-and-fire equations. These neuronal populations are connected together sparsely via local intra-glomerular connections. Representative connections are illustrated in this diagram with colored lines, the thickness of which roughly corresponds to the coupling strength. In the middle we illustrate our full network, comprising K glomeruli (e.g., K = 2–6). The neuronal populations within each of these glomeruli are connected together sparsely via long-range inter-glomerular connections; these long-range connections have the same synaptic strength as the intra-glomerular connections, but are randomly connected using different sparsity coefficients. On the right we list a few tables for the synaptic coupling strengths (given in terms of approximate EPSP and IPSP amplitudes), and sparsity coefficients. A full description of the network, as well as the parameters involved, is given in the Supplementary Information.

The network model discussed in this paper contains PNs, as well as two subclasses of local neurons: LN1s and LN2s. These neurons (totaling several dozen altogether) are organized into clusters that represent distinct glomeruli. The neurons are interconnected, both within each glomerulus and across glomeruli. This connectivity is illustrated in Figure 1.

Within our network model, each neuron is modeled by a single-compartment integrate-and-fire equation, driven by a combination of intrinsic, feedforward and synaptic currents:

$$\begin{array}{l} \tau_{V}\frac{d}{dt}V\left(t\right)=-\left(V-V^{L}\right)+I^{SK}+I_{fast}^{input}+I_{fast}^{syn,LN1}+\;I_{slow}^{syn,LN1}\\ +I_{fast}^{syn,LN2}+I_{slow}^{syn,LN2}+I_{fast}^{syn,PN}, \end{array}$$

The intrinsic currents determine how each neuron responds to stimuli, and are different for the different neuron types (e.g., *PN*s are equipped with *SK*-channels). The feedforward-input currents are independent (uncorrelated) between neurons, and are given by a feedforward Poisson input with time-varying rate. This feedforward Poisson input rate—which again depends on neuron type—comprises both a background (low rate) plus the time-varying stimulus-induced input (which can be high rate).

The synaptic currents involve recurrent nicotinic-type excitation (2 ms timescale), as well as GABA-A-type inhibition (2 ms timescale), as well as a slower synaptic inhibition (e.g., GABA-B-type with a ~750 ms timescale). The coupling strengths depend on the pre- and post-synaptic neuron types (e.g., the LN1 population inhibits the LN2s differently than the PNs). In our model we assume that local neurons (LN1s and LN2s) are inhibitory, whereas PNs are excitatory. We do not explicitly model any excitatory local neurons (see Olsen et al., 2007, as well as Shang et al., 2007), although the effective inter- and intra-glomerular excitation associated with such neurons might be similar to the excitatory effects of our PNs (see Huang et al., 2010).

The recurrent connectivity matrix for our network is chosen to be an Erdos-Renyi random graph (i.e., each edge chosen independently with some given coupling probability) with coupling probabilities that are functions of the pre- and post-synaptic neuron type and are slightly different for inter-glomerular connections vs. intra-glomerular connections.

As we will discuss below, we use our model to conduct numerical simulations: we subject our model to various stimuli while attempting to mimic a variety of experimental conditions. One important detail within this methodology is how we translate PTX and BIC application from the real world to our model. For our purposes, we will simulate PTX application as though PTX reduces the efficacy of GABA-A type receptors. When our model is operating under the influence of PTX (i.e., “PTX-on” condition), the postsynaptic currents associated with GABA-A synapses will be reduced by 75%. We similarly reduce by 75% the postsynaptic GABA-A currents under BIC application. In addition, we drastically reduce the SK-currents under this “BIC-on” condition (as motivated by the discussion in Section R1). Going forward, we will compare and contrast the behavior of our model in the PTX-on and BIC-on conditions with the “control” or CTRL-condition (i.e., CTRL = fully functional GABA-A and SK currents).

We emphasize two important features of our network are:

1. We ensure that the inhibitory synaptic connections made by our LNs are “heterogeneous”; i.e., the distribution of post-synaptic connection strengths varies widely across the LN population. As mentioned above, we enforce this heterogeneity by dividing our LNs into two “subclasses” labeled LN1 and LN2. While both subclasses of LNs are connected sparsely and randomly to the other neurons in our model, the distribution of connection strengths is different for the LN1 and LN2 subclasses. This heterogeneity implies that some LNs will mostly inhibit PNs, without inhibiting too many other LNs, whereas some other LNs will do the opposite. This heterogeneity is crucial for allowing our model to facilitate PTX-on disinhibition of the PNs (see Section R1 for discussion).
2. We ensure that the PNs are equipped with intrinsic SK-currents. These inhibitory currents are driven by each PN's own firing, serving to prevent that PN from firing multiple times in a row. Once elevated, this current persists for quite some time, decaying after ~400 ms. The presence of such a persistent intrinsic inhibitory current is crucial for allowing our model to facilitate the BIC-on shortening of the AHP-phase (see Section R1 for discussion).

### Benchmarking the model

The model described above has several parameters which influence its dynamics. These parameters include both the strength and sparsity of the synaptic connections, as well as the strength of the intrinsic SK-currents and feedforward currents. Many of these parameters are constrained somewhat by physiology (e.g., the synaptic coupling strengths must be compatible with the observed sizes of EPSPs and IPSPs). Nevertheless, even as these parameters are varied within physiological bounds, the network can still produce a wide variety of dynamical regimes, ranging from the physiologically realistic to the unrealistic.

In order to further constrain these network parameters we “benchmark” our model. That is, we choose a variety of experimentally observed phenomena associated with the real AL (i.e., benchmarks) and demand that our network satisfy these benchmarks. Given any particular set of parameters—thought of as a point in parameter space—our network will operate within a particular dynamical regime and, generally speaking, few-to-none of these benchmarks will be satisfied. Our goal is to find a region in parameter space that corresponds to dynamical regimes that satisfy *all* of our benchmarks; we hope that these dynamical regimes will be “realistic” to some extent.

**Figure 2:**
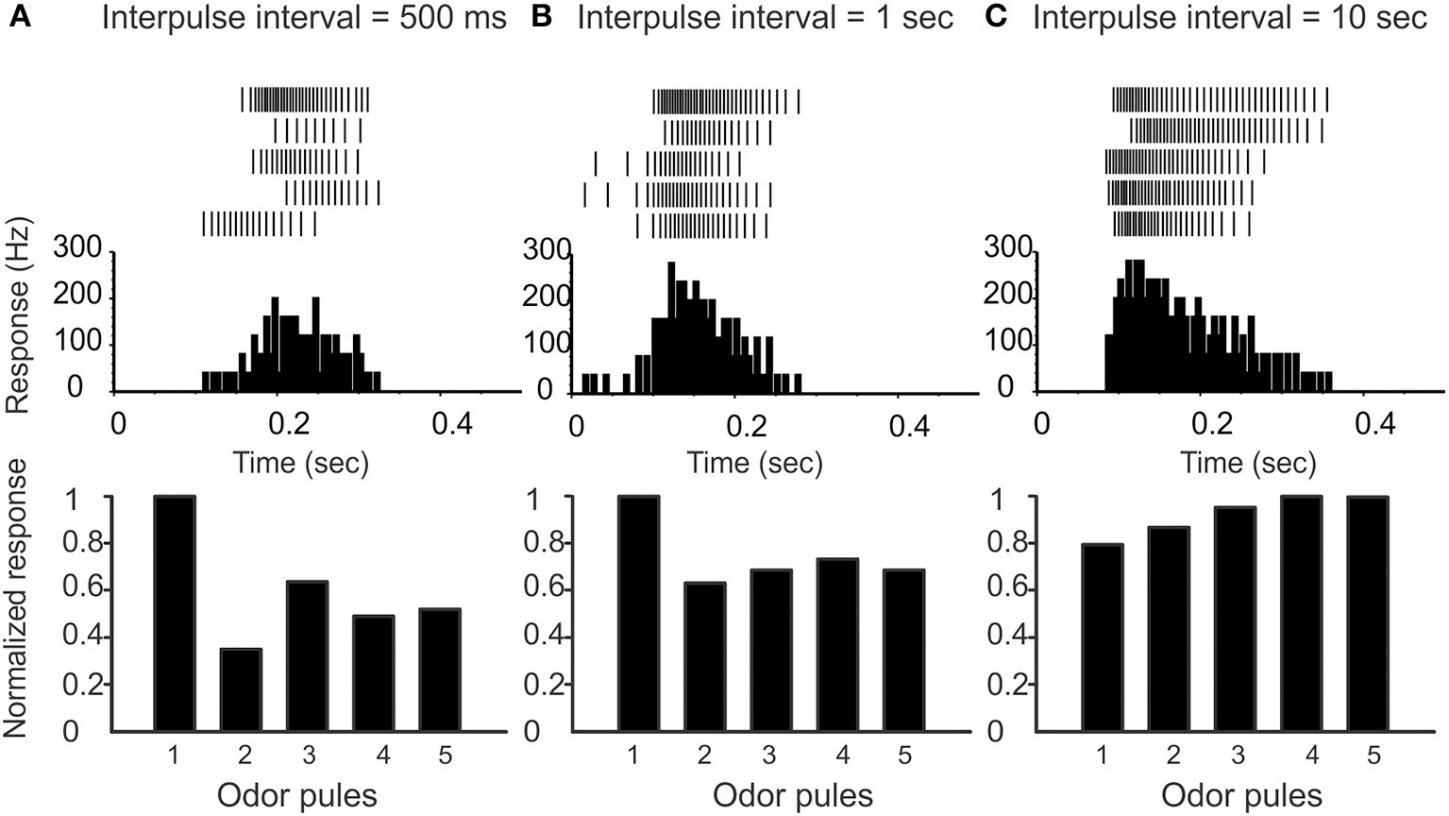
**Varying the inter-pulse-interval (IPI) affects the attenuation of PN responses**. **(A)** On top we show raster-plots of a PN response to five pulses in sequence (IPI 500 ms). The peristimulus-time-histogram is shown in the middle, and the normalized response per pulse is shown on the bottom. Note that there is a marked attenuation of PN response following the first pulse. **(B)** IPI = 1 s, and the attenuation is less marked. **(C)** IPI = 10 s, which is significantly greater than the AHP-phase; there is no attenuation of PN response.

**Figure 3:**
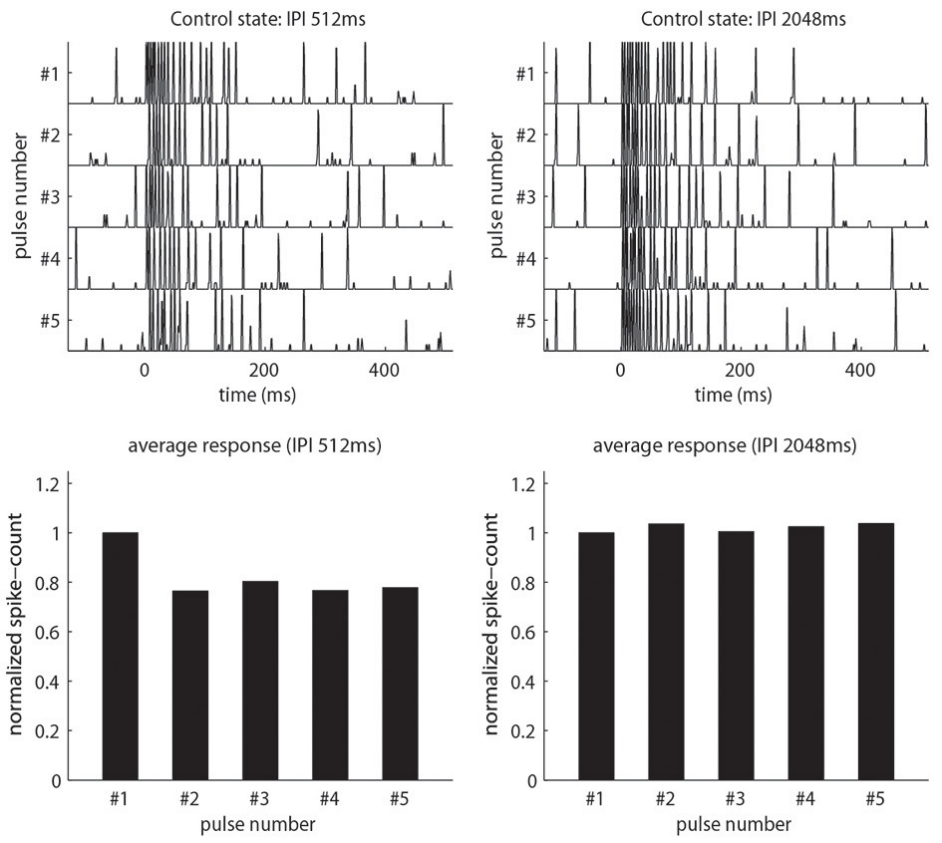
**Our model qualitatively reproduces the pulse-response attenuation seen in Figure 2**. On top we show traces of PN-activity for a model glomerulus responding to a sequence of five pulses. On the bottom we show the normalized spike-count per-pulse. On the left we use an IPI of 512 ms. On the right we use an IPI of 2048 ms, which is significantly longer than the AHP-phase.

**Figure 4:**
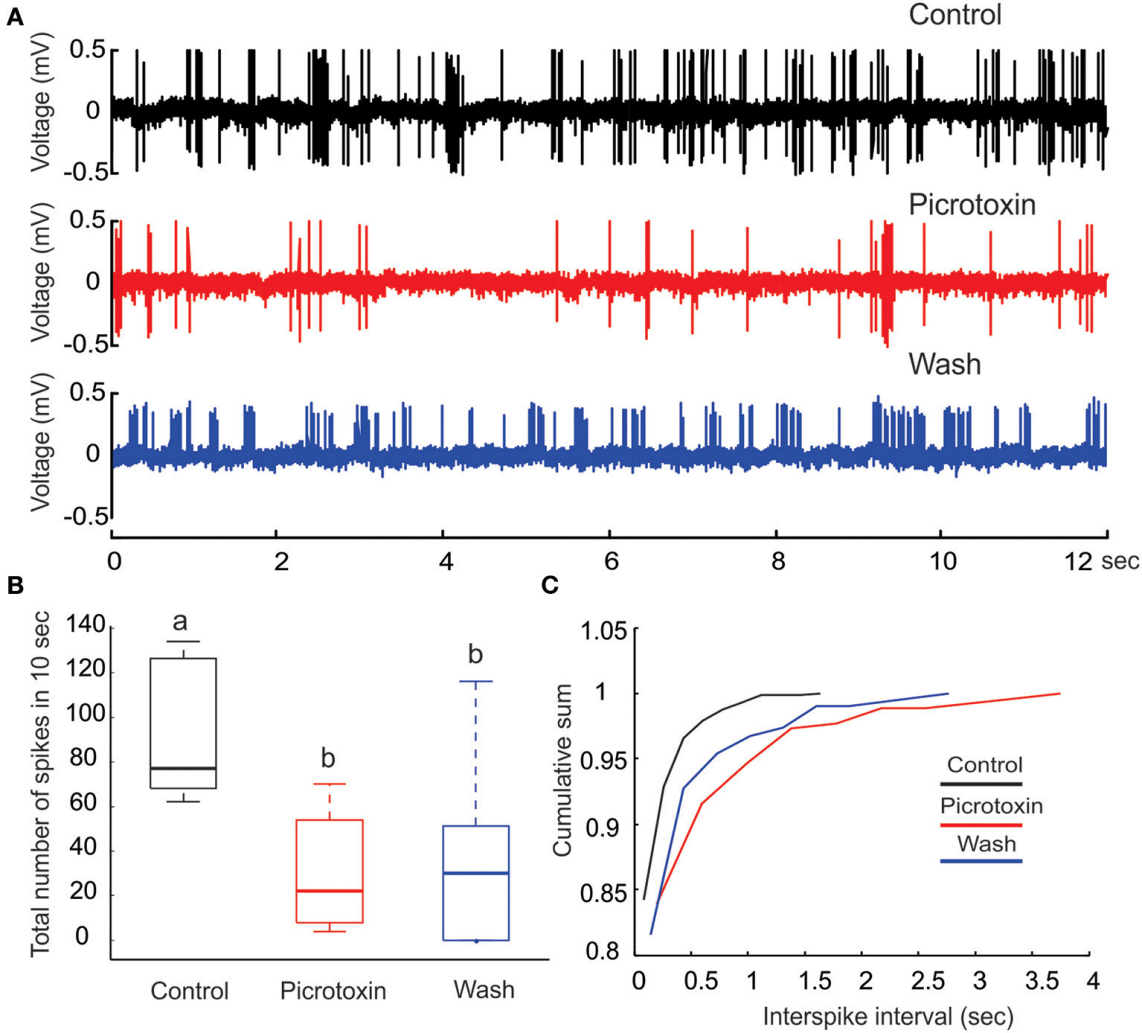
**Picrotoxin reduces PN's spontaneous activities**. **(A)** Juxtacellular recording traces (12 s long) show a marked reduction in the number spikes due to the application of picrotoxin. **(B)** The reduction of number of spikes due to picrotoxin is statistically significant (*Friedman* test, *p* < 0.01, *n* = 8) but the effect is not fully reversible. Different letters on top of the box plots indicate statistical significance. **(C)** Cumulative sum of interspike intervals (ISI) shows that the picrotoxin treatment prolongs the maximal ISI to about 4 s (red line) while the maximal ISI under saline control is only about 1.5 s (black line). Moreover, nearly all (about 98%) ISIs under saline control are less than 0.5 s but about 87% of ISIs under picrotoxin are within this range. Saline wash produces a pattern that is between the drug treatment and control (blue line).

**Figure 5:**
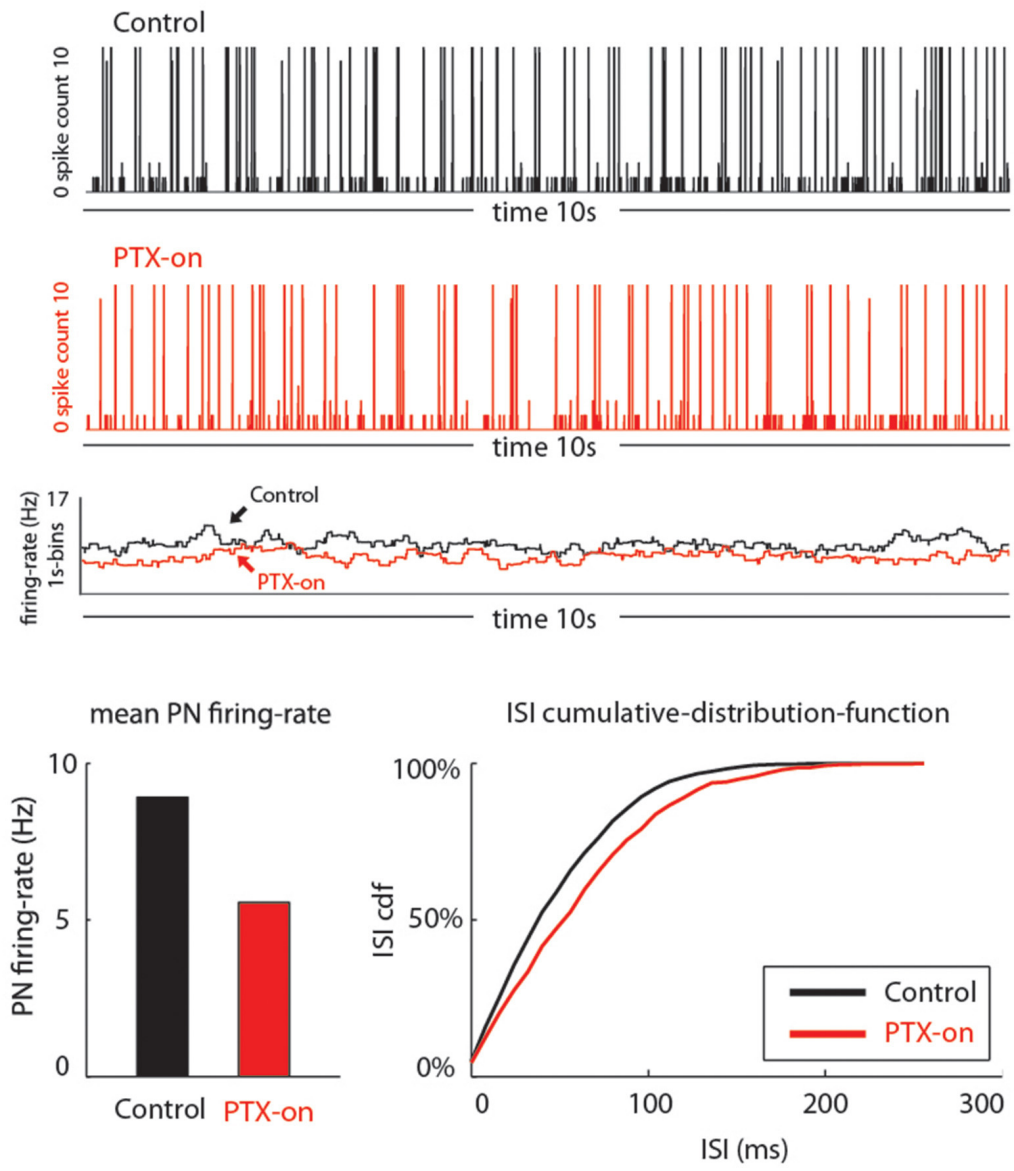
**Our model qualitatively reproduces the PTX-induced phenomena seen in Figure 4**. On top we show traces of PN-activity for a model glomerulus in background, both in the control state (black) and PTX-on state (red). Below these traces we plot the time-averaged PN firing-rate (averaged over 1 s bins). On the bottom we show the average PN firing rates (left) and cumulative-distribution-function for the ISI-intervals (right).

Our benchmarks are listed below:

1. Firing-rates, EPSPs and IPSPs: In the real AL, PN and LN firing-rates are between 5 and 15 Hz in background (i.e., when unstimulated), and usually between 40 and 80 Hz when stimulated. EPSPs and IPSPs are usually smaller than 1 mV. We require that corresponding values for our network lie within these acceptable ranges.
2. Pulse-response attenuation: In the real AL, the PNs will respond less vigorously to an odor puff if that puff was immediately preceded by a previous puff. This phenomenon gives rise to attenuation of the PN response to a rapid sequence of odor pulses. We require that our network exhibit a similar attenuation when stimulated with simulated odor pulses. Compare Figure 2 and Figure 3.
3. PTX response when unstimulated: This benchmark is intended to capture the phenomena discussed in Section R1. As mentioned above, the pharmacological application of PTX to the real AL is translated in our network to the reduction of GABA-A presynaptic currents by ~75%. We require that, when compared against CTRL, the PTX-on condition exhibit both (i) a reduction in spontaneous PN firing-rates, as well as (ii) an increase in the typical spontaneous PN ISIs. Compare Figure 4 and Figure 5.
4. PTX response when stimulated: This benchmark is intended to capture the effects of PTX on the PN response to a train of odor pulses. As per experiment, we require that (i) the mean PN response per pulse for the PTX-on condition should be comparable to that of the CTRL-condition, whereas (ii) the standard-deviation in PN response per pulse should be significantly higher when PTX is on. Compare **Figure 10** and **Figure 11**.
5. BIC response when stimulated: This benchmark is intended to capture the phenomena discussed in Section R1. As mentioned above, the pharmacological application of BIC to the real AL is translated in our network to the combination of (i) a reduction of GABA-A presynaptic currents by ~75%, as well as (ii) a reduction in the strength of the intrinsic SK-currents that follow hyperpolarization of the PNs. We require that, when BIC is on, our model PNs exhibit prolonged responses to odor stimuli; responses that show a much diminished AHP-phase. Consequently, the BIC-on state should reduce PN pulse-response attenuation and prevent PNs from faithfully tracking rapid sequences of odor pulses. Compare (Lei et al., 2009) with Supplementary Figure S7.
6. BIC response when unstimulated: This benchmark is intended to capture the effects of BIC on the spontaneous state. We require that, when BIC is on, the PNs exhibit slow modulation in their background dynamics, switching between long epochs of periodic firing and long epochs of relative silence (typical epoch length should be several seconds to tens of seconds). Compare **Figure 12** and **Figure 13**.

To actually perform our benchmarking we repeatedly scanned sections of parameter-space by varying one or two parameters at a time, covarying the most influential parameters whenever possible. For each scan we chose the “best” set of parameters (i.e., those which came closest to satisfying our benchmarks) and scanned again; varying different parameters the next time. This repeated parameter-scanning was done by hand (and not automated) so that (i) we could gain some intuition for the vastly different kinds of dynamic-regimes our network was capable of producing, and (ii) we could be sure that our results were not too sensitive to any single parameter. We continued searching until we found a large open region in parameter-space, each point of which gave rise to a rather similar dynamical regime that exhibits all of our benchmarks. Figure 1 lists sample values for many of these coupling and connectivity parameters for one point within such a region.

After benchmarking our network, we investigated the mechanisms at work within the resulting dynamical regime. We found that the dynamical regime that supported the above phenomena was one of “high-gain,” with strong recurrent connectivity that gives rise to multiple-firing-events (MFEs). This regime is discussed at length below in Section R2.

## Results

### Section R1: initial experiments

In this section we present some of our experiments which hint at the nature of the after-hyperpolarization (AHP) phase in projection neuron (PN) response. These experiments will strongly suggest that the AHP-phase comprises both inhibitory synaptic currents as well as hyperpolarizing intrinsic currents.

**Figure 6:**
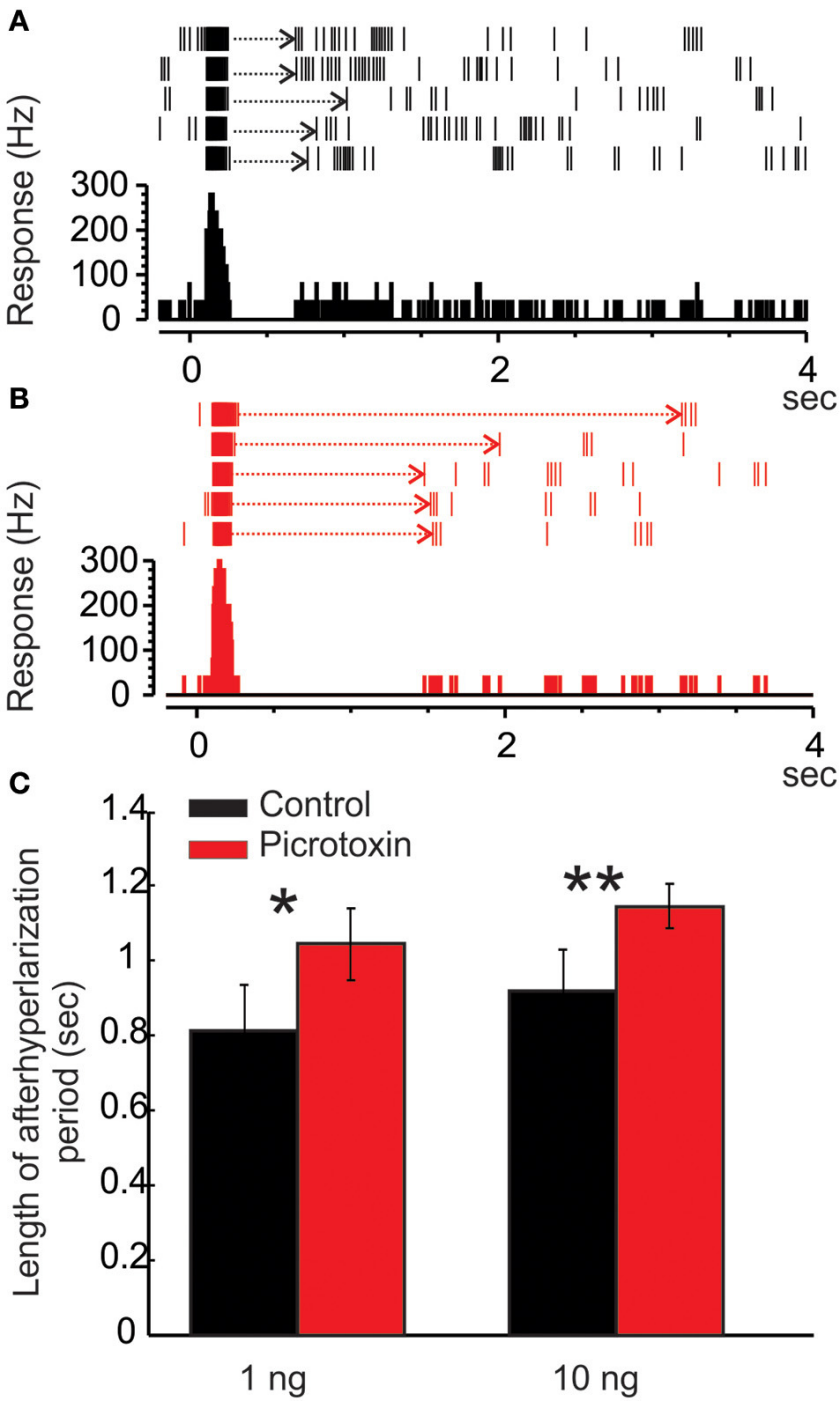
**Picrotoxin increases the duration of afterhyperpolarization (AHP)**. **(A)** Raster plot and peristimulus histogram show a typical excitatory response of a Toroid PN to five pulses of EZ stimulation. The stimulus onset is at time zero and lasts for 50 ms. Note the AHP (dotted arrows) following the bursts of excitatory responses. **(B)** Under the picrotoxin treatment, the duration of AHP is significantly increased, along with a reduced spontaneous activity. **(C)** Group data showing that the picrotoxin-induced increase of AHP duration is statistically significant either under 1 or 10 ng stimulation (*Mann Whitney U*-test, *p* < 0.05 or 0.01, *n* = 8; asterisks indicate the significance level).

To preface, recall that the “control-condition” (i.e., saline wash, rather than any active pharmacological agent) produces spontaneous PN activity in the range of 6–12 Hz (see, e.g., Figure 4A). When stimulated by a pheromone pulse, the PN activity increases vigorously, and is usually followed by an AHP-phase, expressed as a nearly silent period in the raster plots and PSTH following each pulse (see e.g., Figure 6A). This AHP-phase not only truncates the excitatory response evoked by each odor pulse but also lasts for about a second or so. As a result, the AHP-phase caused by any given odor pulse can interfere with—and reduce—the magnitude of excitatory response to any subsequent odor pulse occurring shortly after the first. To quantify this attenuation, we stimulate the MGC with a rapid sequence of five successive odor pulses (see methods) characterized by an “inter-pulse-interval” (IPI) ranging from IPI = 0.5–10 s. As expected, the PN response shows a marked attenuation when the IPI is less than or equal to the observed duration of the AHP (see Figure 2). On the other hand, when the IPI = 2 s or longer, the AHP from each pulse dies away before the next pulse arrives, and so the AHP does not significantly affect the PN response across pulses (i.e., there is little to no attenuation when IPI ≥ 2 s).

Our first set of experiments perturbs the scenario above through the pharmacological application of picrotoxin (PTX) to the MGC. PTX has been shown to be an effective GABA-A receptor antagonist in both vertebrate and invertebrate preparations (Newland and Cull-Candy, 1992; Anthony et al., 1993; Laurent et al., 1999; Lee et al., 2003; Choudhary et al., 2012; Warren and Kloppenburg, 2014). Consequently, we expect PTX application to increase the PN response. However, to the contrary:

#### PTX decreases PN's spontaneous activity

Under our experimental conditions, perfusing the moth AL with PTX (200 μM) significantly reduced the level of spontaneous activities on PNs (Figures 4A,B; *Friedman* test, *p* < 0.01, *n* = 8). Despite a reduction of the number of spikes (from 70 to 120 with median of 79 in a 10-s window to 10–50 with median of 20, Figure 4B), the bursting pattern was not altered (Figure 4A, middle panel). Apparently, the reduction of number of spikes was primarily caused by the increase of ISI, especially those intervals between the bursts. This inference was further confirmed by plotting the cumulative probability sum of ISI across saline control (812 ISIs pooled from eight neurons), PTX treatment (261 ISIs) and saline wash (309 ISIs; Figure 4C). Without PTX (i.e., saline control), the maximal ISI was 1.72 s (Figure 4C, black line), but this number went up to 3.95 s with PTX, an increase of 129% (Figure 4C, red line). Moreover, the distribution of ISIs associated with the saline control group was shifted (toward shorter ISI times) relative to the distribution of ISIs associated with the PTX treatment. For example, 95% of the control-ISIs were shorter than 0.2 s, whereas this range only comprised about 85% of the ISIs under PTX-treatment. Saline wash did not reverse the ISI distribution to the control pattern completely, but rather to a pattern between the saline control and drug treatment (Figure 4C, blue line).

In addition to measuring the effects of PTX on spontaneous activity, we also measured the effects of PTX on the AHP-phase. We observed that:

#### PTX increases the duration of PN's AHP phase

**Figure 7:**
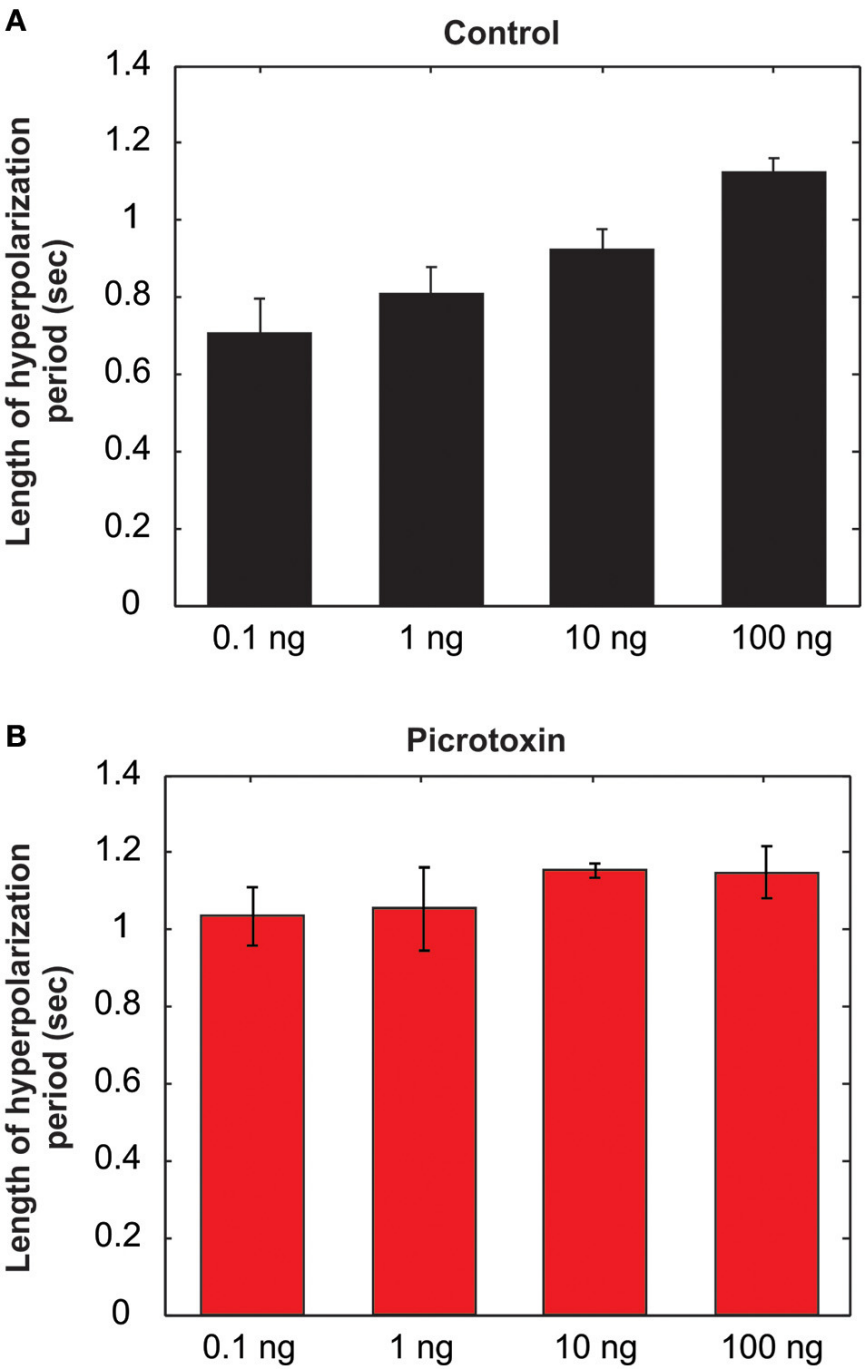
**The length of the afterhyperpolarization (AHP) period is positively related to the odor concentrations under saline control (A), but the linear relationship is lost after picrotoxin treatment (B) (***N*** = 8)**.

As mentioned above, the MGC PNs' excitatory response to pheromones is usually followed by an AHP-phase, expressed as a gap in the raster plots and PSTH (Figure 6A, dotted arrows). The length of the AHP period was significantly increased by PTX application (Figure 6B, dotted arrows). Because the AHP is positively correlated with odor concentrations (Figure 7A), we also compared the PTX effect on low (1 ng) and high (10 ng) dose evoked responses. In both cases, PTX significantly increased the length of AHP (*Mann Whitney U*-test, *p* < 0.05 or 0.01, *n* = 8; Figure 6C). Interestingly, PTX application disrupted the linear correlation between odor concentrations and the duration of AHP (Figure 7B).

Thus, despite the fact that PTX is a GABA-A receptor antagonist, perfusion of PTX actually enhances the inhibitory modulation of the PNs. Moreover, because PTX increases the duration of the AHP-phase, these effects likely stem from an increase in the inhibitory currents responsible for the AHP-phase, and not to secondary-effects of PTX which might block nicotinic-excitation (as seen, e.g., in honeybee, see Barbara et al., 2005). Based on these considerations, we will explore the hypothesis that the PNs may be involved in a disinhibitory network operating within the MGC or even spanning the AL (for motivation, see Christensen et al., 1998a or Buckley and Nowotny, 2011).

**Figure 8:**
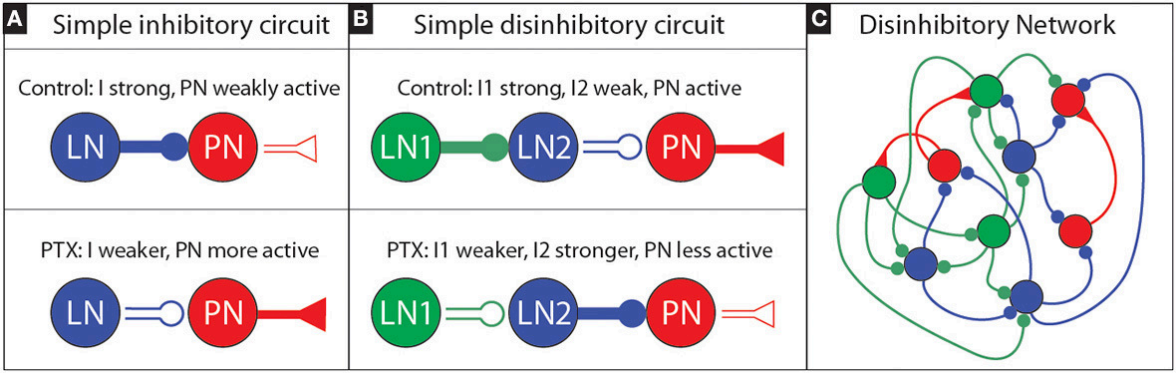
**Sketch of an inhibitory and disinhibitory circuit, as well as a more realistic disinhibitory network: (A)** We illustrate a simple inhibitory circuit, involving a single LN which produces presynaptic inhibitory current I that inhibits a single PN. Under PTX we expect the efficacy of inhibitory synapses to decrease; thus I should get smaller, implying that the PN activity will increase. **(B)** We illustrate a simple disinhibitory circuit, involving an LN1 which produces a presynaptic inhibitory current I1 that inhibits another LN2. This LN2, in turn, produces a presynaptic inhibitory current I2 that inhibits a single PN. If the activity level of the first LN1 is high, we may expect LN2 to be only weakly active, and for the PN to be active. Under PTX we expect that the efficacy of inhibitory synapses will decrease: as a result I1 will decrease, and so the activity level of LN2 will increase. This increased activity level—combined with the now decreased efficacy of inhibitory synapses—will alter I2. If the activity level increase of LN2 is sufficiently large, it is possible for I2 to actually increase overall (even though PTX has been applied). As a consequence, it is possible for the PN activity level to actually decrease overall—even though PTX has been applied. **(C)** A more realistic disinhibitory network would involve not just three neurons, but rather a collection of LNs and PNs, with the former heterogeneously coupled. In the heterogeneous network shown we have colored the various LNs according to the role they might play with regards to disinhibition: those LNs that predominantly inhibit PNs are classified as “LN2s” and colored blue, while the LNs that predominantly inhibit the LN2s are classified as “LN1s” and colored green. In reality the roles are not so clear cut; some LNs will both inhibit PNs as well as inhibit other LNs, and it may not always be possible to clearly classify each and every LN into a specific role.

As a very simple example of such disinhibition, one may consider a 3-neuron circuit consisting of a single local neuron (LN1) inhibiting a second local neuron (LN2) which inhibits a projection neuron (PN). The layout for this simple circuit is illustrated in Figure 8. We'll also assume—for exposition—that this simple circuit is operating in a mean-driven regime (see, e.g., Destexhe and Sejnowski, 2009; Buckley and Nowotny, 2011). In such a regime, each neuron receives independent feedforward input currents that—alone—would be sufficient to cause them to fire at high rates. As we'll discuss next, this mean-driven regime can be understood by analyzing its firing-rates.

The “control” situation for this simple circuit involves LN1 being very active with high firing-rate *m*_1_. In this condition, since LN1 is very active, the inhibitory presynaptic currents to LN2—denoted by “*I*_1_” will be proportional to the LN1 activity. That is to say, *I*_1_ will be roughly *S*×*m*_1_ for some “synaptic strength” *S*. The large presynaptic current *I*_1_ will ensure that LN2 is only weakly active, with a firing-rate *m*_2_ which will be a function [i.e., f(·)] of the total input current to LN2. In this case we expect *m*_2_ = *f*(*E*−*I*_1_) = *f*(*E*−*Sm*_1_), where f depends on both *I*_1_, as well as some background excitatory current *E*; *m*_2_ should be lower as *I*_1_ increases. The presynaptic inhibition to the PN—denoted by *I*_2_—will be proportional to *f*(*E*−*I*_1_), roughly determined by something like *I*_2_ = *S*×f(*E*−*I*_1_) = *Sf*(*E*−*Sm*_1_). Because *m*_1_ and *S* are high, *I*_1_ will be high, so *m*_2_ = *f*(*E*−*I*_1_) will be low, so *I*_2_ will be low, and the PN activity will be high.

When PTX is applied to this simple circuit, the situation will change. The LN1 will remain active, but no longer inhibit LN2 as much. If the application of PTX blocks, say, three-quarters of the GABA-A receptors, we might imagine the synaptic-strength *S* reduced to *S*. With this reduction the presynaptic inhibition to LN2 is only *I*_1_ = *Sm*_1_, and so the new (higher) firing rate of LN2 will be *m*_2_ = *f*(*E*−*Sm*_1_). This new firing-rate m_2_ might be much higher than before (i.e., the firing-rate may be a nonlinear function of the presynaptic currents), implying that the presynaptic inhibitory current to the PN will change to *I*_2_ = *Sm*_2_ = *Sf*(*E*−*Sm*_1_). If *f* has the appropriate structure, it is certainly possible that *I*_2_ might actually be higher under PTX than under the control condition. In such a situation, we would observe the PN activity drop under PTX.

To be clear, we are not suggesting that each PN in the MGC is the target of an idealized disinhibitory circuit such as in Figure 8B, nor that the MGC operates in a mean-driven regime where such a firing-rate analysis is valid. Rather, we are suggesting that perhaps the collection of LNs in the MGC may be interconnected in such a way as to give rise to a similar disinhibitory phenomena– even without any single simple mean-driven disinhibitory circuit existing in isolation (see e.g., Figure 8C). Put another way: we suggest that an appropriately *heterogeneous* LN population (i.e., a population of LNs that have varying degrees of connectivity and coupling strength, both to each other and to the PNs) might—as a gestalt—give rise to the PTX-induced phenomena we observed above.

If, indeed, the MGC PNs are targets of such emergent disinhibition, we would expect many of the results we see under PTX to also manifest under other pharmacological agents which increase the overall level of inhibition in the MGC. One way to test this intuition is to use GABA transporter blockers—specifically L-2-4-diaminobutyric acid (L-DABA) and nipecotic acid. These blockers should increase the GABA concentration in the tissue (Mbungu et al., 1995; Oland et al., 2010). As confirmed below, this increase in GABA concentration has similar effects to PTX:

#### GABA transporter blockers enhance AHP

**Figure 9:**
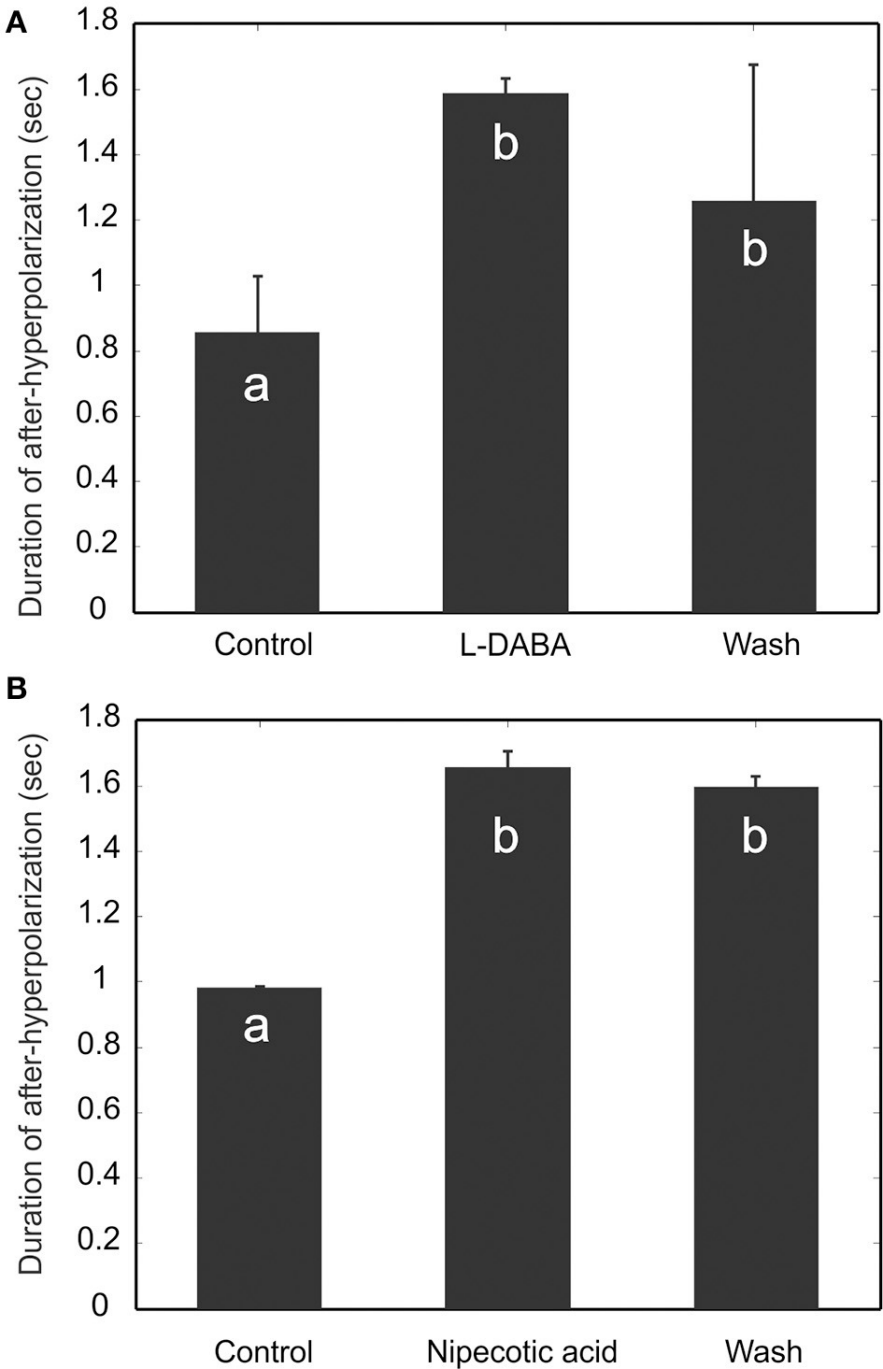
**Blocking GABA transporters increases the duration of afterhyperpolarization (AHP)**. Two GABA transporter blockers, L-DABA **(A)** and Nipecotic acid **(B)**, were used to treat the experimental preparations, and both blockers increase significantly the duration of AHP. The saline wash, however, does not fully reverse the drug effect. Different letters on the bar graphs indicate statistical significance (*Friedman* test, *p* < 0.01, *n* = 5).

We perfused the AL with GABA transporter blockers, L-2-4-diaminobutyric acid (L-DABA) and nipecotic acid. As expected, both blockers increased the AHP duration significantly (Figure 9) (*Friedman* test, *p* < 0.01, *n* = 5). The saline wash, however, did not have significant effects. This could be due to insufficient amount of washing time limited by recording sessions.

Based on the PTX, L-DABA and nipecotic acid experiments above, we concluded that (i) the PNs in the MGC participate in some kind of disinhibitory circuit, and (ii) that disinhibitory circuit gives rise to an inhibitory presynaptic current within the PNs that contributes to the AHP-phase.

While sensible given the experiments we've discussed so far, this conclusion is not obviously consistent with some of our previous experiments involving bicuculline methiodide (BIC; see Lei et al., 2009). To elaborate, BIC is similar to PTX, in that both agents are putative GABA-A receptor antagonists within the *Manduca* AL (Christensen et al., 1998b). Because there may be differences in the affinity of each agent for GABA-A, we don't expect BIC to act in exactly the same way as PTX. Nevertheless, at first blush we expected the effects of BIC to be qualitatively similar to PTX: i.e., to also lengthen the AHP within PN MGCs. To the contrary, however:

#### BIC eliminates the PN's AHP-phase

BIC application substantially reduces the length of the AHP-phase well below ~200 ms, and sometimes eliminates the AHP-phase altogether. Consequently, under BIC the PN response exhibits a much prolonged excitatory phase, persisting several hundred milliseconds after the pheromone stimulus is removed. In addition, due to the lack of an AHP-phase, the PN response exhibits little to no attenuation from one odor pulse to the next—even when those odor pulses are within 1 s of one another (e.g., an IPI of 512 ms). Thus, this BIC-induced lack of attenuation prevents PNs from faithfully tracking the dynamics of a pulsatile odor stimulus (Lei et al., 2009).

These results are surprising; the BIC induced phenomena within the MGC seem diametrically opposite to the PTX induced phenomena. Thus, even though they are both GABA-A receptor antagonists (Waldrop et al., 1987)^1^, PTX and BIC cannot be doing the same thing to the MGC.

One potential explanation for this paradox is that BIC is actually more than just a GABA-A receptor antagonist. Specifically, BIC could also block certain channels within the PNs – channels that give rise to intrinsic currents which, in the absence of BIC, ordinarily contribute to the AHP-phase (for precedent see Villalobos et al., 2004; Pedarzani et al., 2005; Belmabrouk et al., 2011a). While this leap of logic may seem farfetched at first, we believe that there is a natural candidate for such channels: namely, calcium-dependent small-conductance potassium channels (SK-channels). Indeed, in a functional study of cloned SK-channels using *Xenopus* oocytes, BIC was found to block two types of SK-channels (Khawaled et al., 1999).

Although there is no direct molecular evidence that proves that *Manduca* PNs possess SK-channels, there are several pieces of evidence that point toward this possibility:

1. In the fruit fly, *Drosophila melanogaster*, one type of SK channel was reported. Moreover, this channel is likely important for sensory processing, since the photoreceptors of the mutant flies lacking the gene of this channel produce oscillatory currents that hinder their responses under dim conditions (Abou Tayoun et al., 2011).
2. In mammals, SK-channels are believed to mediate the AHP-currents following action potentials, both inhibiting cell firing and limiting the firing frequency of repeatable action potentials (Bond et al., 1999; Adelman et al., 2012).
3. An SK channel homolog, KCNL-2, has also been characterized in the nervous system of *C. elegans* and is believed to regulate egg laying behavior (Chotoo et al., 2013).

Is it possible that *Manduca* PNs are indeed equipped with SK-channels, and that these channels are both partially responsible for the AHP and blocked by BIC? On the surface, this scenario might be consistent with the experiments described above. Recall that PTX and BIC gave rise to, respectively, a lengthening and shortening of the AHP-phase. Perhaps, as previously discussed, PTX reduces the effectiveness of GABA-A receptors, thus lengthening the AHP-phase of the PNs through a disinhibitory network of LNs. Now BIC should also reduce the effectiveness of GABA-A receptors somewhat, but could also block putative SK-channels within the PNs. While the former alone would reduce the PN activity, just like PTX, the latter could remove a substantial component of the AHP-currents, increasing PN activity. Perhaps a combination of these two effects could somehow result in both the PTX-induced phenomena we see above, as well as the BIC-induced phenomena observed in Lei et al. (2009).

Going forward, we will explore this possibility: We will use computational modeling to investigate the scenario sketched out in the previous paragraph. More specifically, we will create a spiking neuronal network that has (a) strong heterogeneous recurrent connectivity across the LN population, and (b) SK-channels within the PNs. We will determine whether or not it is even possible to benchmark such a network against the PTX- and BIC-induced phenomena described above. In doing so, we'll expose mechanisms that may be at work within the MGC or, more generally, across many glomeruli within the AL.

Before we embark on such a project, we comment on two somewhat more subtle phenomena we have observed; the first relating to PTX application, the second to BIC:

#### PTX disrupts PN's response consistency across repeated isolated stimuli

**Figure 10:**
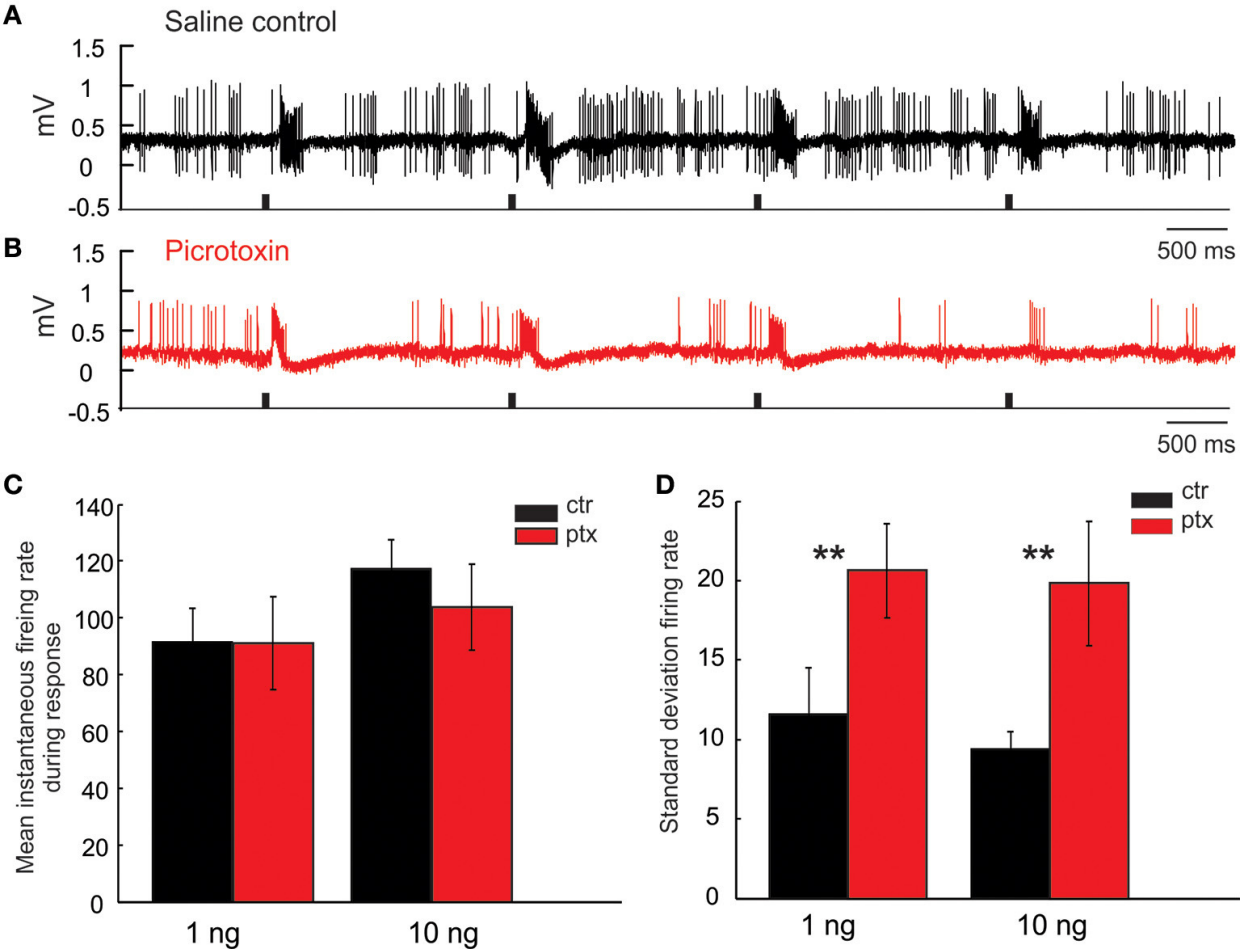
**Picrotoxin increases response variations**. **(A,B)** Juxtacellular recording traces show the responses of a Toroid PN to four pulses of EZ stimulation under saline control (black trace) and picrotoxin treatment (red trace). **(C,D)** Picrotoxin does not significantly change the mean instantaneous firing rate during the response window either to 1 or 10 ng stimulation; however, the treatment significantly increases the response variation measured by the standard deviation of firing rate across odor pulses (*Mann Whitney U*-test, *p* < 0.01, *n* = 8; asterisks indicate the significance level).

**Figure 11:**
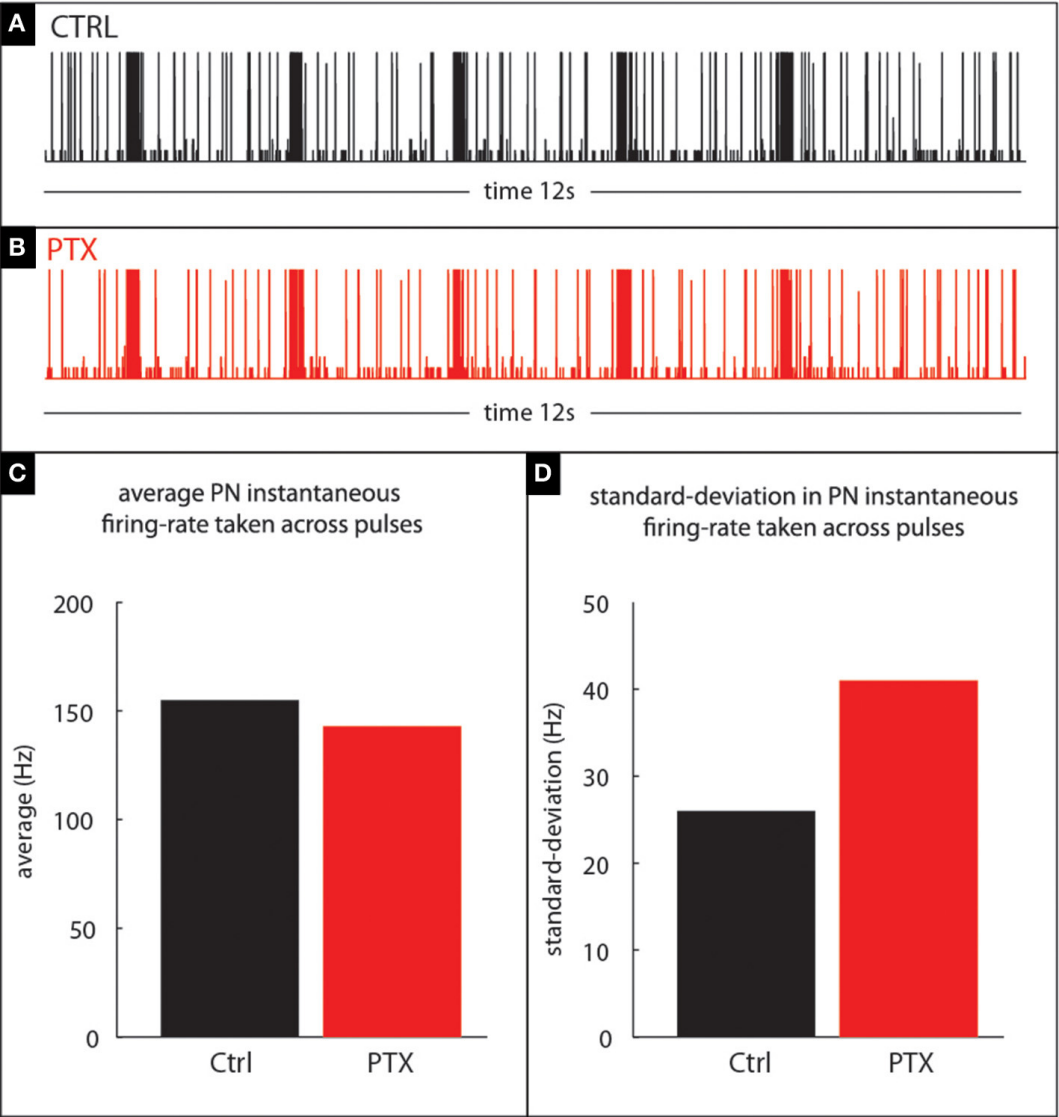
**Our model qualitatively reproduces the PTX-induced phenomena illustrated in Figure 10. (A,B)** PN-activity for a model glomerulus subject to a train of stimulus pulses separated by an IPI of 2 s. **(C)** The PTX-on state induces small changes in the mean PN-response—i.e., instantaneous firing-rate—averaged across pulses. **(D)** The PTX-on state induces larger changes in the standard-deviation (across pulses) of PN-response.

Recall that, in response to isolated pulses of pheromonal stimuli, the MGC PNs typically generate bursts of action potentials tracking each stimulus pulse (Figures 10A,B). Because the inter-pulse-interval (IPI = 2 s) in this case was sufficiently greater than the typical AHP-length (compare, e.g., Figure 6C with Figures 10A,B), the response from one pulse did not “interact” with the following pulse; consequently, there was little to no attenuation of the PN response across pulses. The same holds under PTX application, which did not significantly change the PNs' response magnitude, measured as the mean instantaneous firing rate during the response window across odor pulses (Figure 10C, *Mann Whitney U*-test, *p* > 0.05, *n* = 8). However, under PTX, the excitatory responses from pulse to pulse were not as consistent as those under the saline control, shown by a significant increase of the standard deviation of the mean instantaneous firing rate across all 5 odor-evoked responses (Figure 10D, *Mann Whitney U*-test, *p* < 0.01, *n* = 8). These drug effects were similarly observed when using low (1 ng) or high (10 ng) concentration of odors (Figures 10C,D). See Figure 11 for comparison with our model.

#### BIC introduces structure into the PN spontaneous activity

**Figure 12:**
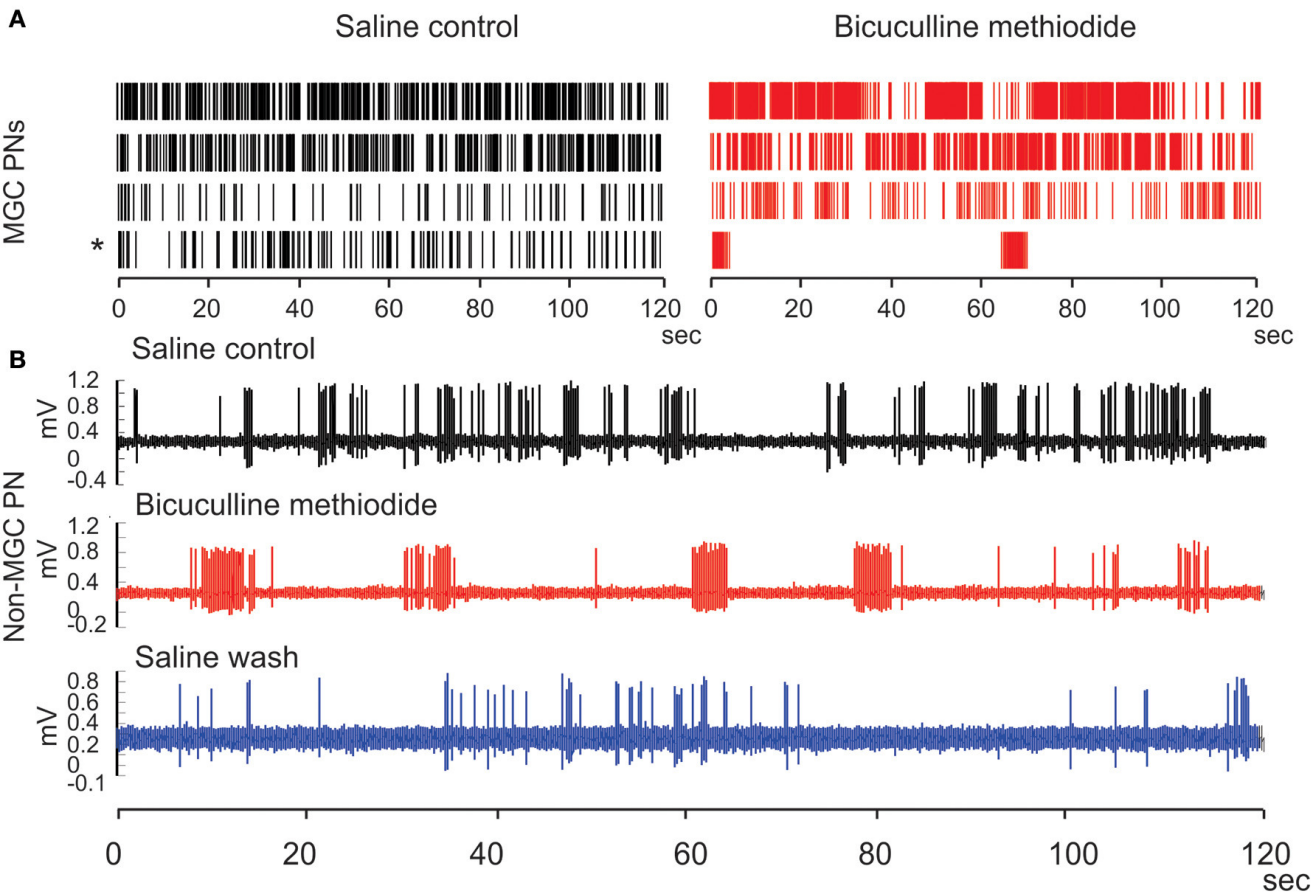
**Bicuculline methiodide (BIC) alters the spiking patterns of the spontaneous activity of PNs both in the MGC and in the ordinary glomeruli**. **(A)** Raster plots show that BIC treatment generally increases the spontaneous activity level of MGC PNs, opposite to the picrotoxin treatment, and produces the run-away pattern (asterisk) in some PNs. **(B)** Such run-away patterns are also present outside the MGC when BIC is used, verifying the predictions from the network model.

When unstimulated, the MGC PNs usually produce sporadic spontaneous activity with no obvious structure. Under BIC application, the spontaneous PN activity can change into a long-lasting structured pattern, which alternates between epochs of fast-periodic-spiking and epochs of near total quiescence. The epochs of fast-spiking are characterized by ISI-intervals of ~50 ms, whereas the quiescent epochs have firing-rates near 0 Hz. The epochs can each last for several tens of seconds, and alternation between the spiking and silent epochs continues for as long as BIC is supplied. The transition between any given spiking epoch and the subsequent silent epoch can be very abrupt—often much less than 100 ms—and sometimes even instantaneous. While we had originally observed this phenomenon in our previous work (Lei et al., 2009), we confirmed it once again with a new set of experiments. In these recent experiments we again observed BIC-induced spontaneous activity patterns, this time with even more variation than what we had originally seen in 2009. Although the spiking activity was generally increased by BIC application, only one MGC PN exhibited extreme rhythmicity when alternating between quiescent and spiking epochs (asterisk in Figure 12A). The other MGC PNs also exhibited long-lasting epochs of spiking as well as quiescent epochs, but were less rhythmic (Rows 1–3 of raster plots in Figure 12A).

These two phenomena both expose rather specific dynamic features of the MGC and neither is an obvious epiphenomenon of the mechanisms we have proposed so far. To elaborate, even if PTX does disrupt a disinhibitory network involving the PNs, why would such a disruption necessarily reduce the consistency of PN response across isolated pulses? Furthermore, even if BIC does block SK-channels—which have dynamics in the 100–500 ms range—why would blocking these channels give rise to structured spontaneous activity on a 10 s time-scale?

Thus, to further constrain and validate our computational modeling, we will use the above two phenomena as additional benchmarks. That is to say, we will determine if our computational model, possessing both (a) heterogeneous connectivity across the LNs and (b) SK-channels within the PNs, can reproduce all the phenomena discussed so far.

### Section R2: computational modeling

In this section we briefly describe our computational model, and use it to probe the potential consequences of disinhibition and SK-channels within the moth MGC.

Note that ours is certainly not the first model to investigate these mechanisms within the *Manduca* AL. For example, a mean-field model by Buckley and Nowotny (2011) analyzes the role of disinhibition within an idealized inhibitory network without fast synapses. As another example, a spiking network-model by Belmabrouk et al. (2011a) includes SK-type channels in order to replicate some of the pharmacological results seen in Lei et al. (2009)—specifically the elimination of the AHP-phase and diminished pulse-tracking properties observed under BIC-application.

We view both these works as encouraging, and take them as additional support for the disinhibition and SK-channel hypotheses. That being said, our model—which combines disinhibition and SK-channels—is the only model we are aware of that attempts to capture the broad range of PTX- and BIC-induced phenomena we observed in Section R1. Moreover, as we will eventually discuss later, our modeling study illuminates the importance of multiple-firing-events, which depend critically on fast synapses and cannot be well understood via a mean-field framework.

Our model is a spiking network model of a few interconnected glomeruli within the *Manduca* AL. This network is built out of several dozen spiking single-compartment integrate-and-fire neurons, using the voltages and conductances of the individual neurons as microscopic variables. Each glomerulus in our model corresponds to a relatively tightly knit cluster of a few dozen neurons, including inhibitory LNs and excitatory PNs. In terms of connectivity, we have abstracted the complex topology of the real AL as follows: we assume that different populations of neurons are interconnected sparsely and randomly, both within each glomerulus as well as across glomeruli. We remark that we do not explicitly model excitatory LNs, instead assuming that their effects are similar to the effects of the excitatory PNs (to which they may be strongly connected—see e.g., Huang et al., 2010).

With regards to the network's dynamics, we equip our neurons with fast synaptic currents, corresponding to nicotinic-type excitation and GABA-A-type inhibition, as well as slower inhibitory synaptic currents with a decay time ~750 ms. Both our LNs and PNs exhibit fast sodium-like spikes (modeled via the integrate-and-fire equations), but our PNs are also equipped with a slow intrinsic inhibitory current mimicking the putative SK-currents discussed above (decay time-scale ~400 ms). Each neuron in our network is also driven by independent feedforward Poisson input comprising (i) a background drive and (ii) a stimulus-specific drive targeting specific glomeruli at specific times.

These are the main ingredients of our model. Note that, as described in the Methods Section and in the Supplementary Material, our model has (a) heterogeneous recurrent inhibition provided by the LNs, as well as (b) slow intrinsic SK-currents within the PNs; while the latter is considered in Belmabrouk et al. (2011b), the former is not. The parameters for our model include excitatory and inhibitory couplings strengths (both within and across glomeruli), the strength of the SK-currents within the PNs, and the strengths of the feedforward input currents. As mentioned in the methods section and discussed in more detail in the Supplementary Material, we proceeded to tune this model by varying the parameters. Our goal when tuning was to search for parameters for which our model was “biologically plausible.” That is, for which our model satisfied all the benchmarks associated with our observations of the AL. We found that, indeed:

#### Our model allows for “biologically plausible” behavior

**Figure 13:**
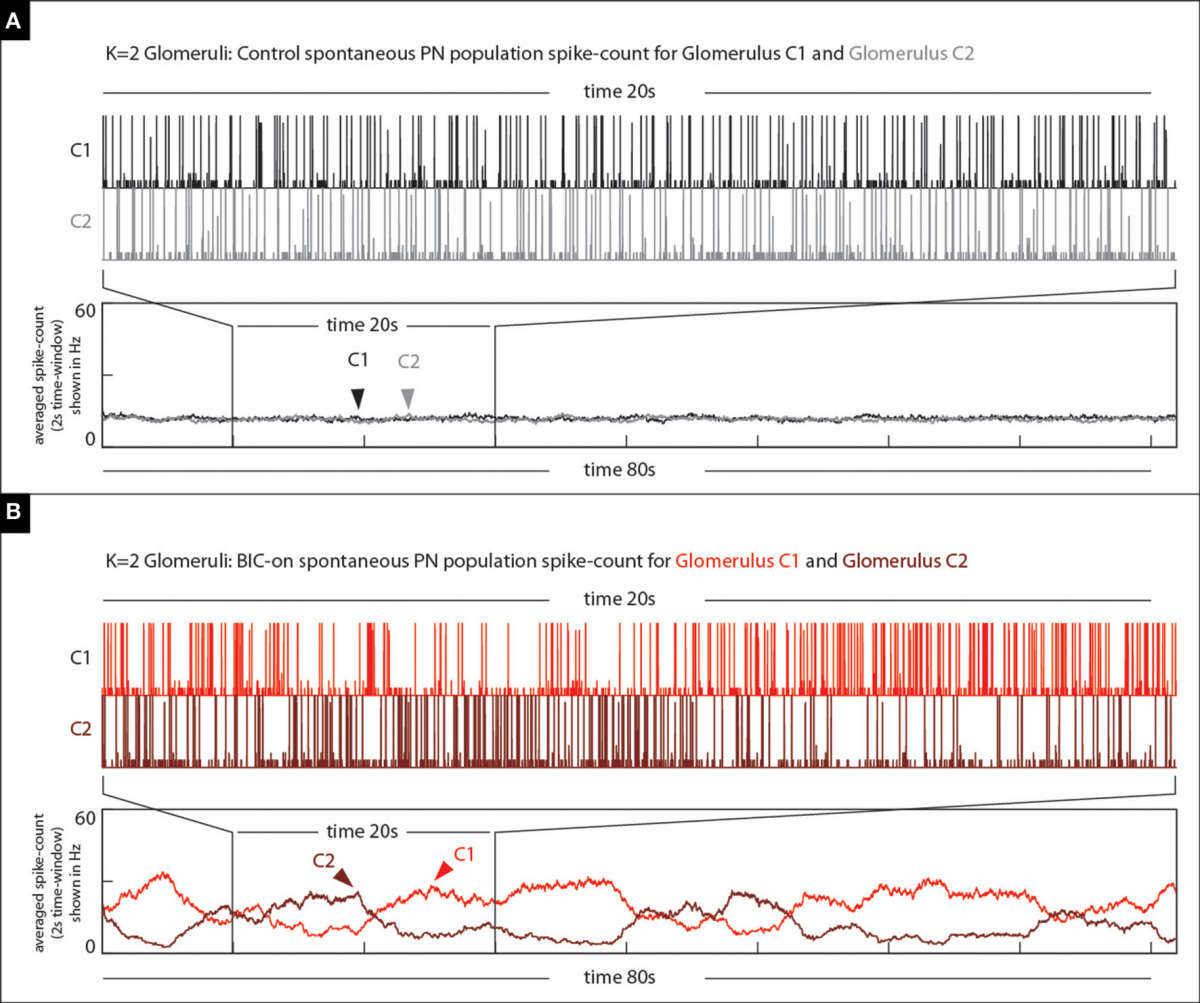
**Our model qualitatively reproduces the BIC-induced phenomena illustrated in Figure 12**. **(A)** On top we show the spontaneous PN-activity for both glomeruli (two shades of gray) in a *K* = 2 glomeruli model. The time-averaged spike-count (2 s bins) is shown on the bottom. **(B)** On top we show the spontaneous PN-activity for both glomeruli (two shades of red) in the same *K* = 2 glomeruli model under the BIC-on state. Note that not only are there long periods of elevated activity observed within each of the glomeruli, but also that these periods of activity are anti-correlated with one another.

There exists a region in parameter space for which our model can simultaneously exhibit the following phenomena discussed in Section R1: (1) Firing-rates, EPSPs and IPSPs similar to those observed in the real AL; (2) Pulse-response attenuation for IPIs < 1 s, (3) PTX-induced reduction in PN spontaneous firing rates, (4) PTX-induced loss of consistency across isolated stimulus pulses, (5) BIC-induced reduction in PN pulse-response attenuation and pulse-tracking, and (6) BIC-induced slow patterns when unstimulated. These phenomena are illustrated in Figures 3, 5, 11, 13, as well as in Supplementary Figure S7.

Even this modicum of success points toward the plausibility of our previous hypothesis. Namely, that the phenomena we observed might be due to (a) heterogeneous recurrent connectivity involving the LNs and (b) intrinsic SK-currents within the PNs. More importantly, however, our computational model gives a hint as to how these architectural mechanisms give rise to the phenomena at hand, and how those phenomena might coexist within a single dynamical regime.

After analyzing our model, we discovered that all the biologically plausible regimes we found shared a few things in common:

1. *Strong recurrent inhibition:* Unsurprisingly, all our biologically plausible regimes had large inhibitory coupling strengths. This is to be expected, as we engineered our model to possess a heterogeneous collection of LNs capable of disinhibition. In order for our network to exhibit the desired PTX-induced reduction in PN firing rates, the effects of such disinhibition should be significant. Strong recurrent inhibition seemed to be a necessary prerequisite for this.
2. *Strong intrinsic SK-currents:* Also unsurprisingly, all our biologically plausible regimes had large amplitude SK-currents within the PNs, corroborating previous modeling work by Belmabrouk et al. (2011a). This again is expected, as we required PNs in our model network to exhibit a BIC-induced reduction in the AHP-phase. Recall that, in our network, BIC-on corresponds to a reduction in both GABA-A-currents and SK-currents. Given that the former alone would lengthen the AHP-phase (through disinhibition), it is crucial that the AHP-phase also comprise a strong intrinsic inhibitory component—in our case this was an SK-current. In order for the BIC-on state to shorten the AHP-phase, this SK-current must be strong enough that its removal (under BIC) “outweighs” the additional presynaptic inhibition received by the PNs due to the disinhibitory network.
3. *High gain:* Our model functioned well when the feedforward, intrinsic and synaptic currents combined to ensure that, most of the time, at least some neurons in the network had membrane potentials that were not too far from the firing-threshold. This requirement is tantamount to the statement that—barring obvious exceptions such as PNs in the AHP-phase—the currents driving each neuron were neither overwhelmingly excitatory nor overwhelmingly inhibitory. This “high gain” regime allowed neurons to be responsive to fluctuations in their input currents; this responsivity played an important role in all the phenomena we sought.
4. *Strong recurrent excitation:* Finally, all our biologically plausible regimes had large excitatory coupling strengths. In this case “large coupling strengths” specifically means that the typical EPSP was of the same order as—or not too much smaller than—the typical IPSP coming from the heterogeneous network of LNs. This requirement can be thought of as a special case of the “high-gain” requirement from the previous paragraph, restricted to presynaptic currents. These large coupling strengths were instrumental in producing the somewhat more subtle dynamic phenomena discussed toward the end of Section R1.

These last two requirements—a high-gain state with strong recurrent excitation—were crucial for our model, and gave rise to a very important dynamic feature:

#### Our model exhibits emergent “multiple-firing-events” —or MFEs

These MFEs are a special kind of causally-linked synchronization across subsets of PNs. This brief synchronization occurs because the PNs are in a high-gain state; there are often a few PNs which are not too far from the firing-threshold. Because of the strong recurrent excitation, one or two typical EPSPs can close this gap, causing one spike to lead to the next. That is to say, it will not be uncommon for any given PN spike to drive at least one other postsynaptic PN over the spiking-threshold, causing a second PN spike almost immediately (i.e., within 1–2 ms). This second spike may cause a third, and so forth, resulting in a chain reaction including several PNs over a comparatively short period of time (say, <5 ms).

That these chain-reactions *can* occur depends on the high-gain and strong recurrent excitation; whether or not a chain-reaction *will* occur at any given time is due primarily to luck—which PNs have high subthreshold voltages and which do not. While it is certainly possible for MFEs to occur spontaneously (i.e., when the model is unstimulated), most MFEs occur during the initial response to stimulation. This initial response period corresponds to the “highest-gain” of the PNs, before they will be suppressed by the inhibitory currents from the forthcoming AHP-phase.

**Figure 14:**
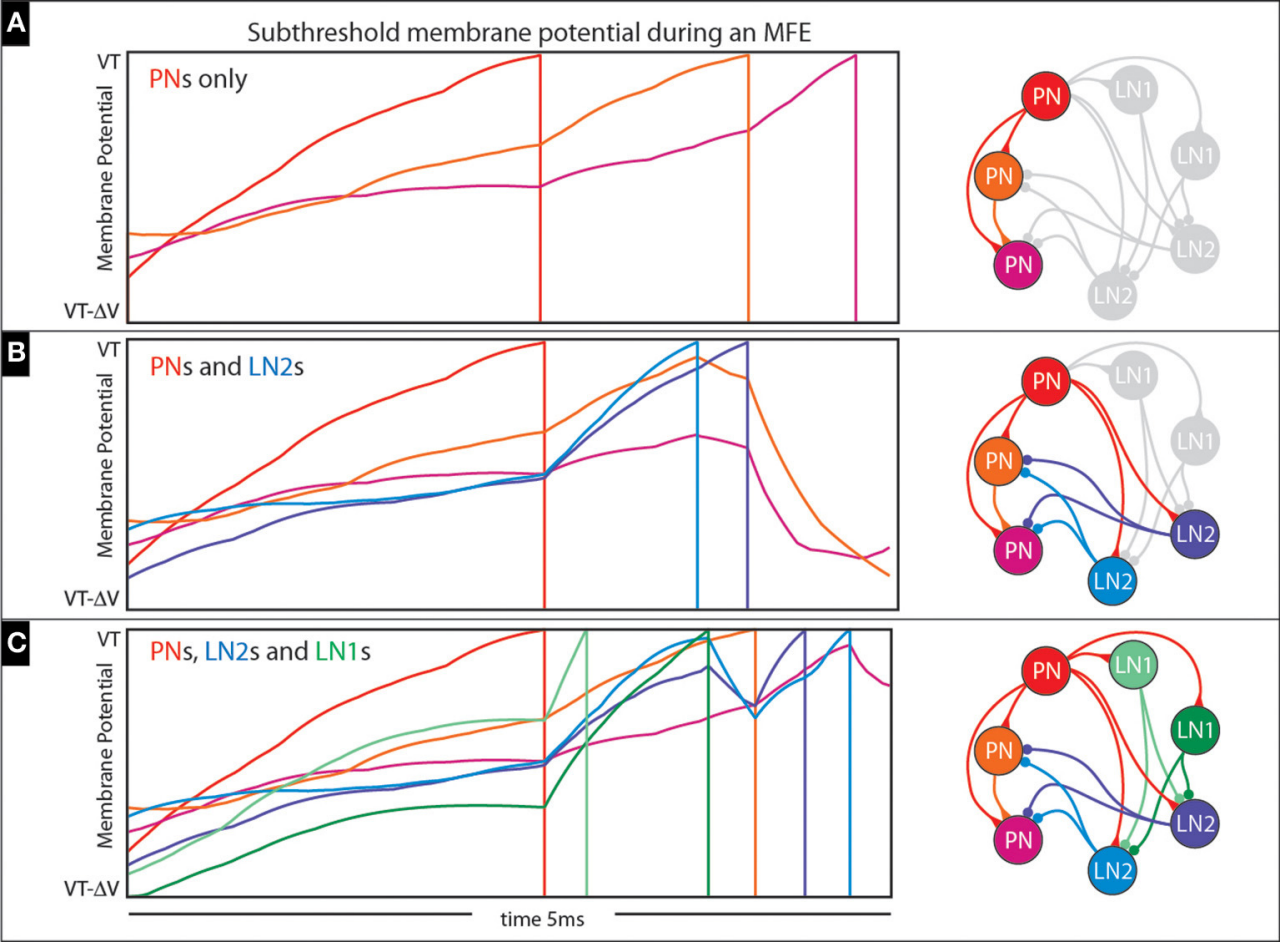
**Illustration of multiple-firing-events (MFEs): (A–C) Here we use a collection of small networks to illustrate the kinds of chain-reactions we see in our simulations**. See text for details.

An example of an MFE within an idealized 3-neuron network is shown in Figure 14A. This network comprises 3 PNs which are stimulated with feedforward Poisson input similar to our full network; the synaptic time constants are also similar to our full network (i.e., 2 ms), but the synaptic coupling strengths are about a factor of 10 higher so that MFEs clearly manifest. On the top left we show a short 5 ms sample trajectory from this network with the subthreshold voltage of each PN color-coded in accordance with the network to the right. Because each neuron is modeled using the integrate-and-fire equations, each subthreshold-voltage will fluctuate (based on its combination of input currents) until it reaches the firing-threshold (VT), at which point the neuron will fire and reset to VR (firing denoted by vertical line), no longer participating in this short stretch of dynamics. Note that the first PN to fire adds an excitatory input current to the second PN and encourages it to fire as well; the combined effects of these first two PNs adds a substantial excitatory presynaptic current to the third PN, causing it to fire less than a millisecond later. This entire cascade takes place over <3 ms, and is similar to the MFEs in our larger network.

Of course, the chain-reactions we've been describing don't just include PNs; the first few PNs to initiate a chain-reaction will also cause firing amongst the LNs, many of which also benefit from the high-gain state. How such a chain-reaction unfolds can be very complicated and situation-dependent; recall that the LN population is heterogeneous. LN1s inhibit LN2s; LN2s inhibit PNs. The short-time-scale dynamics within each MFE can be rather complicated, with PNs, LN1s and LN2s competing over the fate of the cascade.

This complexity is illustrated in Figures 14B,C. In Figure 14B we expand the 3-neuron network of Figure 14A to include two LN2s (see addition blue neurons on the right, as well as bluish trajectories on the left), which affect how the cascade unfolds. This time, the first PN again adds excitatory presynaptic current to the other two PNs, but also to the two LN2s; these LN2s manage to fire before the other PNs would fire, giving rise to inhibitory presynaptic currents which actually prevent these other PNs from firing altogether. Another example is shown in Figure 14C, where the simple network is further expanded to include two LN1s (see additional green neurons on the right, as well as greenish trajectories on the left). This time the first PN to fire causes one of the LN1s to fire second, which actually inhibits and delays the spikes coming from the LN2s. As a result, the LN2s are not capable of completely curtailing the cascade, and one of the remaining PNs manages to fire (compare Figure 14B with Figure 14C).

Thus, the specifics of any given MFE are rather variable: it is possible for a chain-reaction of PN spikes to trigger LN2 spikes which halt the cascade or to trigger LN1 spikes which help the cascade continue via disinhibition. When each MFE concludes—usually due to a barrage of inhibition—the PNs involved experience an abundance of persistent inhibitory currents: both presynaptic and intrinsic. If the system is stimulated, a sufficiently strong feedforward input can override these inhibitory currents and cause further firing. On the other hand, when the network is unstimulated, these inhibitory currents are usually sufficient to prevent further firing.

#### MFEs strongly affect the dynamics of our model

As one can see from the description above, MFEs represent a particular kind of synchrony; they are most decidedly *causal* in nature, stemming from strong and fast competition between synaptic excitation and inhibition. In this regard, MFEs can be viewed as a more complicated version of the “sandpile” cascades discussed in Bak et al. (1987) and later considered in the context of neuroscience by Mirollo and Strogatz (1990), Gerstner and van Hemmen (1993), DeVille and Zheng (2014) and others. We also believe MFEs to be related to the “neuronal avalanches” studied by Plenz et al. (2011) and Beggs and Plenz (2003).

By contrast, MFEs are quite distinct from many other forms of synchrony that have been studied, such as synchrony borne from (i) correlated feedforward inputs, (ii) global fluctuations in firing-rate, (iii) strong sources of synaptic depression, or (iv) synaptic delays. The MFEs we see in our model are not easily characterized analytically as an additional feedforward Poisson spiking process (see Brunel, 2000), or as fluctuations of a balanced state (see van Vreeswijk and Sompolinsky, 1998).

While we don't yet have a full characterization of MFEs ourselves, we have studied them in a less complex network (Rangan and Young, 2013b). Even this simpler case required serious effort to analyze mathematically (Rangan and Young, 2013a; Zhang et al., 2014a,b; Zhang and Rangan, 2015). Thus, at present we eschew any sort of detailed analysis, instead describing in words the dynamic picture we see in our model:

##### MFEs manifest within the control state

**Figure 15:**
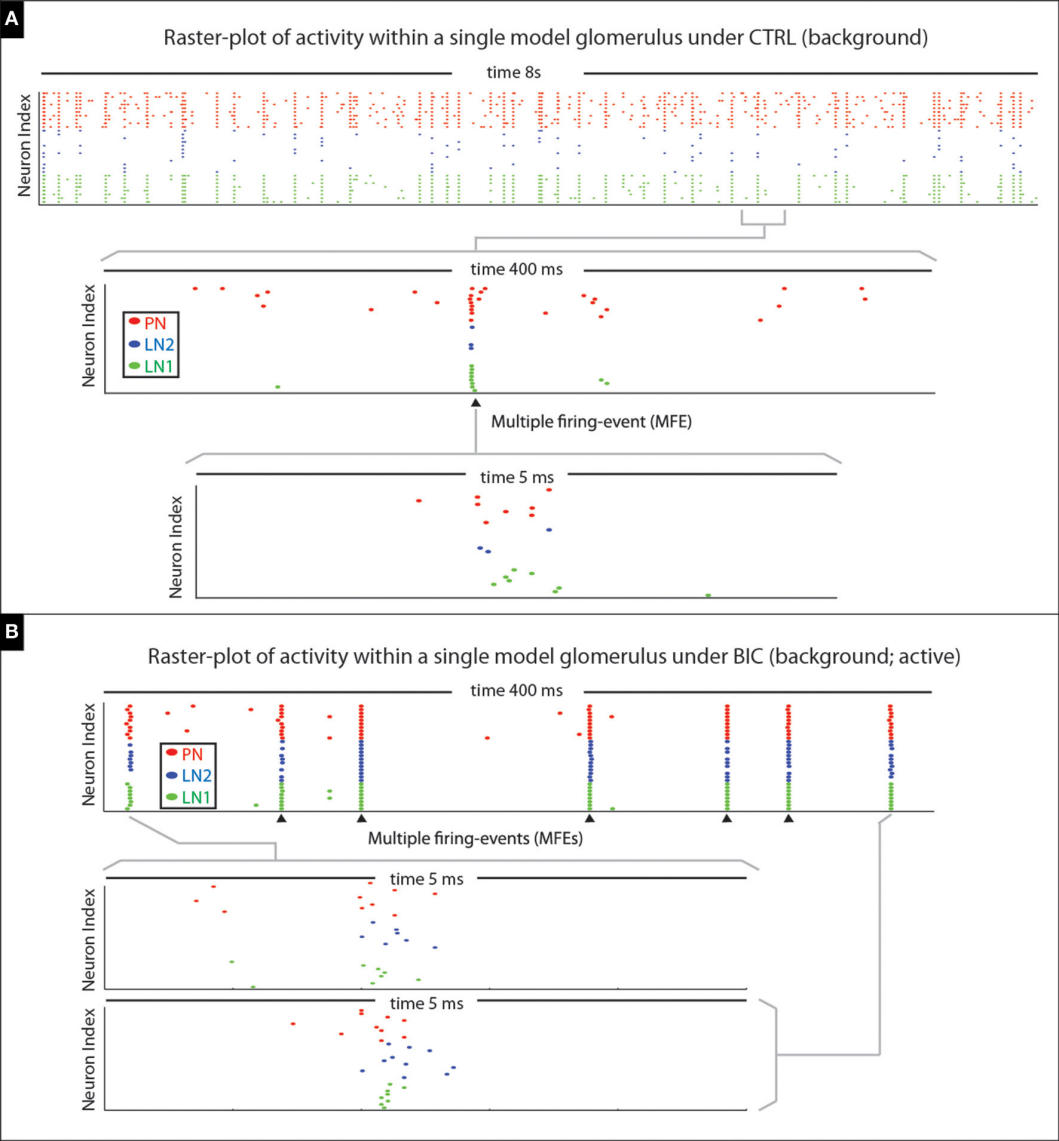
**Illustration of multiple-firing-events (MFEs) within our model network**. **(A)** Here we show a raster-plot of spontaneous activity within a single model glomerulus from our network. The spikes associated with the different kinds of neurons are indicated via different colored dots. By zooming-in we can see an example of a MFE transpiring over ~5 ms. **(B)** Here we show a raster-plot of spontaneous activity spontaneous activity from the same model-glomerulus under the BIC-on condition, with two MFEs displayed in more detail underneath. This raster-plot is taken during an active-epoch, where this glomerulus is firing at roughly 20 Hz. Two MFEs are shown in detail; the rest indicated with arrowheads. Note that the firing-events within each MFE are far from independent, instead occurring in brief synchronous bursts. In each case the MFE is precipitated by the firing of one or more PNs; these first few spikes trigger a cascade which includes more PNs, as well as LN1s and LN2s.

In terms of the model's spontaneous activity, many of the PN firing-events are isolated (i.e., not part of any MFE), but some PN spikes occur synchronously (see Figure 15A). When the model is driven by an odor pulse the activity levels of both the PNs and LNs rise; again the PN activity comprises both MFEs and isolated spikes, although with both occurring at a much higher frequency than in the spontaneous state. Shortly after any odor pulse the PN activity dies down, and the PNs are driven predominantly by persistent inhibitory currents. These inhibitory currents combine slow synaptic inhibition with intrinsic SK-currents, giving rise to an AHP-phase. During this AHP-phase, our model PNs are no longer in a high-gain state, and exhibit very little firing at all (i.e., very few isolated spikes or MFEs).

##### MFEs underlie the PTX-on phenomena

When PTX is applied, the GABA-A coupling strengths are reduced; the LN2 presynaptic inhibitory current drops; the LN2 population moves closer to the spiking threshold; the LN2 firing rate increases significantly; and the net inhibitory presynaptic currents to the PNs increases somewhat. The net effect of all this on spontaneous activity is rather simple: the spontaneous PN firing-rate is somewhat lower in the PTX-on state than in the control state. The excess inhibitory currents lower both the probability of isolated spikes and MFEs.

With regard to stimulated activity, however, the story is more intricate. As we discussed in Figure 14, predominantly excitatory networks (i.e., networks without strong disinhibition, similar to Figure 14A) tend to have more stereotyped MFEs than networks that are capable of strong disinhibition (i.e., networks with strongly competing LN1s and LN2s, similar to Figure 14C). A corollary to this claim is: networks with stronger disinhibition tend to be more variable than networks without. We believe that this mechanism underlies the PTX-on reduction in PN consistency.

To elaborate: recall that the PTX-on state causes the LN2 population to fire more vigorously (i.e., to be “higher gain”) than in background. As a consequence, the competition between the LN1s, LN2s and PNs is more acutely felt when PTX is on. This increased competition means that—for certain choices of parameters—the cascades that occur within any given MFE are even more variable than in the control state. This extra variability gives rise to the PTX-induced reduction in PN consistency across isolated stimulus pulses. As corroborated by numerical experiments, this reduction in PN consistency is accentuated as the overall level of disinhibition rises (see Supplementary Figure S6F). Note that this phenomenon is not captured via a mean field reduction, and thus will not manifest in most standard firing-rate models.

##### MFEs underlie the BIC-on phenomena

Recall that the BIC-on state in our network involves both a reduction of GABA-A coupling strengths as well as a reduction in the intrinsic SK-currents within the PNs. The effect of the former alone would be identical to the PTX-on state. However, the removal of the SK-currents changes the story quite significantly. When the PNs no longer have SK-currents, the conclusion of each MFE no longer heralds the onset of intrinsically generated AHP-currents. As a result, the PNs participating in any one MFE are free to participate in another shortly afterwards, as long as they are not suppressed by inter-glomerular inhibition coming from elsewhere in the AL.

Thus, under BIC it is possible for the network to generate “runaway synchronization,” where any one glomerulus produces a stochastic sequence of MFEs with a characteristic period determined by the feedforward input currents (typically in the 50 ms range). An example of such behavior is illustrated in Figure 15B. During such an MFE-sequence many of the other glomeruli will be suppressed by this active glomerulus (due to strong inter-glomerular inhibition, enhanced by the disinhibitory effects of BIC). This runaway synchronization typically continues until the active glomerulus “falters,” and by chance fails to generate an MFE. At this point another glomerulus—one that was initially suppressed—has a chance to grab the reins and begin its own runaway sequence of MFEs. Such a coup—if it occurs—typically takes place rather abruptly, as the successor only needs a short window of opportunity to nucleate an MFE and take over.

In this manner the spontaneous activity in the BIC-on state can produce—for any given glomerulus—epochs of periodic firing (i.e., when the glomerulus is active) alternating with epochs of quiescence (i.e., when another glomerulus is active). The time-scale of these epochs is determined by the probability that a bout of runaway synchronization “falters.” Depending on the choice of parameters, this falter-probability can be quite small—corresponding to long epoch timescales in the tens of seconds. Our intuition underlying this argument is essentially the same as the discussion in Section 10.6 of Zhang and Rangan (2015), which also presents an analysis of this phenomenon. As before, this BIC-induced phenomenon is not captured by a mean-field reduction.

#### Summary

The narration we have provided above captures—as best we can—the essential dynamical features of our computational model. As described, the architectural features of our network give rise to a dynamical regime with many interdependent mechanisms that interact in a complicated way. Whether or not any of these mechanisms applies to the real AL is an important question, which we turn to now.

### Section R3: model predictions

It is expected that many of the specific dynamical details of our model will vary depending on our exact choice of parameters. In the previous section we attempted to gloss over these minutiae and focus only on the salient features of our model: features that persisted across all the parameters which satisfied our benchmarks. Some of these salient features take the form of emergent dynamical mechanisms; mechanisms that we did not explicitly build into our model, but which arose naturally from the interactions of the network. Such emergent mechanisms give rise to predictions regarding the real AL.

Below are two such predictions; both involve emergent dynamical mechanisms which are integral to the function of our model and robust to our choice of parameters.

1. Our model predicts that PNs in the AL participate in MFEs. While many PN spikes are isolated, many also occur as a result of other spikes. This latter phenomenon can involve just 2 PNs, or (more rarely) all the PNs in a glomerulus, and often includes LN spikes as well. These MFEs are most common during the initial response to a stimulus; but can also occur during spontaneous activity.

We feel quite comfortable with this prediction for two reasons. Firstly, MFEs emerged ubiquitously across a very wide swath of parameters that was much larger than—and included—the region in parameter-space that was biologically plausible. Put another way, our model never did anything even remotely reasonable without MFEs occurring. Secondly, we believe that MFEs are a key ingredient in the dynamical interactions responsible for both the PTX- and BIC-induced phenomena.

2. Furthermore, our model predicts that the BIC-induced structured activity encompasses not just the MGC, but many other glomeruli as well. Any glomerulus with a foreshortened AHP-phase—as induced by BIC—can begin producing MFE sequences. Moreover, different glomeruli compete to produce these MFE-sequences: such activity within any one glomerulus ensures—through interglomerular inhibition—that other glomeruli are suppressed. Conversely, a quiescent epoch observed in any one glomerulus necessarily implies an active epoch occurring someplace else.

We are also comfortable with this second prediction for two reasons. Firstly, despite all of our parameter-scanning, we were never able to produce BIC-induced structured activity without the underlying mechanism of competing MFE-sequences. This held true even when we searched across parameters that didn't even satisfy our other benchmarks. In other words, our model seemed incapable of producing structured activity on a 10 s timescale in any other way. Secondly, we have observed a similar mechanism at work in a model of the primate visual cortex (Rangan and Young, 2013b). In this previous work, competing MFE-sequences of much the same nature give rise to the slowly shifting patterns of activity observed in the anesthetized cortex—a state which, like our BIC-on state, does not exhibit a prolonged AHP and thus also allows for runaway synchronization.

### Section R4: validating the model with further experiments

We now depart from our computational model and return to physiology to try and find evidence confirming our two predictions.

Our first prediction above—i.e., the existence of MFEs—has been observed indirectly under a variety of experimental conditions. For example, Christensen et al. (2000) found that PN activity within *Manduca* was not independent, but rather correlated to varying degrees depending on the stimulus. Later work by Lei et al. (2002) found that PNs in the MGC often fired synchronously (i.e., within 5 ms of one another), with the preponderance of synchronous spikes dependent on the recurrent presynaptic inhibition. The nature of this synchrony was further clarified by Christensen et al. (2003), which confirmed that—like the MFEs we see in our model—the synchronous PN firings observed in experiment could not be attributed to coordinated firing-rate fluctuations coming from the LFP. Finally, recent work by Martin et al. (2013) found that, when stimulated, at least 15% of the PN spikes produced within the *Manduca* MGC were participants in a synchronous event spanning <2 ms and involving at least one other PN.

While these experiments have yet to directly confirm (i) the chain of causality linking one spike to the next and (ii) the participation of LNs, taken altogether they strongly suggest that the MFEs we see in our model might be occurring in the AL. Because we did not use these experiments as benchmarks to constrain the dynamics of our model, we can consider them as a kind of validation of the dynamical picture we discovered. Conversely, we could also view the emergent MFEs from our model as further support for many of the conclusions that have been drawn from this experimental work (see e.g., Martin et al., 2011).

Our second prediction above—i.e., that BIC-induced spontaneous patterns involve many glomeruli—has not yet been confirmed. While structured spontaneous activity has been observed within the MGC (see, e.g., our benchmark Lei et al., 2009), such activity outside the MGC has not yet been reported. We take this step here, measuring from glomeruli outside the *Manduca* MGC under BIC application. These new experiments were able to verify aspects of our second prediction:

#### BIC induces structured spontaneous activity encompassing glomeruli outside the MGC

We moved our recording electrode to the medial portion of AL, and recorded from a plant-odorants (hibiscus oil) responsive neuron. This neuron displayed spontaneous bursting patterns, implying that it was a PN (Lei et al., 2011). We found that, under BIC application, this neuron displayed epochs of fast-periodic-spiking lasting 5–10 s long interspersed with quiescent epochs lasting about 20 s long. As exhibited in Figure 12, the time-scale, distribution of, and transitions between these epochs seem commensurate with those reported within the MGC. These drug-induced changes could be reversed by saline wash.

We view this experimental result as an indication that our reasoning is on the right track. Nevertheless, we have yet to directly confirm that (i) the fast-spiking epochs are due to MFE-sequences, and (ii) that the glomeruli compete antagonistically with one another, with only one glomerulus active at a time. Naturally, we hope to carry out such experiments in the future, further illuminating the mechanisms at work within the AL. For now, however, we take a step back and try and interpret the results we have so far.

## Discussion

This paper describes somewhat of a journey; a trajectory beginning with physiological experiments, passing through the realm of modeling and simulation, and ending back again with more experiments. We started out by measuring the PN dynamics and AHP-phase within the MGC under a variety of pharmacological agents. The drastic differences between PTX- and BIC-application lead us to conjecture that the AHP-phase involved both (a) disinhibition mediated by heterogeneous LN connectivity, as well as (b) intrinsic SK-currents produced by the PNs themselves. To determine whether or not these two mechanisms could, simultaneously, give rise to all the phenomena we observed, we built and benchmarked a computational model. This model highlighted the occurrence of MFEs—a dynamical mechanism that we did not directly observe in experiment, but which emerged naturally from the high-gain state of our biologically plausible model. Using our model as a stepping-stone, we then returned to experiment; confirming at least partially some of the predictions we had regarding the existence of MFEs and their dynamical consequences.

It is certainly possible that our hypotheses are not correct, or that we have omitted an important feature; PTX and BIC could affect a variety of specific targets in different ways. For example, rather than involving SK-channels, BIC could primarily affect the synapses of the LN2s while PTX primarily affected the synapses of the LN1s. Nevertheless, we found that our hypotheses were sufficient to explain the phenomena under study, and we feel comfortable concluding that:

- There exists strong fast recurrent excitatory and inhibitory connectivity throughout the *Manduca* AL—both between and within glomeruli. Moreover, this connectivity is heterogeneous—i.e., different neurons can have very different distributions of pre- and post-synaptic connections. Such heterogeneous connectivity implies that the network is capable of disinhibiting the PNs under the right circumstances.
- While the strong recurrent inhibition described in (a) definitely contributes to the strong AHP-phase of the PNs, the AHP-phase also depends on intrinsic inhibitory currents produced by the PNs themselves.
- The strong fast excitation mentioned above facilitates MFEs—causally linked spikes spanning subsets of neurons within the AL. Rapid sequences of MFEs are typically held in check by the presence of the AHP-phase, which prevents PNs from participating in many successive MFEs.

These conclusions are bolstered, to one degree or another, by various lines of experimental evidence, much of which we have referenced along the way. For example, regarding Conclusion-A, Christensen et al. (1998a) found that PN activity in the *Manduca* AL was modulated by disinhibitory microcircuits involving two GABAergic LNs. Regarding Conclusion-B, we have the results of Lei et al. (2009), which clearly show that BIC-application shortens the AHP-phase. Finally, regarding Conclusion-C, we have an abundance of circumstantial evidence (Christensen et al., 2000, 2003; Lei et al., 2002; Martin et al., 2013), as well as our own BIC-induced results of Figure 12, all of which point toward synchrony within the *Manduca* AL.

Nevertheless, not all experimental observations line up with our conclusions. For completeness we briefly discuss some conflicting evidence.

### Is there disinhibition in the silkworm moth?

A recent study (Fujiwara et al., 2014) probed the inhibitory circuits in the AL of another moth species—the silkworm moth *Bombyx mori*—and concluded that odor stimulation produced both recurrent excitatory and inhibitory currents, with the latter emerging after some time delay and supressing the excitatory phase of subsequent odor-evoked responses. This point itself is not inconsistent with our conclusion-A but is based on a different measurement. They then further examined how PTX-application affected the PN dynamics. Unlike our experiments discussed in Section R1, Fujiwara et al. measured the odor-evoked PN spike counts, instead of measuring the AHP. They reported that PTX-application did increase spike counts, in contrast to our observation that PTX-application lengthened the AHP-phase (Figure 6). From their observations they concluded that PTX-application elevates excitatory currents to the PNs; a conclusion that seems diametrically opposite to our work in Section R1, where we concluded that PTX-application actually enhances the inhibitory currents impinging on the PNs. This discrepancy may be due to the species difference or experimental conditions, but is also likely related to the specifics of the measurement used. We remark that, while our conclusions regarding the role of PTX may differ, both we and Fujiwara et al. agree that PTX does not affect the mean odor-evoked firing-frequency (Figure 10). While we don't yet have resolution to this conundrum, earlier work by Waldrop et al., (1987; Christensen et al., 1998a) points out that direct excitatory, inhibitory and disinhibitory connections may all affect the PNs. Antagonizing GABA-A receptors (i.e., PTX) may produce a syndrome of effects that requires us to consider the entire network.

### Does the AHP-phase combine recurrent and intrinsic currents in the swordgrass moth?

Based on our Conclusion-B, we would predict that the duration of the AHP phase should be positively correlated with increased odor concentration; an increased concentration causes higher LN and PN firing, the former giving rise to more recurrent inhibition, the latter to a stronger intrinsic SK-current. This is indeed the case (Figure 7), and moreover this dose-response relation is disrupted by PTX-application. It's unclear how the duration of the AHP phase could encode odor concentrations, but our data at least suggest that the GABA-A receptor-mediated inhibition is related to the dynamic range of PN's responsiveness. However, in another moth species—the black swordgrass moth *Agrotis ipsilon*—the duration of the AHP (termed Inhibitory Phase) is not correlated with odor concentrations (Jarriault et al., 2009). While we cannot yet explain this discrepancy, we noticed that their data do reveal that the magnitude—rather than the duration—of the AHP is concentration-dependent (Figure 7 in Jarriault et al., 2009). Thus, in summary, while we are reasonably confident of the dynamic picture we have painted for the *Manduca*, the mechanisms may reveal themselves differently in other insect species.

## Summary

If indeed our picture is accurate and the *Manduca* AL does exhibit the mechanisms we propose, we are lead naturally to the grander question: what purpose could they serve? We hypothesize that perhaps the *Manduca* has evolved to excel at certain difficult sensory tasks, such as finding a mate on the wing through a highly dynamic scent plume. One necessary computation for such behavior would be to reliably detect and respond to a faint odor-filament spun across a turbulent breeze.

As a moth flies, it encounters such filaments intermittently, with each brief exposure to pheromone lasting no more than a few 10 s of millisecond, and with subsequent glimpses of the odor separated by several 100 s of millisecond. In this kind of scenario it makes some amount of sense for the AL to be in a very high-gain state; with even the slightest hint of pheromone eliciting a vigorous response. Of course, given the brevity of the stimulus, a firing-rate code seems inefficient, and a temporal code (such as the temporal-binding of odor-specific synchronous subsets) might be much more elegant and efficient (Martin et al., 2011). The high-gain state we predict in this paper is consistent with both of these requirements; producing vigorous synchronous bursts of PN activity in response to brief stimulus pulses.

Carrying this narrative forwards, one can imagine the moth having just encountered one such odor-filament, its MGC responding furiously. At this point the moth's AL is blind to the world; after all, a typical consequence of maintaining such a high-gain state is that—after the initial response—it is very easy for recurrent excitatory connectivity to perpetuate that response, regardless of any new stimulus. It is at this point that the AHP-phase steps in; the recurrent inhibition and intrinsic currents curtailing such a runaway response, and allowing the MGC to “reset” after 100–500 ms. This characteristic AHP-time is not too different from the typical time it might take before the moth bumps into the next odor-filament. At that point the moth's AL will be in a high-gain state once more; fresh and ready to respond vigorously a second time.

In conclusion, our integrated theoretical and empirical approach supports the notion that both recurrent network interactions and intrinsic currents contribute to the dynamical properties of the projection neurons in the antennal lobe. These properties render a high-gain state, which may be an adaptive feature for the animal's olfactory behaviors.

## Author contributions

HL, designed and performed experiments, analyzed data, drafted manuscript; YY, performed experiments and analyzed data; SZ, provided overall support for YY and guided the project partially; AR, built the mathematical model and analyzed its performance, revised manuscript.

## Funding

This work is supported by the National Science Foundation (DMS-2100009 to HL and AR), as well as the National Institute of Health (R01-DC-02751 to John G. Hildebrand), and the National Science and Technology Support Program of China (2015BAD08B01 to YY).

### Conflict of interest statement

The authors declare that the research was conducted in the absence of any commercial or financial relationships that could be construed as a potential conflict of interest.

## Footnotes

^1^Even though we used different methods to introduce PTX and BIC into the AL—bath perfusion vs. multibarrel pressure injection—we do not believe that this difference in procedure could be wholly responsible for the extreme discrepancies we observed in the PN dynamics.

## References

[B1] Abou Tayoun A. N. Li X. Chu B. Hardie R. C. Juusola M. Dolph P. J. (2011). The *Drosophila* SK channel (dSK) contributes to photoreceptor performance by mediating sensitivity control at the first visual network. J. Neurosci. 31, 13897–13910. 10.1523/JNEUROSCI.3134-11.2011 21957252 PMC3758547

[B2] Adelman J. P. Maylie J. Sah P. (2012). Small-conductance Ca^2+^-activated K^+^ channels: form and function. Annu. Rev. Physiol. 74, 245–269. 10.1146/annurev-physiol-020911-153336 21942705

[B3] Anthony N. M. Harrison J. B. Sattelle D. B. (1993). GABA receptor molecules of insects. EXS 63, 172–209. 10.1007/978-3-0348-7265-2_8 7678525

[B4] Assisi C. Stopfer M. Laurent G. Bazhenov M. (2007). Adaptive regulation of sparseness by feedforward inhibition. Nat. Neurosci. 10, 1176–1184. 10.1038/nn1947 17660812 PMC4061731

[B5] Bak P. Tang C. Wiesenfeld K. (1987). Self-organized criticality: an explanation of 1/f noise. Phys. Rev. Lett. 59, 381–384. 10.1103/PhysRevLett.59.381 10035754

[B6] Barbara G. Zube C. Ryback J. Gauthier M. Grunewald B. (2005). Acetylcholine, GABA and glutamate induce ionic currents in cultured antennal lobe neurons of the honeybee, *Apis mellifera*. J. Comp. Physiol. A Neuroethol. Sens. Neural Behav. Physiol. 191, 823–836. 10.1007/s00359-005-0007-3 16044331

[B7] Beggs J. M. Plenz D. (2003). Neuronal avalanches in neocortical circuits. J. Neurosci. 23, 11167–11177. 14657176 10.1523/JNEUROSCI.23-35-11167.2003 PMC6741045

[B8] Belmabrouk H. Nowotny T. Rospars J. P. Martinez D. (2011a). Interaction of cellular and network mechanisms for efficient pheromone coding in moths. Proc. Natl. Acad. Sci. U.S.A. 108, 19790–19795. 10.1073/pnas.1112367108 22109556 PMC3241803

[B9] Belmabrouk H. Rospars J. P. Martinez D. (2011b). A computational model of the moth macroglomerular complex. BMC Neurosci. 12(Suppl. 1):P212 10.1186/1471-2202-12-S1-P212

[B10] Bond C. T. Maylie J. Adelman J. P. (1999). Small-conductance calcium-activated potassium channels. Ann. N.Y. Acad. Sci. 868, 370–378. 10.1111/j.1749-6632.1999.tb11298.x 10414306

[B11] Brunel N. (2000). Dynamics of sparsely connected networks of excitatory and inhibitory spiking neurons. J. Comp. Neurosci. 8, 183–208. 10.1023/A:1008925309027 10809012

[B12] Buckley C. L. Nowotny T. (2011). Multiscale model of an inhibitory network shows optimal properties near bifurcation. Phys. Rev. Lett. 106, 238109. 10.1103/PhysRevLett.106.238109 21770552 PMC3276847

[B13] Chotoo C. K. Silverman G. A. Devor D. C. Luke C. J. (2013). A small conductance calcium-activated K^+^ channel in *C. elegans*, KCNL-2, plays a role in the regulation of the rate of egg-laying. PLoS ONE 8:e75869. 10.1371/journal.pone.0075869 24040423 PMC3769271

[B14] Choudhary A. F. Laycock I. Wright G. A. (2012). γ-Aminobutyric acid receptor A-mediated inhibition in the honeybee's antennal lobe is necessary for the formation of configural olfactory percepts. Eur. J. Neurosci. 35, 1718–1724. 10.1111/j.1460-9568.2012.08090.x 22515321

[B15] Christensen T. A. Hildebrand J. G. (1987). Male-specific, sex pheromone-selective projection neurons in the antennal lobes of the moth *Manduca sexta*. J. Comp. Physiol. A 160, 553–569. 10.1007/BF00611929 3612589

[B16] Christensen T. A. Lei H. Hildebrand J. G. (2003). Coordination of central odor representations through transient, non-oscillatory synchronization of glomerular output neurons. Proc. Natl. Acad. Sci. U.S.A. 100, 11076–11081. 10.1073/pnas.1934001100 12960372 PMC196929

[B17] Christensen T. A. Pawlowski V. M. Lei H. Hildebrand J. G. (2000). Multi-unit recordings reveal context-dependent modulation of synchrony in odor-specific neural ensembles. Nat. Neurosci. 3, 927–931. 10.1038/78840 10966624

[B18] Christensen T. A. Waldrop B. R. Hildebrand J. G. (1998a). GABAergic mechanisms that shape the temporal response to odors in moth olfactory projection neurons. Ann. N.Y. Acad. Sci. 855, 475–481. 10.1111/j.1749-6632.1998.tb10608.x 9929641

[B19] Christensen T. A. Waldrop B. R. Hildebrand J. G. (1998b). Multitasking in the olfactory system: context-dependent responses to odors reveal dual GABA-regulated coding mechanisms in single olfactory projection neurons. J. Neurosci. 18, 5999–6008. 9671685 10.1523/JNEUROSCI.18-15-05999.1998 PMC6793051

[B20] Destexhe A. Sejnowski T. J. (2009). The Wilson-Cowan model, 36 years later. Biol Cybern. 101, 1–2. 10.1007/s00422-009-0328-3 19662434 PMC2866289

[B21] DeVille L. Zheng Y. (2014). Synchrony and periodicity in excitable neural networks with multiple subpopulations. SIAM J. Appl. Dynam. Syst. 13, 1060–1081. 10.1137/130943261

[B22] Fujiwara T. Kazawa T. Sakurai T. Fukushima R. Uchino K. Yamagata T.. (2014). Odorant concentration differentiator for intermittent olfactory signals. J. Neurosci. 34, 16581–16593. 10.1523/JNEUROSCI.2319-14.2014 25505311 PMC6608502

[B23] Galizia C. G. Rössler W. (2010). Parallel olfactory systems in insects: anatomy and function. Annu. Rev. Entomol. 55, 399–420. 10.1146/annurev-ento-112408-085442 19737085

[B24] Gerstner W. van Hemmen J. L. (1993). Coherence and incoherence in a globally coupled ensemble of pulse-emitting units. Phys. Rev. Lett. 71, 312. 10.1103/physrevlett.71.312 10055239

[B25] Hansson B. S. Anton S. (2000). Function and morphology of the antennal lobe: new developments. Annu. Rev. Entomol. 45, 203–231. 10.1146/annurev.ento.45.1.203 10761576

[B26] Hansson B. S. Christensen T. A. Hildebrand J. G. (1991). Functrionally distinct subdivisions of the macroglomerular complex in the antennal lobe of the male sphinx moth *Manduca sexta*. J. Comp. Neurol. 312, 264–278. 10.1002/cne.903120209 1748732

[B27] Heinbockel T. Christensen T. A. Hildebrand J. G. (1999). Temporal tuning of odor responses in pheromone-responsive projection neurons in the brain of the sphinx moth Manduca sexta. J. Comp. Neurol. 409, 1–12. 10363707

[B28] Heinbockel T. Christensen T. A. Hildebrand J. G. (2004). Representation of binary pheromone blends by glomerulus-specific olfactory projection neurons. J. Comp. Physiol. A 190, 1023–1037. 10.1007/s00359-004-0559-7 15378331

[B29] Homberg U. Christensen T. A. Hildebrand J. G. (1989). Structure and function of the deutocerebrum in insects. Annu. Rev. Entomol. 34, 477–501. 10.1146/annurev.en.34.010189.002401 2648971

[B30] Huang J. Zhang W. Qiao W. Hu A. Wang Z. (2010). Functional connectivity and selective odor responses of excitatory local interneurons in *Drosophila* antennal lobe. Neuron 67, 1021–1033. 10.1016/j.neuron.2010.08.025 20869598

[B31] Jarriault D. Gadenne C. Rospars J. P. Anton S. (2009). Quantitative analysis of sex-pheromone coding in the antennal lobe of the moth *Agrotis ipsilon*: a tool to study network plasticity. J. Exp. Biol. 212, 1191–1201. 10.1242/jeb.024166 19329752

[B32] Khawaled R. Bruening-Wright A. Adelman J. P. Maylie J. (1999). Bicuculline block of small-conductance calcium-activated potassium channels. Pflügers Arch. 438, 314–321. 10.1007/s004240050915 10398861

[B33] Kim A. J. Lazar A. A. Slutskiy Y. B. (2015). Projection neurons in *Drosophila* antennal lobes signal the acceleration of odor concentrations. Elife 4:e06651. 10.7554/eLife.06651 25974217 PMC4466247

[B34] Laurent G. MacLeod K. Stopfer M. Wehr M. (1999). Dynamic representation of odours by oscillating neural assemblies. Entomol. Exp. Appl. 91, 7–18. 10.1046/j.1570-7458.1999.00460.x

[B35] Lavialle-Defaix C. Jacob V. Monsempés C. Anton S. Rospars J. P. Martinez D.. (2015). Firing and intrinsic properties of antennal lobe neurons in the noctuid moth *Agrotis ipsilon*. Biosystems 136, 46–58. 10.1016/j.biosystems.2015.06.005 26126723

[B36] Lee D. Su H. O'Dowd D. K. (2003). GABA receptors containing Rdl subunits mediate fast inhibitory synaptic transmission in *Drosophila* neurons. J. Neurosci. 23, 4625–4634. Available online at: http://www.jneurosci.org/content/23/11/4625.full 12805302 10.1523/JNEUROSCI.23-11-04625.2003 PMC6740792

[B37] Lei H. Christensen T. A. Hildebrand J. G. (2002). Local inhibition modulates odor-evoked synchronization of glomerulus-specific output neurons. Nat. Neurosci. 5, 557–565. 10.1038/nn0602-859 12006983

[B38] Lei H. Oland L. A. Riffell J. A. Beyerlein A. Hildebrand J. G. (2010). Microcircuits for olfactory information processing in the antennal lobe of *Manduca sexta*, in Handbook of Brain Microcircuits, eds Gordon M. S. Grillner S. (Oxford University Press), 417–426.

[B39] Lei H. Reisenman C. E. Wilson C. H. Gabbur P. Hildebrand J. G. (2011). Spiking patterns and their functional implications in the antennal lobe of the tobacco hornworm *Manduca sexta*. PLoS ONE 6:e23382. 10.1371/journal.pone.0023382 21897842 PMC3163580

[B40] Lei H. Riffell J. A. Gage S. L. Hildebrand J. G. (2009). Contrast enhancement of stimulus intermittency in a primary olfactory network and its behavioral significance. J. Biol. 8, 21. 10.1186/jbiol120 19232128 PMC2687775

[B41] Martin J. P. Beyerlein A. Dacks A. M. Reisenman C. E. Riffell J. A. Lei H.. (2011). The neurobiology of insect olfaction: sensory processing in a comparative context. Prog. Neurobiol. 95, 427–447. 10.1016/j.pneurobio.2011.09.007 21963552

[B42] Martin J. P. Lei H. Riffell J. A. Hildebrand J. G. (2013). Synchronous firing of antennal-lobe projection neurons encodes the behaviorally effective ratio of sex-pheromone components in male *Manduca sexta*. J. Comp. Physiol. A Neuroethol. Sens. Neural Behav. Physiol. 199, 963–979. 10.1007/s00359-013-0849-z 24002682 PMC3840155

[B43] Matsumoto S. G. Hildebrand J. G. (1981). Olfactory mechanisms in the moth *Manduca sexta*: response characteristics and morphology of central neurons in the antennal lobes. Proc. Roy. Soc. Lond. B 213, 249–277. 10.1098/rspb.1981.0066

[B44] Mbungu D. Ross L. S. Gill S. S. (1995). Cloning, functional expression, and pharmacology of a GABA transporter from *Manduca sexta*. Arch. Biochem. Biophys. 318, 489–497. 10.1006/abbi.1995.1258 7733681

[B45] Mirollo R. E. Strogatz S. H. (1990). Synchronization of pulse-coupled biological oscillators. SIAM J. Appl. Math. 50, 1645–1662. 10.1137/0150098

[B46] Newland C. F. Cull-Candy S. G. (1992). On the mechanism of action of picrotoxin on GABA receptor channels in dissociated sympathetic neurones of the rat. J. Physiol. 447, 191–213. 10.1113/jphysiol.1992.sp018998 1317428 PMC1176032

[B47] Oland L. A. Gibson N. J. Tolbert L. P. (2010). Localization of a GABA transporter to glial cells in the developing and adult olfactory pathway of the moth *Manduca sexta*. J. Comp. Neurol. 518, 815–838. 10.1002/cne.22244 20058309 PMC2920212

[B48] Olsen S. R. Bhandawat V. Wilson R. I. (2007). Excitatory interactions between olfactory processing channels in the drosophila antennal lobe. Neuron 54, 89–103. 10.1016/j.neuron.2007.03.010 17408580 PMC2048819

[B49] Otmakhova N. A. Lisman J. E. (2004). Contribution of *I*_*h*_ and GABA_*B*_ to synaptically induced afterhyperpolarizations in CA_1_: a brake on the NMDA response. J. Neurophysiol. 92, 2027–2039. 10.1152/jn.00427.2004 15163674

[B50] Pedarzani P. McCutcheon J. E. Rogge G. Skaaning Jensen B. Christophersen P. Hougaard C.. (2005). Specific enhancement of SK channel activity selectively potentiates the afterhyperpolarizing current IAHP and modulates the firing properties of hippocampal pyramidal neurons. J. Biol. Chem. 280, 41404–41411. 10.1074/jbc.M509610200 16239218

[B51] Pinault D. (1996). A novel single-cell staining procedure performed *in vivo* under electrophysiological control: morpho-functional features of juxtacellularly labeled thalamic cells and other central neurons with biocytin or Neurobiotin. J. Neurosci. Methods 65, 113–136. 8740589 10.1016/0165-0270(95)00144-1

[B52] Plenz D. Stewart C. V. Shew W. Yang H. Klaus A. Bellay T. (2011). Multi-electrode array recordings of neuronal avalanches in organotypic cultures. J. Vis. Exp. 54:2949. 10.3791/2949 21841767 PMC3211128

[B53] Rangan A. Young L. S. (2013a). Dynamics of spiking neurons: between homogeneity and synchrony. J. Comp. Neurosci. 34, 433–460. 10.1007/s10827-012-0429-1 23096934

[B54] Rangan A. Young L. S. (2013b). Emergent dynamics in a model of visual cortex. J. Comp. Neurosci. 35, 155–167. 10.1007/s10827-013-0445-9 23519442 PMC3766520

[B55] Reisenman C. E. Christensen T. A. Hildebrand J. G. (2005). Chemosensory selectivity of output neurons innervating an identified, sexually isomorphic olfactory glomerulus. J. Neurosci. 25, 8017–8026. 10.1523/JNEUROSCI.1314-05.2005 16135759 PMC1351300

[B56] Saito Y. Takazawa T. Ozawa S. (2008). Relationship between afterhyperpolarization profiles and the regularity of spontaneous firings in rat medial vestibular nucleus neurons. Eur. J. Neurosci. 28, 288–298. 10.1111/j.1460-9568.2008.06338.x 18702700

[B57] Schneiderman A. M. Hildebrand J. G. Brennan M. M. Tumlinson J. H. (1986). Trans-sexually grafted antennae alter pheromone-directed behaviour in a moth. Nature 323, 801–803. 10.1038/323801a0 3774007

[B58] Shang Y. Claridge-Chang A. Sjulson L. Pypaert M. Miesenbock G. (2007). Excitatory local circuits and their implications for olfactory processing in the fly antennal lobe. Cell 128, 601–612. 10.1016/j.cell.2006.12.034 17289577 PMC2866183

[B59] van Vreeswijk C. Sompolinsky H. (1998). Chaotic balanced state in a model of cortical circuits. Neural. Comput. 10, 1321–1371. 10.1162/089976698300017214 9698348

[B60] Villalobos C. Shakkotai V. G. Chandy G. K. Michelhaugh S. K. Andrade R. (2004). SKCa channels mediate the medium but not the slow calcium-activated afterhyperpolarization in cortical neurons. J. Neurosci. 24, 3537–3524. 10.1523/JNEUROSCI.0380-04.2004 15071101 PMC6729743

[B61] Vosshall L. B. Wong A. M. Axel R. (2000). An olfactory sensory map in the fly brain. Cell 102, 147–159. 10.1016/S0092-8674(00)00021-0 10943836

[B62] Waldrop B. Christensen T. A. Hildebrand J. G. (1987). GABA-mediated synaptic inhibition of projection neurons in the antennal lobes of the sphinx moth, *Manduca sexta*. J. Comp. Physiol. A 161, 23–32. 10.1007/BF00609452 3039128

[B63] Warren B. Kloppenburg P. (2014). Rapid and slow chemical synaptic interactions of cholinergic projection neurons and GABAergic local interneurons in the insect antennal lobe. J. Neurosci. 34, 13039–13046. 10.1523/JNEUROSCI.0765-14.2014 25253851 PMC6608333

[B64] Wilson C. J. Goldberg J. A. (2006). Origin of the slow afterhyperpolarization and slow rhythmic bursting in striatal cholinergic interneurons. J. Neurophysiol. 95, 196–204. 10.1152/jn.00630.2005 16162828

[B65] Wilson R. I. Laurent G. (2005). Role of GABAergic inhibition in shaping odor-evoked spatiotemporal patterns in the Drosophila antennal lobe. J. Neurosci. 25, 9069–9079. 10.1523/JNEUROSCI.2070-05.2005 16207866 PMC6725763

[B66] Zhang J. Newhall K. Zhou D. Rangan A. (2014a). Distribution of correlated spiking events in a population-based approach for Integrate-and-Fire networks. J. Comp. Neurosci. 36, 279–295. 10.1007/s10827-013-0472-6 23851661

[B67] Zhang J. Zhou D. Cai D. Rangan A. (2014b). A coarse-grained framework for spiking neuronal networks: between homogeneity and synchrony. J. Comp. Neurosci. 37, 81–104. 10.1007/s10827-013-0488-y 24338105

[B68] Zhang J. W. Rangan A. V. (2015). A reduction for spiking integrate-and-fire network dynamics ranging from homogeneity to synchrony. J. Comp. Neurosci. 38, 355–404. 10.1007/s10827-014-0543-3 25601481

