# Supplementary material for: Intrinsic and Network Mechanisms Constrain Neural Synchrony in the Moth Antennal Lobe
Source: Front Physiol. 2016 Mar 8;7:80. doi: 10.3389/fphys.2016.00080 (PMC4781831; doi:10.3389/fphys.2016.00080)

1 Model Description

The model discussed in the main text is that of a few glomeruli within the *Manduca* antennal lobe (AL). Ultimately, we would like to work towards a model that can both (i) provide a window into real AL dynamics while also (ii) illuminating the underlying mechanisms responsible for this dynamics. The former begs for a dynamically rich model whereas the latter begs for simplicity. Our challenge was to craft a model that was competent on both counts; rich enough not to interfere with biologically relevant mechanisms, yet simple enough to be dissected and analyzed. In keeping with the former, we have built this model out of a network of spiking point neurons, representing individual projection neurons (PNs) and local neurons (LNs). In keeping with the latter, we have made several abstractions (explained further below), and our model is rather idealized, with relatively few parameters.

In addition to the rather broad goals stated above, we also wanted to specifically investigate some of the phenomena we have observed in experiment. To this end, we deliberately structured our network's architecture to include two mechanisms that we propose might be responsible for some of these phenomena:

1. Our LN population is explicitly heterogeneous; different LNs have different distributions of pre- and post-synaptic connectivity, and different pre- and post-synaptic coupling strengths. This heterogeneity may contribute to the picrotoxin (PTX) induced reduction in spontaneous PN activity.

2. Our model also includes intrinsic calcium-dependent small-conductance potassium currents (i.e., SK-currents) within the PNs; these intrinsic currents may contribute to the after-hyperpolarization (AHP) currents observed in these neurons, and their removal may correspond to the effects of bicuculline (BIC) application.

These features have yet to be directly observed within the *Manduca* AL but, as discussed in the main text, there is plenty of circumstantial evidence that warrants their exploration. The model we develop endeavors to probe the limits of what these mechanisms are capable of.

As we will discuss below, even though our network contains many dozens of neurons, it is not overly detailed, and we have included only a few of the most important parameters, such as coupling strengths between neuronal populations, and strengths of intrinsic SK- and feedforward input-currents. Additionally, we have chosen several experimental phenomena which impose further constraints on our model parameters. These phenomena include, but are not limited to, (i) the PTX-induced reduction in spontaneous PN activity, (ii) the BIC-induced shortening of the AHP-phase, (iii) the PTX-induced reduction in PN consistency under drive, and (iv) the BIC-induced emergence of structured spontaneous activity. As we have designed our model with (i) and (ii) in mind (see, e.g., mechanisms 1 and 2 above and the discussion in section R1 of the main text), we expect that our model should be able to reproduce these phenomena across a large range of parameters. On the other hand, we have not deliberately engineered our model to produce (iii) or (iv), and so we expect these latter phenomena to impose significant constraints on our model.

Given the relatively small number of free parameters, it was by no means guaranteed that our model would be able to simultaneously satisfy all of these benchmarks for *any* single choice of parameters. Our principal result, as demonstrated below, is that there are indeed fairly sizable regions in parameter space for which our model exhibits simultaneously all of the phenomena studied, even as some of these phenomena appear to place conflicting demands on the system.

The main features of our model are introduced in sections. 1.1 and 1.2. The first restrictions on the 20 or so parameters that appear in this model are discussed in section 1.3.

1.1 Network architecture, equations and parameters

Our model is intended to represent a few glomeruli within the AL. Each glomerulus corresponds to a 'cluster' of excitatory PNs and inhibitory LNs. There are K clusters in total, with the cluster C_k corresponding to the kth glomerulus. The excitatory and inhibitory cells within each cluster are connected to one another sparsely and randomly. The cells are also sparsely connected to cells in other glomeruli. We will refer to connections within any given cluster C_k as *local* or *short-range* connections, and connections between C_k and $C_{k'}$, $k' \neq k$, as *long-range* connections. The basic architecture of our model is shown in Fig S1. This figure also lists many of the parameters that we use later as a 'reference-point' in parameter-space to illustrate our model's dynamics.

To allow for heterogeneity across the LN population, we model two different subtypes of local neurons: denoted LN1s and LN2s. While all the LNs are inhibitory, the LN1s and LN2s have different distributions of pre- and post-synaptic connections.

Each cluster has N_{PN} excitatory PNs, N_{LN1} inhibitory LN1s and N_{LN2} inhibitory LN2s. In order to focus clearly on causal neuron-neuron interactions we model each neuron using single-compartment leaky integrate-and-fire equations. The state-variables associated with any given neuron are its membrane-potential V; quickly decaying excitatory and inhibitory synaptic potentials $g_{\text{fast}}^{\text{syn},PN}$, $g_{\text{fast}}^{\text{syn},LN1}$, $g_{\text{fast}}^{\text{syn},LN2}$; slowly decaying inhibitory synaptic conductances $g_{\text{slow}}^{\text{syn},LN1}$, $g_{\text{slow}}^{\text{syn},LN2}$ and – in the case of PNs – a slowly decaying inhibitory conductance g^{SK} associated with intrinsic SK-like channels.

The state-variable V of a typical neuron n evolves according to a set of ODEs which can be described as follows:

$$\begin{aligned} \frac{d}{dt}V(t) &= -\frac{1}{\tau_V}\left(V - V^L\right) - g^{\text{SK}}(t)\left(V - V^I\right) - g_{\text{fast}}^{\text{input}}(t)\left(V - V^E\right) \\ &\quad - g_{\text{slow}}^{\text{syn},LN1}(t)\left(V - V^{LN1}\right) - g_{\text{slow}}^{\text{syn},LN2}(t)\left(V - V^{LN2}\right) \\ &\quad - g_{\text{fast}}^{\text{syn},LN1}(t)\left(V - V^{LN1}\right) - g_{\text{fast}}^{\text{syn},LN2}(t)\left(V - V^{LN2}\right) \\ &\quad - g_{\text{fast}}^{\text{syn},PN}(t)\left(V - V^{PN}\right). \end{aligned} \tag{1}$$

In these equations $V^L = 0$, $V^E = V^{PN} = 14/3$, $V^I = V^{LN1} = V^{LN2} - 2/3$ (expressed in non-dimensional units) are reversal potentials associated with the leakage-, excitatory- and inhibitory-currents, and the leakage-timescale $\tau_V = 20$ms (Dayan and Abbott, 2001). The voltage V of neuron n evolves continuously until $V(t)$ crosses a membrane-potential threshold $V^T = 1$, at which point this neuron fires, and its voltage $V(t)$ is reset to $V^L = 0$, and held there for a time $\tau_{\text{ref}} = 2$ms.

The term $g^{\text{SK}}(t)$ models the inhibitory SK-channels. This term is 0 for the LNs, whereas for each of the PNs this term is given by

$$g^{\text{SK}}(t) = W^{\text{SK}} \cdot \alpha^{\text{SK}} \star m_n,$$

where W^{SK} is a constant coefficient determining the strength of the SK-currents and $m_n(t)$ is a sum of delta-functions representing the firing activity of the PN itself. The function $\alpha^{\text{SK}}(t)$ is an alpha-function with instantaneous rise-time and a decay-time of $\tau^{\text{SK}} \sim 400$ms. I.e., $\alpha^{\text{SK}}(t) = \mu(t) \cdot \exp(-t/\tau^{\text{SK}})/\tau^{\text{SK}}$, where $\mu(t)$ is the Heavyside function (1 when $t > 0$, 0 when $t < 0$). The '$\star$' denotes temporal convolution.

The term $g_{\text{fast}}^{\text{input}}$ models the excitatory conductance associated with feedforward input to neuron n (see Eq 5 below). The terms $g_{\text{slow}}^{\text{syn},LN1}$, $g_{\text{slow}}^{\text{syn},LN2}$ model the slow inhibitory synaptic conductance, and the terms $g_{\text{fast}}^{\text{syn},Q}$ models the fast Q-type synaptic conductance, $Q \in \{PN, LN1, LN2\}$.

The slow-inhibitory conductances $g_{\text{slow}}^{\text{syn},LN1}$ and $g_{\text{slow}}^{\text{syn},LN2}$ for the neuron n are given by

$$g_{\text{slow}}^{\text{syn},LN1}(t) = \sum_{n' \text{ of type } LN1} W^{nn'} S_{\text{slow}}^{Q,LN1} \cdot \alpha_{\text{slow}}^{\text{syn}} \star m_{n'}, \tag{2}$$

$$g_{\text{slow}}^{\text{syn},LN2}(t) = \sum_{n' \text{ of type } LN2} W^{nn'} S_{\text{slow}}^{Q,LN2} \cdot \alpha_{\text{slow}}^{\text{syn}} \star m_{n'} \tag{3}$$

where $W^{nn'}$ is a connectivity matrix describing whether or not neuron n' is connected to neuron n; $S_{\text{slow}}^{Q,LN1}$ and $S_{\text{slow}}^{Q,LN2}$ are coupling strengths linking the $LN1$ and $LN2$ populations to the Q-type population; $m_{n'}$ is a sum of delta-functions representing the firing activity of neuron n'; and $\alpha_{\text{slow}}^{\text{syn}}(t)$ is an alpha-function with instantaneous rise-time and a decay-time of $\tau_{\text{slow}}^{\text{syn}} \sim 750$ms.

In a similar fashion, the fast Q-type conductance $g_{\text{fast}}^{\text{syn},Q}$, $Q \in \{PN, LN1, LN2\}$, is given by

$$g_{\text{fast}}^{\text{syn},Q} = \sum_{n' \text{ of type } Q} W^{nn'} S_{\text{fast}}^{QQ'} \cdot \alpha_{\text{fast}}^{Q} \star m_{n'}. \tag{4}$$

The alpha-function $\alpha^Q_{\text{fast}}(t)$ has instantaneous rise-time and a short decay-time of $\tau^Q_{\text{fast}} = 2$ms. Thus, each time a neuron fires, the fast synaptic-conductances create a rapid transient in the voltages of the postsynaptic neurons. These rapid transients are what allows one excitatory neuron to drive multiple other neurons to fire over a short period of time (say, $\lesssim 5$ms).

The coupling strengths $W^{nn'}$ encode the connectivity of the network, n' being the transmitting and n the receiving neuron. We assume that the neurons are sparsely and randomly connected. In terms of details, we draw an independent bernoulli random variable to determine whether each pair of neurons is connected or not. Given a pair of neurons n and n' with types Q and Q' in glomeruli k and k', respectively, the probability that they will be connected is given by $P^{QQ'}_{\delta_{kk'}}$, which only depends on their types and whether or not they are in the same glomerulus.

If two neurons are connected, then we assume that their connection strengths depend only on their types. That is to say, given two connected neurons n and n' of types Q and Q', $W^{nn'}$ will be one of 15 coupling parameters $S^{Q,Q'}_{\text{fast}}$, $S^{Q,LN1}_{\text{slow}}$, $S^{Q,LN2}_{\text{slow}}$. Later on, for brevity, we will refer to $S^{QQ'}_{\text{fast}}$ as $S^{QQ'}$, and $S^{Q,LN1}_{\text{slow}}$ and $S^{Q,LN2}_{\text{slow}}$ as $L^{Q,1}$ and $L^{Q,2}$, respectively.

1.2 Two types of input

We will consider two general classes of input $g^{\text{input}}_{\text{fast}}$ for this system: (i) an input which models the unstimulated or 'background' state of the AL, and (ii) an input which models a 'stimulus' intended to represent an odor pulse of some duration. Both the background drive and the stimulus to neuron-n are modeled by an input of the form

$$g^{\text{input}}_{\text{fast}} = S^Q_{\text{drive}} \cdot \alpha^E_{\text{fast}} \star m^{\text{drive}}_n \;, \text{ with } m^{\text{drive}}_n(t) = \sum_h \delta\left(t - T^{\text{drive}}_{n,h}\right) \tag{5}$$

where $\alpha^E_{\text{fast}} = \alpha^{PN}_{\text{fast}}$ is an alpha-function with instantaneous rise-time and a decay-time of 2ms. The spike-times $T^{\text{drive}}_{n,h}$ are chosen from a Poisson process with rate $\eta^{Q,k}(t)$ that depends on the neuron's type Q and it's glomerulus k. Different neurons, even those with same type in the same glomerulus, receive independent realizations of this Poisson input. In background the rate of this Poisson input is chosen to be constant across all glomeruli k. The stimulus is modeled by increasing the rate of this Poisson input drive to selected clusters. For example, in order to model a pulse of odor that targets only a single glomerulus, we would briefly increase the rate of the input drive to that glomerulus. If, on the other hand, we wished to model a sequence of stimulus pulses that each targeted multiple glomeruli, we would modulate the rate η to those glomeruli with some periodic waveform (e.g., a sinusoid or square-wave). Thus, in general, the drive (i.e., the rate of the Poisson input) is given by a cluster-independent background drive plus a cluster-dependent stimulus-specific drive:

$$\eta^{Q,k}(t) = \eta^Q_{\text{bkg}} + \eta^{Q,k}_{\text{stim}}(t) \,.$$

1.3 Basic constraints from physiology

The following are parameters in our model:

(a) network size (K, N_Q),
(b) sparsity of connections ($P^{QQ'}_{\delta_{kk'}}$)
(c) synaptic coupling strengths ($S^{QQ'}_{\text{fast}}$, $S^{Q,LN1}_{\text{slow}}$, $S^{Q,LN2}_{\text{slow}}$),
(d) strength of the SK-currents W^{SK}.
(e) time-scales τ_{slow} and τ^{SK}.
(f) input (S^Q_{drive}, η^Q_{bkg}, $\eta^{Q,k}_{\text{stim}}$).

Many of these parameters are constrained in one way or another by the physiology and architecture of the real AL as we now discuss.

(a) : With regard to network size, a typical glomerulus in the AL has only $\sim 3-4$ PNs, and $\sim 4-6$ LNs, yet each cell may have several spike-initiation sites (Christensen et al., 1993; Gouwens and Wilson, 2009). To

roughly account for this feature we choose $N_{LN1} = 8$, $N_{LN2} = 12$ and $N_{PN} = 10$, representing a few dozen single-compartment spiking units within each glomerulus. We choose $K = 2 - 6$ for most of our simulations – representing a few of the strongly interconnected glomeruli that might most directly influence the activity within the macroglomerular-complex.

(b) : With regard to connectivity, we assume that neurons are connected randomly and sparsely both within each glomerulus, and across glomeruli. The sparsity of these connections is expected to be neither very sparse nor fully dense. We use network connectivities of about 25% for the post-synaptic distribution of LNs, and about 75% for the post-synaptic distribution of PNs.

(c) : The synaptic strengths are constrained by electrophysiological experiments, which indicate that typical EPSPs and IPSPs are not much larger than 1.0mV (Warren and Kloppenburg, 2014). Thus a typical cell near reset will require at least $\sim 20 - 30$ synaptic excitatory 'kicks' (in quick succession) to be driven over threshold. This constraint places an upper bound on the coupling strengths $S_{\text{fast}}^{QQ'}$. We do not expect the time-integrated slow-inhibitory IPSPs to be much larger than the time-integrated fast IPSPs; this will impose constraints on the $S_{\text{slow}}^{Q,LN1}$ and $S_{\text{slow}}^{Q,LN2}$.

(d) : The SK-currents observed in other animals may be significant, but rarely dominate the dynamics of the subthreshold voltage (see, e.g., Abou Tayoun et al. 2012). We constrain our system by ensuring that W^{SK} is comparable to (or not much bigger than) the slow inhibitory presynaptic currents.

(e) : The time-scale of slow-inhibition in the AL varies from $\sim 300 - 800$ms (Wilson and Laurent 2005). In our model we use $\tau_{\text{slow}} = 768$ms for most of our simulations, but this choice is certainly not critical to our results. If this timescale were to change by $\pm 50\%$, we can easily retune our other parameters to retain the same qualitative behavior. The time-scale of SK-currents is also $\sim 300 - 800$ms (Adelman et al. 2012), and we use $\tau^{\text{SK}} = \tau_{\text{slow}}/2 = 384$ms; again, this choice is not critical to our results.

(f): The input to our system is similarly constrained by experiments. Within the real AL the EPSPs due to input are rarely large, and consist of many micro-EPSPs from the antennal nerve (Liu et al. 2014). When driven, the additional input usually targets the glomeruli associated with the stimulus. Thus, we constrain our system by requiring that (i) the magnitude of S_{drive}^{Q} is small (say, $< S_{\text{fast}}^{Q,PN}$), and (ii) the additional input associated with a cognate odor should only significantly affect one glomerulus.

In the AL it is observed that spontaneous activity usually involves PN firing-rates of 5-15 Hz, with inhibitory firing-rates that are not much higher (Lei at al. 2011). This places a lower bound on the input current to our system. If the combination $S_{\text{drive}}^{Q} \cdot \eta_{\text{bkg}}^{Q}$ is too low, then our system will not fire sufficiently frequently in background.

Last but not least, many different experiments have demonstrated that the AL is sensitive to inputs (Tabuchi et al. 2013). This sensitivity places a constraint on our model, requiring that the background input not be too large (i.e., an upper bound on S_{drive}^{Q}). If S_{drive}^{Q} were to be too large, then the system will not be that sensitive, instead responding linearly as $\eta^{Q,k}$ increases (Brunel, 2000). Thus, we consider values of S_{drive}^{Q} that are relatively small, with $\eta^{Q,k}$ relatively large; each neuron receives many relatively weak input EPSPs. This type of input ensures that the membrane-potential of each neuron is often close to V^T, and that a small additional input can drive many neurons to fire.

2 Benchmarking the Model

How good is the model described above? One way to find out is to try to replicate some of the empirical observations of the AL that are not direct consequences of the modeling, i.e., they are not directly implied by our model architecture or by the rules of the dynamics. The following sections discuss several such empirical observations. In each case, we will demonstrate (via simulations) that the phenomenon occurs for suitable choices of parameters, and give a sense of how they constrain the network. Our aim is to produce regions in parameter space which exhibit all of the phenomena considered. We will often refer to the 'reference-set' of parameters listed in Fig S1 and described more fully in the Appendix.

For brevity, below we will abbreviate $S_{\text{fast}}^{QQ'}$ as $S^{QQ'}$, and $S_{\text{slow}}^{Q,LN1}$ as $L^{Q,1}$ and $S_{\text{slow}}^{Q,LN2}$ as $L^{Q,2}$.

2.1 Sources of noise

We pause here to remark that we have two different sources of randomness in our model. Firstly, the most obvious source of randomness comes from the Poisson input drive to each neuron. Each time we simulate our networks this Poisson input drive could potentially be different. The results we present below in this section are robust to this source of randomness: for any given particular network and choice of parameters we collect our statistics over several hundreds to thousands of simulated seconds of evolution.

The second source of randomness has to do with the network connectivity; the networks we simulate are randomly generated using the connection probabilities $P_1^{QQ'}$ and $P_0^{QQ'}$. Each time we randomly generate a network we'll obtain a different connectivity matrix, and the dynamics of that particular network will be slightly different than other networks generated using the same parameters. Because the number of neurons in each network is not too large (i.e., only 20 per glomerulus), the variation from one randomly generated network to the next is not insignificant. In order to ensure that our results are robust to this source of randomness, we generate (and simulate) many networks for each choice of parameters. While, for any given set of parameters, we may use specific randomly-generated networks to illustrate some of our benchmarks, we base our general conclusions on the average behavior of networks generated using those parameters. Compare, e.g., Figs S3 and S4; the former illustrates the dynamics of a particular network for our particular reference-set of parameters, whereas the latter displays results collected across many randomly generated networks for those parameters (as well as analogous results across many different points in parameter space).

2.2 Control phenomena

Spontaneous activity:

As mentioned above, in the real AL the PN and LN firing-rates are between 5 and 15Hz in background (i.e., when unstimulated). From the point of view of a single neuron, this activity seems sporadic, without obvious structure. To corroborate this point, the inter-spike-interval (ISI) distribution for spontaneous activity is roughly unimodal, with a long tail (Lei et al. 2011). We require that corresponding values for our network lie within these acceptable ranges. In terms of constraints, this benchmark mainly requires that the combination of input strength S^Q_{drive} and rate η^Q_{bkg} not be excessively large.

As we will see later in section 2.4, a secondary constraint emerges when we consider networks with strong recurrent synaptic excitation. For these networks the spontaneous activity can self-organize, and so in order to maintain an unstructured spontaneous state our remaining parameters are constrained to include a strong source of AHP-current (e.g., via a large W^{SK}). That being said, when there is a strong source of AHP-current this constraint is satisfied easily: quantitatively speaking, all the regimes shown in the parameter-sweep of Fig S4 satisfy this constraint.

Driven activity:

When driven by an odor puff, real PNs and LNs typically respond vigorously, with instantaneous firing-rates that can reach or exceed 40-80Hz. This response is characterized by a rapid transient increase in activity, which then remains elevated for the duration of the odor puff. When the odor is removed the PN activity dies down over 20-50ms, and is followed by a relatively quiescent period exhibiting very infrequent spikes. This quiescent period, dubbed the after-hyperpolarization (AHP) phase, lasts somewhere betwen 500ms to 1s, with a duration that typically depends on the stimulus (e.g., on the odor concentration).

In order for our model to reproduce this phenomenon, we obviously need the drive strength S^Q_{drive} and rate η^Q_{stim} to be sufficiently strong, otherwise we do not get a substantial response. In addition, we need our model to produce a sufficiently strong inhibitory current following the response (i.e., an AHP-current). There are two candidates for such a slow current in our network: (a) recurrent synaptic inhibition from the LNs, and (b) intrinsic SK-currents within the PNs. Both of these sources of current persist for several hundred ms. In order for our network to exhibit a physiologically realistic AHP-phase, we need to have either (a) strong connectivity strengths $L^{PN,1}$ and $L^{PN,2}$, or (b) a large contribution W^{SK} from the SK-currents, or a combination of the two.

A further consequence of the AHP-phase is that, when input pulses are presented in sequence, the AHP-currents induced by one stimulus pulse can interfere with – and detract from – the response to the next pulse. This pulse-respose attentuation will be observed when the inter-pulse-interval (IPI) is comparable to or shorter than the typical AHP-length (i.e., $\lesssim$ 1s). In order to produce pulse-response attenuation

comparable to experiment, we need the accumulated AHP-currents to be neither too weak, nor too strong. Thus, in terms of constraints, we must have the combination of (a) $L^{PN,1}+L^{PN,2}$ and (b) W^{SK} be sufficiently large, but not too large. To achieve this benchmark alone, we could do with only one of (a) or (b). However, as we alluded to above, the most physiologically realistic regimes are those that combine moderate $L^{PN,1}, L^{PN,2}$ with moderate W^{SK} (i.e., if W^{SK} is too low within the control-state, then its removal under BIC-application will not significantly change the dynamics).

An example of the driven activty in our network is illustrated in Fig S2, which shows pulse-response attenuation for the reference-set of parameters listed in Fig S1. In this figure it can be seen that, with inter-pulse-intervals of 512ms, the first pulse is roughly 25% larger than the subsequent pulses in the pulse-train. This phenomenon is robust: quantitatively speaking, every set of parameters displayed later on in Fig S4 demonstrates a similar average pulse-attenuation of at least 20% (when the IPI is 512ms).

2.3 PTX-induced phenomena

In the network described above, each neuron-neuron connection is an idealized representation of the interaction between two neurons, say n' and n. This interaction, quantified e.g., by the number $W^{nn'}S^{QQ'}$, is intended to represent several individual synapses between n and n', with the 'stronger' connections involving more synapses. Given that picrotoxin (PTX) is a known GABA-A receptor antagonist, we can model the effects of PTX by assuming that PTX-application incapacitates or blocks three-quarters of the GABA-A type synapses. This translates, in our model, to modeling the effects of PTX by reducing the coupling strengths of the fast inhibitory conductances by 75%, since these coupling strengths correspond to the effects of fast GABA-A type inhibition.

Specifically, we associate with our network a 'PTX-on' state, in which the coupling strengths $S^{Q,LN1}$ and $S^{Q,LN2}$ are reduced by 75% for each postsynaptic type Q. We do not reduce $L^{Q,1}$and $L^{Q,2}$, since these coupling strengths correspond to slower inhibition which is not mediated by GABA-A-type receptors (Anthony et al., 1993; Lee et al., 2003).

Reduction in spontaneous PN firing-rate:

Under PTX-application, the PNs in the real AL reduce their firing-rates by $10\% - 30\%$ or more, from $\sim$ 10Hz to $\sim$ 7Hz or even less. This reduction in firing-rate is accompanied by an increase in the mean PN inter-spike-interval (ISI), as shown in Fig 4 in the main text. We require that similar features hold for our network, as illustrated in Fig S3, which uses the same reference-set of parameters as shown in Figs S1 and S2 and described in the Appendix.

Our observations are that this phenomenon hinges strongly on disinhibition. Given the architecture of our network, we expect this disinhibitory pathway to involve the LN1s driving the LN2s which drive the PNs. In the ideal situation where the LN1s are unaffected by the rest of the network then the PTX-on state should (i) lower the strength of the inhibitory coupling between the LN1s and the LN2s, which should correspond to (ii) a decrease in the strength of the presynaptic inhibitory currents to the LN2s, which (iii) gives rise to an increase in the firing-rate of the LN2s, which (iv) consequently increases the presynaptic inhibitory currents to the PNs, which (v) finally lowers the firing-rate of the PNs. Note that, due to the nonlinearities of neuronal dynamics, step (iii) can lead to step (iv) even though the PTX-on state also reduces the inhibitory coupling strengths from the LN2 to PN populations.

This mechanism of disinhibition is rather robust in our model, and occurs across a large region in parameter space, even extending to situations where the LN1s are affected by the rest of the network (and the clean argument above does not apply). To illustrate this, we take a 2-dimensional 'slice' of parameter-space around the reference-parameters listed in Fig S1, varying two of the coupling-strengths responsible for disinhibition in our network. The first coupling-strength we vary is $S^{LN2,LN1}_{\text{fast}} = S^{2,1}$, which determines how strongly the LN1s suppress the LN2s over short time-scales. The second coupling-strength we vary is $S^{PN,LN2}_{\text{slow}} = L^{PN,2}$, which determines how the LN2s suppress the PNs over long time-scales. For each choice of parameters (i.e., for each point in parameter-space) we simulate many randomly-generated networks, each for 128s, and accumulate statistics across this collection of networks. This network-average is displayed in Fig S4.

As shown in Fig S4, the PTX-induced reduction in PN-firing-rate is observed for a wide range of parameters spanning moderate values of $S^{2,1}$ and $L^{PN,2}$. Evidence of disinhibition is seen here as well; the PTX-induced reduction in PN-firing-rate corresponds to a PTX-induced readjustment in the LN-firing-rates. This readjustment includes both a PTX-induced increase in LN2-firing-rates (as discussed above) as well as

a PTX-induced reduction in LN1-firing-rates. This latter reduction occurs because – for these parameters – the LN1s are affected by both the PNs and LN2s.

In Figure S5 we condense the information in Fig S4, showing the region in parameter-space that satisfies this benchmark. More specifically, the 'valid' region corresponds to parameters for which the average PTX-induced reduction in spontaneous PN firing-rate is at least 15%. This valid region includes not only the center pixel of Fig S4, but also many nearby points in parameter-space.

Reduction in consistency of PN response across isolated stimulus pulses:

When driven by isolated stimulus pulses (i.e., pulses separated by more than the typical AHP-length of 1s or so), PNs within the MGC respond vigorously. As observed experimentally (see Fig 10 in the main text), different stimulus pulses will typically give rise to different levels of PN response. The PN response to the jth-pulse can be quantified using the mean PN instantaneous-firing-rate, denoted by ω_j, calculated by averaging the PN instantaneous-firing-rate over a time-window around the jth pulse. As discussed in (Lei et al., 2009), the calculation of ω_j is largely insensitive to the time-window used, and time-windows of 64ms-1024ms yield nearly identical results.

The average mean PN instantaneous firing-rate $\langle\omega\rangle$ (taken across independent pulses) is roughly 120Hz, and the standard-deviation σ_ω of this instantaneous-firing-rate (again, taken across independent pulses) is roughly 10Hz. As discussed in the main text, PTX-application alters this picture somewhat, maintaining the mean response $\langle\omega\rangle$, but increasing the standard-deviation σ_ω to about 20Hz. That is to say, PTX-application does not significantly affect the mean PN-response across these isolated stimulus-pulses, but does reduce the consistency of this response, with σ_ω^{PTX} significantly higher than σ_ω^{CTRL}.

We require that a similar phenomenon hold within our network model. To this end we again perform numerical simulations, this time driving our network with pulse-trains separated by an IPI of 2048ms. We measure ω_j for each pulse, with each instantaneous-rate calculated across a 512ms window (as in experiment, our results are very robust to the choice of time-window). Once this is done, we measure the mean $\langle\omega\rangle$ and standard-deviation σ_ω of the collection of $\{\omega_j\}$. An example of the dynamics we see is shown in panels A-D of Fig S6, which uses the same reference-parameters used in the previous figures. As you can see from this figure, our model is capable of reproducing the general trend shown in experiment, with the exception being that our standard-deviations σ_ω^{CTRL} and σ_ω^{PTX} are both higher than those observed experimentally – partly due to the fact that our Poisson-driven integrate-and-fire model is less reliable than an actual neuron driven by the antennal nerve.

While the PTX-induced increase in σ_ω shown in Fig S6D is quite significant (i.e., the PTX-induced relative change in σ_ω, calculated via $\delta\sigma = \left[\sigma_\omega^{PTX} - \sigma_\omega^{CTRL}\right]/\sigma_\omega^{CTRL}$ is more than 50%), this value is specific to the particular randomly-generated system we have simulated. Other randomly-generated systems – with the same set of parameters – will have different values for $\delta\sigma$, ranging from $\sim 15\%$ to $\sim 70\%$. This distribution (across randomly-generated networks) is quite broad, significantly more so than the other statistics we have presented so far. Thus, to quantify the typical behavior of our model, we simulated many randomly-generated networks and measured $\delta\sigma$ for each of these networks. We also measured the means $\langle\omega\rangle^{PTX}$ and $\langle\omega\rangle^{CTRL}$ for each network, as well as the PTX-induced relative change in the mean $\delta\langle\omega\rangle = \left[\langle\omega\rangle^{PTX} - \langle\omega\rangle^{CTRL}\right]/\langle\omega\rangle^{CTRL}$. By collecting these measurements across randomly-generated-networks we then calculated the average of the $\delta\sigma$ taken across the various networks and compared this to the average of $\delta\langle\omega\rangle$ taken across the same networks. We classify a point in parameter-space as 'valid' if the average $\delta\sigma$ is greater than the average of $\delta\langle\omega\rangle$. That is to say, a point in parameter-space is valid if the average PTX-induced change in standard-deviation σ_ω is larger than the average PTX-induced change in mean-response $\langle\omega\rangle$. The region-of-validity for our 2-dimensional slice is shown in Fig S6E in dark-red, along with the region-of-validity associated with the previous benchmark (dark-green).

Comparing the two curves in Fig S6E, we see that the region of validity for this benchmark coincides rather strongly with the parameters for which disinhibition manifests. In fact, as shown in Fig S6F, the values of $\delta\sigma - \delta\langle\omega\rangle$ that we observe across our 2-dimensional parameter-slice are highly correlated with the values of δf (i.e., the relative PTX-induced change in firing-rate) that we see in Fig S4C. While we do not have a full analytical explanation for this correspondence, we believe we have a partial explanation, which we discuss at more length in the main text (see section R2).

2.4 BIC-induced phenomena

In the real AL, bicuculline (BIC) is a GABA-A antagonist. In addition, as discussed in section R1 of the main text, we have reason to believe that BIC may also block putative SK-channels within the PNs; SK-channels that partly contribute to the AHP-phase of the PNs. To simulate these effects within our model we define a 'BIC-on' state in which we make two modifications. First, we reduce the coupling strengths $S^{Q,LN1}$ and $S^{Q,LN2}$ by 75% for each postsynaptic type Q. This reduction by itself is identical to the modification made during the PTX-on state (both BIC and PTX are GABA-A antagonists). Secondly, we reduce the strength W^{SK} of the SK-currents from its original value (usually 0.5) down to 0. This reduction simulates the additional affect that BIC might have on SK-currents.

Reduction in pulse-tracking capability

In the real AL it has been observed that BIC-application greatly diminishes the AHP-phase of the PNs, allowing the PNs to sustain their activity for a long period of time after a stimulus pulse (Lei et al., 2009). This effect can be clearly observed when stimulating the AL using a pulse-train with IPI = 512ms. In the control-state the AHP-phase is quite evident, and the excitatory phase of the PN response to any individual pulse is limited to a much shorter time-interval than the IPI; the pulse-train produces a sequence of clearly separated responses which 'track' the pulses themselves. On the other hand, under BIC-application, the AHP-phase is diminished to the point where the excitatory phase of the PN response persists for longer than the IPI of 512ms; the pulse-train produces a persistent excitatory response that extends to encompass all the pulses, no long cleanly tracking the individual pulses.

We require that a similar phenomenon hold in our network. Given a network, we simulate both the control and BIC-on states when driven by 5-pulse-trains with an IPI of 512ms. An example of the dynamics we see is shown in panels A-B of Fig S7, which again uses the same reference-set of parameters used in previous figures. To quantify this phenomenon, we calculate the autocorrelation $A(\tau)$ of the PN-population-response (accumulated over 2ms bins), as shown in Fig S7C. Clearly, the autocorrelation $A^{CTRL}(\tau)$ of the control-response has sharper peaks than the autocorrelation $A^{BIC}(\tau)$ of the BIC-on response. We smooth these curves using a τ-window of 24ms for ease of visualization, producing $\tilde{A}^{CTRL}(\tau)$ and $\tilde{A}^{BIC}(\tau)$, as displayed in Fig S7D. To quantify the observed difference between the control and BIC-on response, we measure the ratio $\Delta = \left|A^{BIC}(\tau) - A^{CTRL}(\tau)\right| / \left|A^{CTRL}(\tau) - \tilde{A}^{CTRL}(\tau)\right|$ (using the L_2-norm in both the numerator and denominator). This numerator of Δ is the difference between the BIC-on- and control-autocorrelation (in this case ~ 10), whereas the denominator (used as a normalization constant) is the difference between the nonsmoothed and smoothed control-autocorrelation (in this case ~ 2.5). The ratio Δ (in this case ~ 4) will be large when A^{BIC} is significantly different than A^{CTRL}, and will be close to 1 when A^{BIC} is not appreciably different from a noisy version of A^{CTRL}. We classify a point in parameter-space as satisfying this benchmark if the average value of Δ (taken over multiple randomly generated networks) is more than 3. The region within our 2-dimensional slice of parameter-space that satisfies this benchmark is shown in Fig S7E.

Structured spontaneous activity

In the real AL it has been observed that BIC-application gives rise to structured spontaneous activity within the PNs. This structured activity alternates between epochs of fast-periodic-spiking and epochs of near total quiescence. The epochs of fast-spiking are characterized by ISI-intervals of $\sim$ 50ms (i.e., 20Hz firing), whereas the quiescent epochs have firing-rates near 0Hz. The epochs can each last for several tens of seconds (Lei et al. 2009).

We require that a similar phenomenon hold in our network. Given a network, we turn BIC on and let the network evolve without stimulus. An example of the dynamics we see is shown in Fig S8.

In this figure we evolve our network without stimulation – i.e., driven only by the background drive. Moreover, to start out with, we restrict our network to having only $K = 2$ glomeruli. In Fig S8A we show the typical spontaneous activity for our reference-network in the control-state. On top we show a 20s trajectory for the system, depicting the PN spike-count (accumulated over 2ms time-windows) for both glomeruli C_1 (black) and C_2 (gray). This 20s trajectory is part of a long 80s trajectory, shown on the bottom of Fig S8A. The PN spike-count within this 80s trajectory has been convolved with a 2s sliding window in order to reveal any slow time-scales within the dynamics (allowing the vertical axis to represent the local firing-rate, which is the 2s-averaged #-of-spikes-per-neuron-per-second). In addition, the activity of the two glomeruli are plotted on top of one another in order to reveal any inter-glomerulus competition (see arrowheads). Clearly, there is

very little structure to this spontaneous activity; no one glomerulus dominates the other for very long, and their activity is not strongly anticorrelated. In Fig S8B we do the same thing again for the BIC-on state; the glomeruli are shown in red (C1) and dark-red (C2). Within this BIC-on state there is competition between the glomeruli. One glomerulus can maintain an active epoch – firing at $\sim 15 - 25Hz$ and dominating the other glomerulus – for several tens of seconds before relinquishing control and entering a quiescent epoch. Moreover, the activity of the two glomeruli is strongly anticorrelated; the second glomerulus takes over when the first becomes inactive. The situation shown in panels-A,B is not limited to networks possessing only $K = 2$ glomeruli, and we see qualitatively similar behavior for larger K as well. An example is given in panels-C,D, where we increase K to 3 (with the other parameters remaining the same as the reference-set used for panels-A,B). Panels C and D show, respectively, 20s trajectories for the control-state and BIC-on state (again convolved with a 2s sliding window to reveal slow time-scales, and with the glomeruli plotted on top of one another to reveal inter-glomerular competition). Similar to before, we see that the BIC-on state allows individual glomeruli to maintain long active epochs of more than 10s, during which the other glomeruli are suppressed and relatively quiescent. Much like in the $K = 2$ case, the BIC-induced activity of any one glomerulus is strongly anticorrelated with the sum of the activity of the other two.

Quantitatively speaking, we characterize this structured spontaneous activity as follows: (i) individual glomeruli must maintain an epoch of active firing (i.e., firing-rate > 10Hz) for at least 2s, and (ii) individual glomeruli must maintain a quiescent epoch (i.e,. firing-rate < 10Hz) for at least 2s. In Fig S8E we show the region in our 2-d slice of parameter space for which this benchmark is achieved for at least 90% of the randomly-generated networks we simulated. Because this benchmark is binary in nature, and not based on a continuous variable, the region of validity for this benchmark is demarcated without linear interpolation.

As mentioned above, in the BIC-on state the firing-rate of any one glomerulus is anticorrelated with the firing-rate of the other glomerulus in the $K = 2$ case, and anticorrelated with the sum of the firing-rates of the other glomeruli in the $K \geq 3$ case. Quantitatively, this anticorrelation is at least -0.8 (or lower) when the firing-rate is calculated using a 256ms time-window, which is significantly larger in magnitude than the analogous correlation computed in the control-state (i.e., < 0.1 in magnitude) . We see this correspondence – between BIC-induced structured activity and anticorrelations across glomeruli – across all the parameters we have simulated, not merely the 2-d slice shown in Fig S8E. Put another way, structured activity and glomerular antagonism seem to go hand in hand; we have never observed the former without the latter. While we have not fully analyzed this phenomenon, we believe we have a partial explanation, which we discuss in more detail in the main text (see section R2).

2.5 Summary of benchmarks

The various benchmarks we have considered, including control-, PTX- and BIC-induced phenomena, are attained across a relatively broad swathe of parameter-space, including the reference-set of parameters shown earlier in Fig S1 and detailed in the Appendix. As an example of the robustness of our model, we have carried out a 2-dimensional scan of parameter-space, illustrating in Fig S9 the region that satisfies all of our benchmarks. The reference-set of parameters is at the center of this 2-dimensional scan (i.e., the center point within this 9×9 array). In our experience tuning this network, we believe that this valid region is of reasonable size: any one or two of our parameters can be adjusted by up to 40% without destroying the picture we have described above, provided that we retune the remaining parameters appropriately. Other parameters which are currently set to 0, such as the long-range excitatatory connectivity fractions P_0^{QE}, can be increased from 0% up to $10 - 15$% without changing our results significantly (again, assuming we retune the remaining parameters).

3 Appendix

The parameters in our model are described in sections 1.1, 1.2 and 1.3. In order to locate viable parameters which satisfied our benchmarks we conducted many parameter sweeps in this high dimensonal space. Our search was somewhat systematic: we began by fixing most of the parameters based on educated guesses, and then varying one or two other parameters that 'competed'. Examples include pitching recurrent excitation against recurrent inhibition (e.g., a combination of $S^{PN,PN} + S^{2,2}$ against a combination of $S^{2,PN} + S^{PN,2}$). By varying two degrees of freedom in parameter-space at a time, we were able to construct 2-d slices such as

Fig S9, and then examine each slice for parameters that were successful. Our simulations spanning these 2-d slices gave us feedback and allowed us to improve our initial guesses (especially when the simulations failed to produce reasonable parameters).

Via these parameter sweeps we were able to find a region in parameter-space that satisfied all our benchmarks. We believe that this valid region is quite large, accomodating variations in most of the parameters of up to ±40% (note that, as observed in Fig S9, this valid set spans a factor of 2 in both the $S^{2,1}$ and $L^{PN,2}$ directions). A specific 'reference-set' of parameters in the interior of this region is fixed and is used in all of our figures above. The precise values of our reference-set are given below.

We conclude by remarking that our search through parameter-space is certainly not exhaustive; we are not claiming that the region we have discovered is the only valid one.

Reference parameter set

Each glomerulus has 20 neurons, distributed via: $N_{LN1} = 8$, $N_{NL2} = 12$, $N_{PN} = 10$. While each glomerulus in the real AL actually has only a handful of LNs and PN (e.g., 3-6 of each type), many of these neurons may have more than one spiking center (Christensen et al., 2001). Our choice of $N_Q \sim 10$ attempts to crudely model each actual neuron using multiple individual single-compartment integrate-and-fire neurons.

We used only $K = 2$ glomeruli for the majority of simulations shown here, but our results (and region of validity) do not change appreciably if we increase K to $3 - 6$. While the actual AL has many more glomeruli (e.g., 45), the modest values of K that we use here may serve as a reasonable approximation to the glomerular network involving the macroglomerular-complex, a network which may only actually involve a few strongly connected glomeruli.

The feedforward input is governed by: $S^{LN1}_{\text{drive}} = 0.017$, $S^{LN2}_{\text{drive}} = 0.0014$, $S^{PN}_{\text{drive}} = 0.0023$, $\eta^{LN1}_{\text{bkg}} = 3800\text{Hz}$, $\eta^{LN2}_{\text{bkg}} = 3400\text{Hz}$, $\eta^{PN}_{\text{bkg}} = 3800\text{Hz}$. The exact values of these terms do not seem to matter as much as their products: i.e., the mean input currents determined by $S^{Q}_{\text{drive}} \cdot \eta^{Q}_{\text{drive}}$ and $S^{Q}_{\text{bkg}} \cdot \eta^{Q}_{\text{drive}}$. We have found that these input currents should not be too large or two small, and should be chosen so that the system is in a fluctuation-driven state (see, e.g., Rangan and Young, 2013). While we can alter these input currents somewhat, if they become too low then we have no firing in background, and if they become too high then the background firing-rate soars well above the physiological observed bounds.

The additional feedforward input during any stimulus is governed by $\eta^{Q}_{\text{drive}}(t)$, which ranges from 0 (no extra stimulus) to roughly η^{Q}_{bkg} (strong stimulus). Loosely speaking, whenever we simulate an input pulse, $\eta^{Q}_{\text{drive}}(t)$ takes the form of a square-wave pulse with a long tail. Specifically, to simulate an odor-pulse presented from time t_{on} to time t_{off}, we set :

$$\begin{aligned} \mu_{\text{on}}(t) &= \mu(t - t_{\text{on}}), \;\; \mu_{\text{off}}(t) = \mu\left(t - t_{\text{off}}\right), \\ \eta^{Q}_{\text{drive}}(t) &= \eta^{Q}_{\text{bkg}} \cdot \left\{\mu_{\text{on}}(1 - \mu_{\text{off}}) + \mu_{\text{off}} \cdot \exp\left(-\left(t - t_{\text{off}}\right)/384\text{ms}\right)\right\}, \end{aligned}$$

with μ the heavyside function (i.e., $\mu(t) = 1$ if $t > 0$ and 0 otherwise). When we simulate multiple pulses we 'reset' $\eta^{Q}_{\text{drive}}(t)$ to a function of the above type at the onset of each pulse, forgetting the exponential decay from all the previous pulses. Following this procedure, the feedforward drive to any neuron is never more than double the background; reaching its peak during the 'on-period' of each stimulus pulse, and remaining elevated for half a second or so after each pulse ends. This simple behavior is intended to crudely model the saturation and decay of the olfactory sensory neurons within the antenna (Hallem and Carlson, 2006)

The local network architecture is determined by the connectivity fractions $P_1^{QQ'}$ and $P_0^{QQ'}$, as well as the connection strengths $S^{QQ'}$ and $L^{QQ'}$, as shown in Fig S1.

The synaptic time-constants are given by $\tau^{\text{syn}}_{\text{fast}} = 2\text{ms}$, $\tau^{\text{SK}} = 384\text{ms}$, $\tau^{\text{syn}}_{\text{slow}} = 768\text{ms}$, however our results do not change appreciably if these time-constants are altered by a factor or 2 or so.

Finally, the strength of the SK-currents – determined by W^{SK} – is chosen to be 0.5; this choice is made so that the SK-currents are roughly the same order of magnitude as the slow inhibitory synaptic currents. As with many of our other parameters, we can change W^{SK} by a factor of two without appreciably changing our results (provided that we alter the other parameters slightly to compensate).

References

[1] Dayan P. and Abbott L.F.. (2001). *Theoretical Neuroscience.* MIT press, Cambridge, MA.

[2] Brunel N. (2000). Dynamics of sparsely connected networks of excitatory and inhibitory spiking neurons. *J. Comp. Neurosci.* 8:183–208.

[3] Abou Tayoun, A.N., Li, X., Chu, B., Hardie, R.C., Juusola, M., and Dolph, P.J. (2011). The Drosophila SK channel (dSK) contributes to photoreceptor performance by mediating sensitivity control at the first visual network. *J Neurosci.* 31, 13897-13910. doi: 10.1523/JNEUROSCI.3134-11.2011.

[4] Adelman, J.P., Maylie, J., and Sah, P. (2012). Small-conductance Ca2+-activated K+ channels: form and function. *Annu.Rev.Physiol.* 74, 245-269. doi: 10.1146/annurev-physiol-020911-153336

[5] Anthony, N.M., Harrison, J.B., and Sattelle, D.B. (1993). GABA receptor molecules of insects. *EXS.* 63, 172-209.

[6] Christensen, T.A., Waldrop, B.R., Harrow, I.D., and Hildebrand, J.G. (1993). Local interneurons and information-processing in the olfactory glomeruli of the moth Manduca sexta. *J.Comp.Physiol.* A 173, 385-399.

[7] Gouwens, N.W., and Wilson, R.I. (2009). Signal Propagation in Drosophila Central Neurons. *J Neurosci.* 29, 6239-6249.

[8] Lee, D., Su, H., and O'Dowd, D.K. (2003). GABA receptors containing RDL subunits mediate fast inhibitory synaptic transmission in Drosophila neurons. *J Neurosci.* 23, 4625-4634.

[9] Lei, H., Reisenman, C.E., Wilson, C.H., Gabbur, P., and Hildebrand, J.G. (2011). Spiking patterns and their functional implications in the antennal lobe of the tobacco hornworm Manduca sexta. *PLosONE.* 6, e23382. doi: 10.1371/journal.pone.0023382.

[10] Lei, H., Riffell, J.A., Gage, S.L., and Hildebrand, J.G. (2009). Contrast enhancement of stimulus intermittency in a primary olfactory network and its behavioral significance. *Journal of Biology.* 8, 21. doi:10.1186/jbiol120.

[11] Liu, H., Zhou, B., Yan, W., Lei, Z., Zhao, X., Zhang, K., and Guo, A. (2014). Astrocyte-like glial cells physiologically regulate olfactory processing through the modification of ORN-PN synaptic strength in Drosophila. *Eur.J.Neurosci.* doi:10.1111/ejn.12646.

[12] Tabuchi, M., Sakurai, T., Mitsuno, H., Namiki, S., Minegishi, R., Shiotsuki, T., Uchino, K., Sezutsu, H., Tamura, T., Haupt, S.S., Nakatani, K., and Kanzaki, R. (2013). Pheromone responsiveness threshold depends on temporal integration by antennal lobe projection neurons. *Proc Natl Acad Sci.* 110, 15455-15460. doi/10.1073/pnas.1313707110.

[13] Warren, B., and Kloppenburg, P. (2014). Rapid and slow chemical synaptic interactions of cholinergic projection neurons and GABAergic local interneurons in the insect antennal lobe. *J Neurosci.* 34, 13039-13046. doi: 10.1523/JNEUROSCI.0765-14.2014.

[14] Wilson, R.I., and Laurent, G. (2005). Role of GABAergic inhibition in shaping odor-evoked spatiotemporal patterns in the Drosophila antennal lobe. *J.Neurosci.* 25, 9069-9079. DOI:10.1523/JNEUROSCI.2070-05.2005.

[15] Christensen T.A., D'Alessandro G., Lega J and Hildebrand J.G. (2001). Morphometric modeling of olfactory circuits in the insect antennal lobe: I. Simulations of spiking local interneurons. *Biosystems.* Julâ€“Aug; 61(2-3): 143â€“153. PMCID: PMC2773206, NIHMSID: NIHMS45085.

[16] Rangan A.V., Young L.S. (2013). Dynamics of spiking neurons: between homogeneity and synchrony. *J. Comput. Neurosci.* 34:443-460. DOI 10.1007/s10827-012-0429-1.

[17] Hallem E.A., Carlson J.R. (2006). Coding of odors by a receptor repertoire. *Cell.* Apr 7;125(1): 143-60.

Figure 1: Illustration of computational network architecture. On the far left we illustrate the anatomy of a single glomerulus, comprising $N_{PN} = 10$ PNs, $N_{LN1} = 8$ LN1, and $N_{LN2} = 12$ LN2s, each modeled using single-compartment integrate-and-fire equations. These neuronal populations are connected together sparsely via local intra-glomerular connections, determined using sparsity coefficients $P^{QQ'}_{\delta_{kk}} = P^{QQ'}_{1}$. For neurons that are indeed connected together, their fast- and slow-synaptic coupling strengths are given by $S^{QQ'}_{\text{fast}}$ and $S^{QQ'}_{\text{slow}}$, respectively (abbreviated as $S^{QQ'}$ and $L^{QQ'}$ in the text). Representative connections are illustrated in this diagram, with the superscripts P, 1 and 2 standing in for PN, $LN1$, and $LN2$, respectively. The thickness of these connections corresponds roughly to their relative strength. In the middle we illustrate our full network, comprising K glomeruli (e.g., $K = 6$). The neuronal populations within each of these glomeruli are connected together sparsely via long-range inter-glomerular connections; these long-range connections have the same synaptic strength, but are randomly connected using different sparsity coefficients $P^{QQ'}_{\delta_{kk'}} = P^{QQ'}_{0}$. The thickness of these connections also corresponds roughly to their relative strengths; the excitatory long-range connections are illustrated with dashed lines because – for the majority of the simulations we will show later – $P^{QE}_{0} = 0$. On the far right we list some representative coefficients corresponding to a point in parameter space that satisfies our benchmarks. This 'reference-point' will be used later in the figures to follow. When displaying these tables we condense $S^{QQ'}_{\text{slow}}$, which does not involve presynaptic PNs, and $P^{QQ'}_{0}$, $P^{QQ'}_{1}$, which do not differentiate between the subtypes LN1 and LN2. As one can see by examining these tables, the largest coupling strengths are 0.037, from the $LN1 \to LN2$ population, and 0.022, from the $LN2 \to PN$ population; both of which correspond (in our nondimensional units) to IPSPs of roughly $1/40^{\text{th}}$ of the distance from reset to threshold (i.e., $\sim 0.5mV$). The coupling strength from the $PN \to PN$ population is numerically smaller (i.e., only 0.0075), but actually corresponds to a comparable EPSP of $\sim 0.35mV$ (i.e., roughly $1/57^{\text{th}}$ of $\left|V^T - V^L\right|$), simply because the excitatory reversal potential of $V^E = 14/3$ is so much farther away from $\left(V^L, V^T\right)$ than the inhibitory reversal potential of $V^I = -2/3$. We remark that the other coupling strengths, while smaller than the ones we just discussed, are not negligible; if we were to change any of these listed coupling strengths by $\pm 50\%$, our dynamical regime would change slightly, and we would have to re-adjust the other coupling strengths to maintain our benchmarks. A similar story holds for the sparsity coefficients $P^{QQ'}_{1}$, $P^{QQ'}_{0}$, which each can be increased or decreased by 10-15% without changing the dynamical regime significantly (provided, of course, that we readjust our other parameters to maintain our benchmarks).

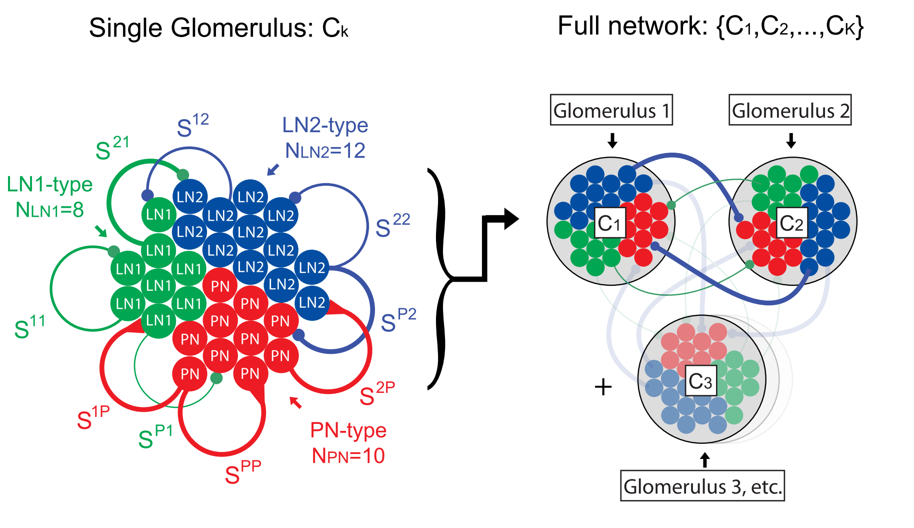

$S^{QQ'} = S^{QQ'}_{\text{fast}} \times 1000$

to \ from	LN1 (1)	LN2 (2)	PN (P)
LN1	7.5	7.5	5.5
LN2	37	7.5	5.5
PN	0.5	22	7.5

$L^{QQ'} = S^{QQ'}_{\text{slow}} \times 1000$

to \ from	LN1 (1)	LN2 (2)
LN1	1.25	1.25
LN2	40	1.25
PN	5.0	35

$P^{QQ'}_{1}$ (%)

to \ from	LN	PN
LN	25	75
PN	38	75

$P^{QQ'}_{0}$ (%)

to \ from	LN	PN
LN	0	0
PN	55	0

Figure 2: Here we illustrate the pulse-response-attenuation for a particular instantiation of our system. We construct a network by randomly choosing a synaptic connectivity matrix (see section 1.1) and then simulate that network for many seconds. The parameters used are those of the 'reference-point' shown in Figs S1 and further detailed in the Appendix. For these simulations we stimulate one of the glomeruli over and over again with 5-pulse-trains (i.e., sequences of 5 repeated pulses), allowing the system to return to spontaneous activity between each pulse-train. Within each 5-pulse-train the duration of each pulse is 128ms, and the interpulse interval (IPI) is either 512ms (left hand side) or 2048ms (right hand side). On the top we show the PN-population spike-count for this glomerulus for one such pulse-train (spike-count accumulated over 2ms time-windows; each pulse plotted on a different row). On the bottom we show the average PN-response for this glomerulus as a function of pulse-number (averaged over 100 pulse-trains). Note that when the IPI is 512ms the pulse-response attenuates by roughly 25% after the first pulse. We remark that these results are specific to this particular glomerulus within this particular network; if we were to rebuild our model by constructing a different random connectivity matrix (using the same sparsity fractions $P_1^{QQ'}$ and $P_0^{QQ'}$) we would end up with slightly different results. Nevertheless, the general trend observed here is quite robust. As mentioned in the text, we observe an average pulse-response-attenuation of at least 20% across the entire 2-d parameter-slice shown later in Fig S4.

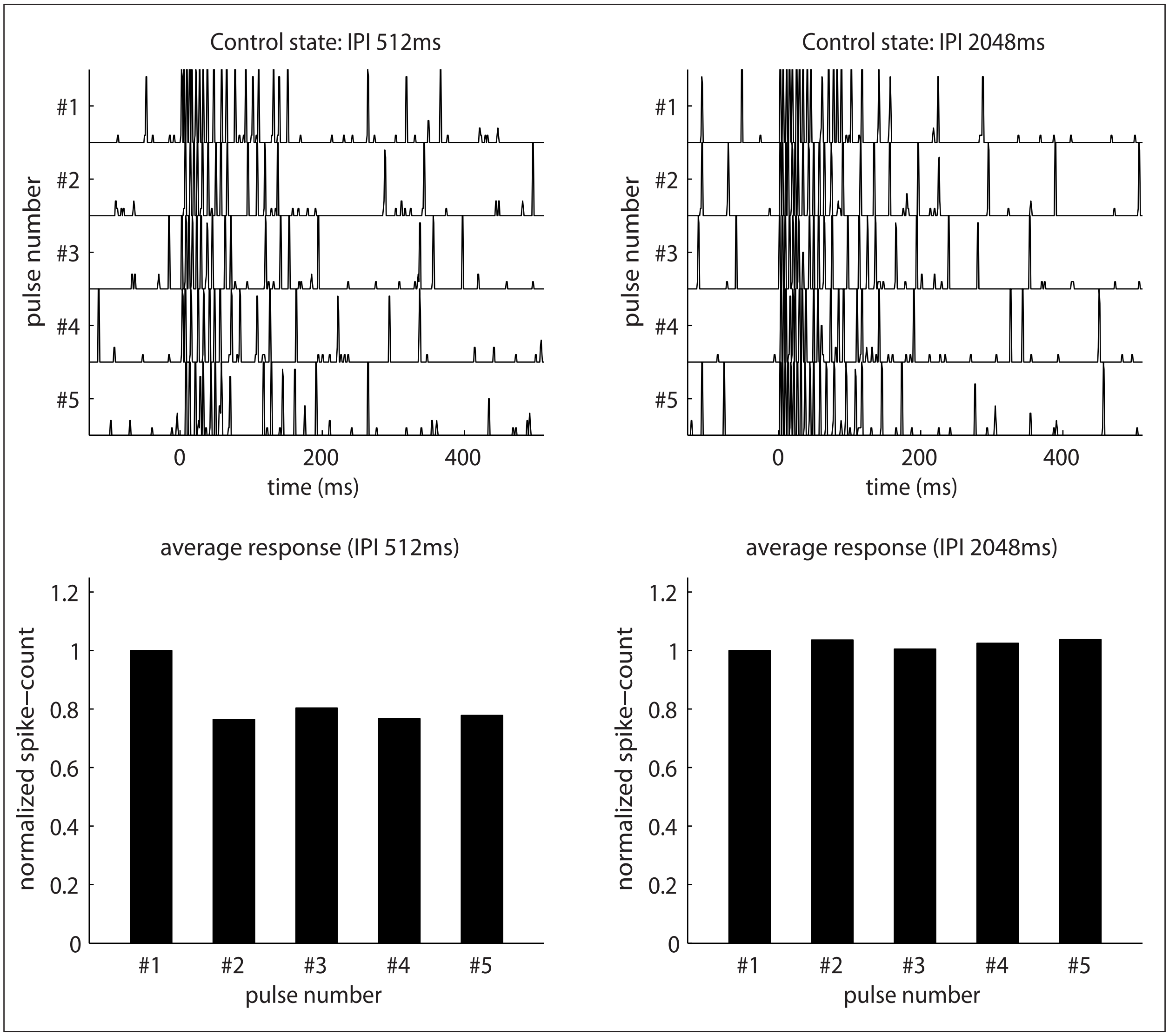

Figure 3: Here we illustrate the PTX-induced reduction in spontaneous activity for the same network show in Fig S2. On the top we show a 10s raster-plot for the PNs in a sample glomerulus of this system (when unstimulated), as well as a 10s trace of the PN-spike-count (accumulated over 2ms bins) for that same glomerulus. In the middle we show a sample raster-plot and spike-count-trace for that same glomerulus when PTX is on. Below that we show the time-averaged firing-rates (averaged over 1s bins) corresponding to the dynamics above. Note that the PN-firing-rate is lower when PTX is on, even though PTX-on corresponds to a reduction in fast-inhibition. This reduction in PN-firing-rate is quantified – for this particular system – in the lower-left (average taken over 128s of simulated time). In the lower-right we show the cumulative-distribution-function for the PN inter-spike-intervals (again, for this particular system) under the control-state (black) and PTX-on state (red). Note that these results are specific to this particular system (and its choice of synaptic connectivity matrix). Nevertheless, as illustrated later in Fig S4, the qualitative behavior will almost always be the same as that shown here.

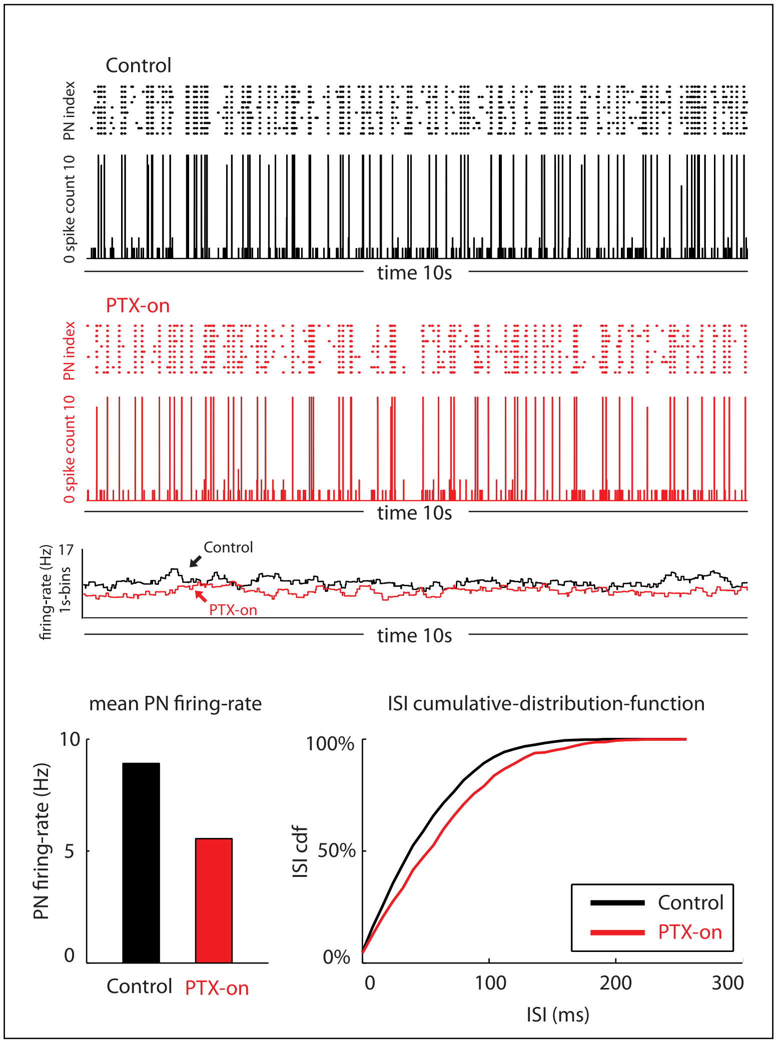

Figure 4: Here we show 9 panels, each describing a different feature of our model across the same 2-dimensional 'slice' of parameter-space. Within each panel, we vary the coupling-strength $S^{2,1}$ logarithmically along the vertical-axis, and the parameter $L^{PN,2}$ logarithmically along the horizontal-axis. The center of each panel corresponds to the reference-point in parameter-space described in Fig S1 (as well as the Appendix), and the borders of each panel correspond to a four-fold decrease/increase in the parameters $S^{2,1}$ and $L^{PN,2}$. For this figure, each pixel in each panel corresponds to a measurement of not merely one 128s simulation, but rather an average taken over several dozen simulations – each with a different randomly generated connectivity matrix. Consequently, the results shown in this figure can be taken as representative of the typical behavior of our model. Panel A, shows the mean spontaneous PN firing-rate under the control condition. Panel B shows the mean spontaneous PN firing-rate under the PTX-on condition. Panel C shows the difference between these two. Panels D,E,F illustrate the analogous firing-rates for the LN1 population, and Panels G,H,I the same for the LN2 population.

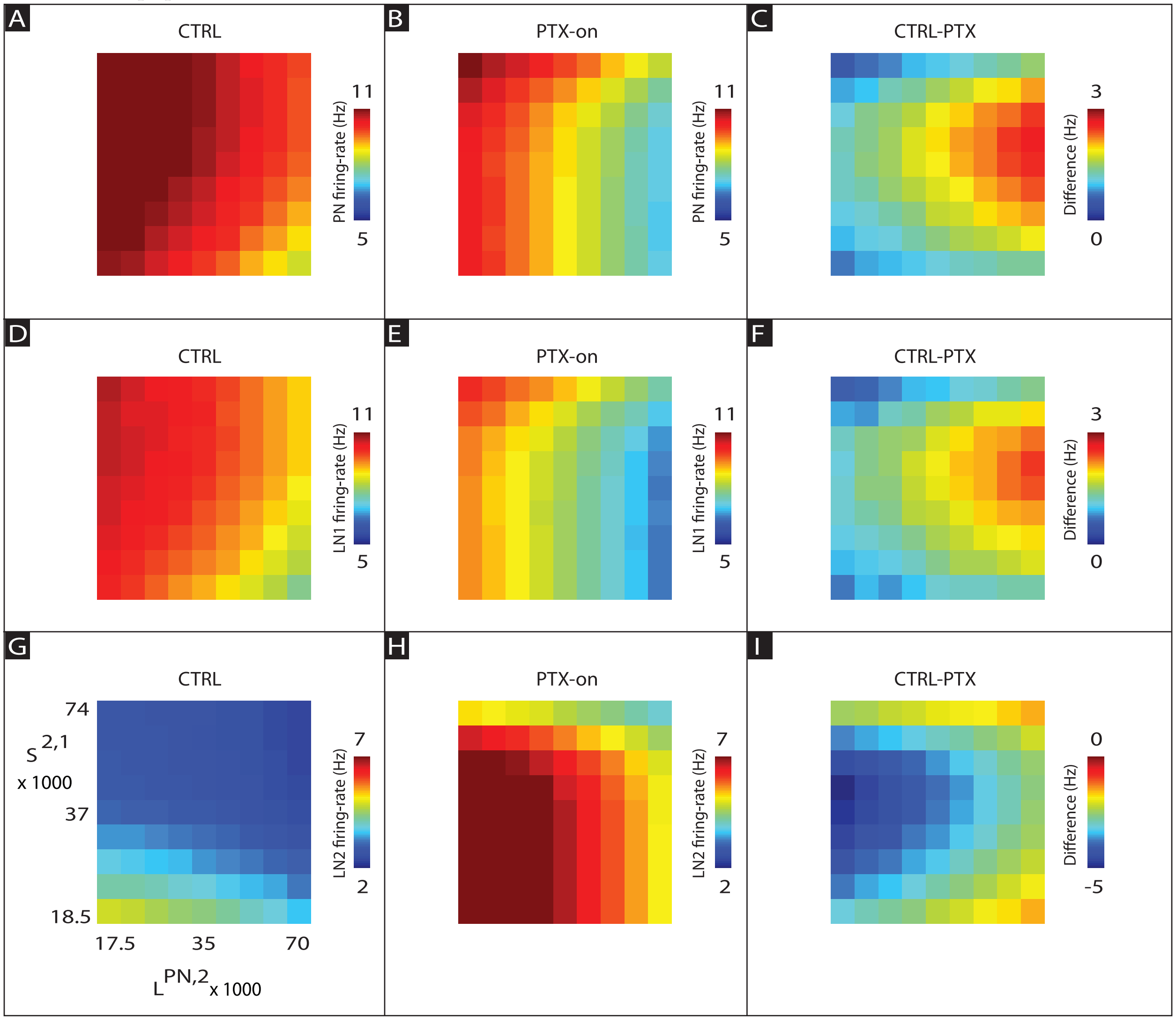

Figure 5: The region within the 2-dimensional slice of parameter-space for which the typical PTX-induced reduction in spontaneous PN firing-rate (as averaged over many randomly-generated instantiations of our network) is at least 15%. The contour delineating the boundary of this region is determined by linearly interpolating data measured on the 9×9 grid of simulations shown in Fig S4. Specifically, we calculate $\delta f = (\text{CTRL} - \text{PTX})/\text{CTRL}$ via S5C/S5A, and then threshold this δf at at the 0.15 level. We show this contour twice: on the left overlaying the panel in Fig S5C, and on the right indicating the region of validity for this benchmark.

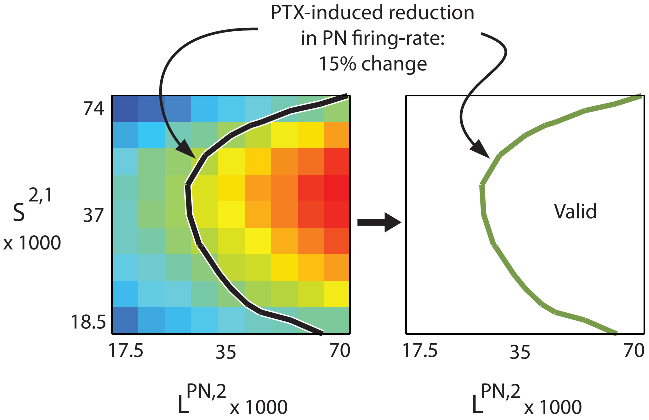

Figure 6: Here we illustrate the PTX-induced decrease in consistency (i.e., increase in σ_ω) using the same reference-set of parameters used in previous figures. For this benchmark we stimulate our network with 128ms pulses, separated by an IPI of 2048ms. In panels A and B we show two 12s system trajectories, one for the control-state and one with PTX-on, each depicting the PN-spike-count for the stimulated glomerulus (spike-count taken over 2ms time-bins). In panels C,D we show the average PN instantaneous firing-rate and the standard-deviation in the PN instantaneous firing-rate (statistics taken across more than 100 pulses). The results shown in panels A-D are specific to this particular network. In panel-E we return to our 2-dimensional slice of parameter-space and show (dark red) the region for which the PTX-induced increase in σ_ω is significant (see text). We overlay on this dark-red contour the dark-green contour of Fig S5 indicating the region for which PTX induces a reduction in spontaneous PN firing-rate. In panel-F we show the correlation between our metric for this particular benchmark (i.e., $\delta\sigma - \delta\langle\omega\rangle$ shown on the y-axis) versus our metric for the previous benchmark (i.e., computed from Fig S4 as $\delta f = (\text{CTRL} - \text{PTX})/\text{CTRL}$)).

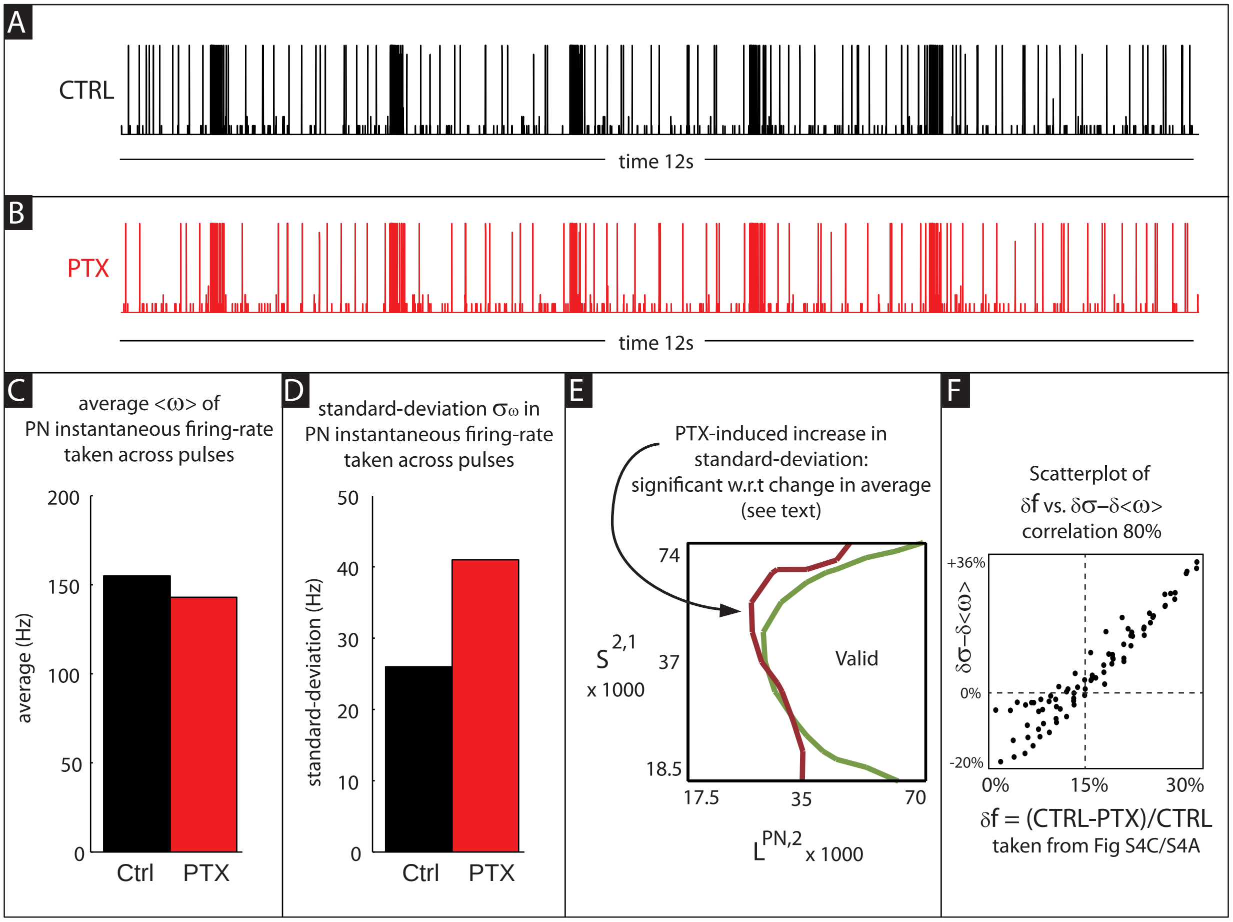

Figure 7: Here we illustrate the BIC-induced decrease in pulse-tracking capability using the same reference-set of parameters used in previous figures. For this benchmark we stimulate our network with 5-pulse trains, each pulse of duration 128ms and separated by an IPI of 512ms. In panels A and B we show two 4s system trajectories, one for the control-state and one with BIC-on, each depicting the PN-spike-count for the stimulated glomerulus (spike-count taken over 2ms time-bins). In panels C,D we show the nonsmoothed and smoothed autocorrelation of this PN-spike-count (statistics taken across several hundred pulses). The results shown in panels A-D are specific to this particular network. In panel-E we return to our 2-dimensional slice of parameter-space and show (dark teal) the region for which BIC induces a significant reduction in pulse-tracking capability (see text). We overlay on this dark-teal contour the dark-red and dark-green contours of our earlier benchmarks. The valid region lies to the right of the dark-teal-contour and to the left of the other contours.

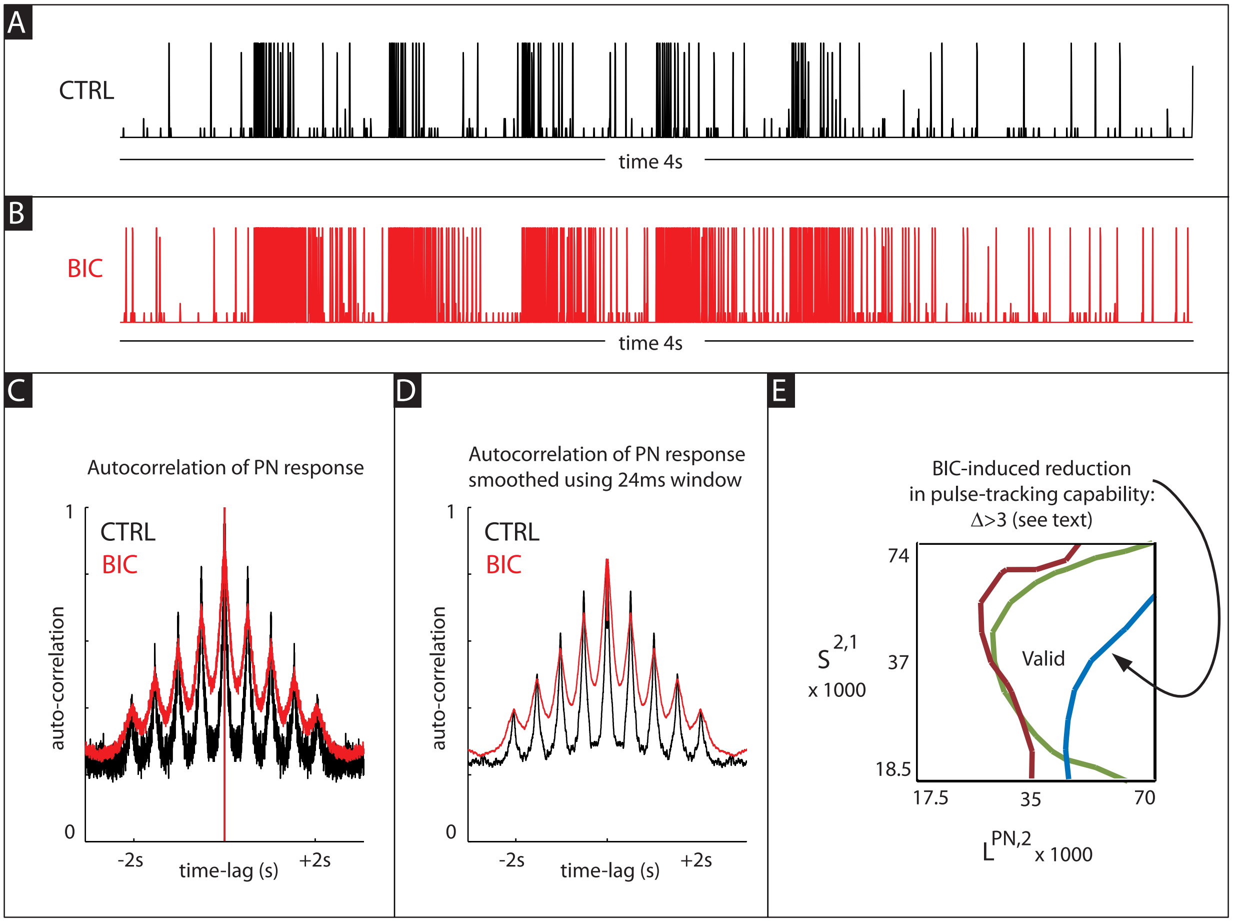

Figure 8: Here we illustrate the BIC-induced structured spontaneous activity. See text for details. The results shown in panels A-D are each specific to a particular network. In panel-E we return to our 2-dimensional slice of parameter-space and show (black) the region for which BIC induces structured spontaneous activity. We overlay on this black boundary the contours associated with our earlier benchmarks. The valid region lies to the right of the dark-teal contour and to the left of the other contours.

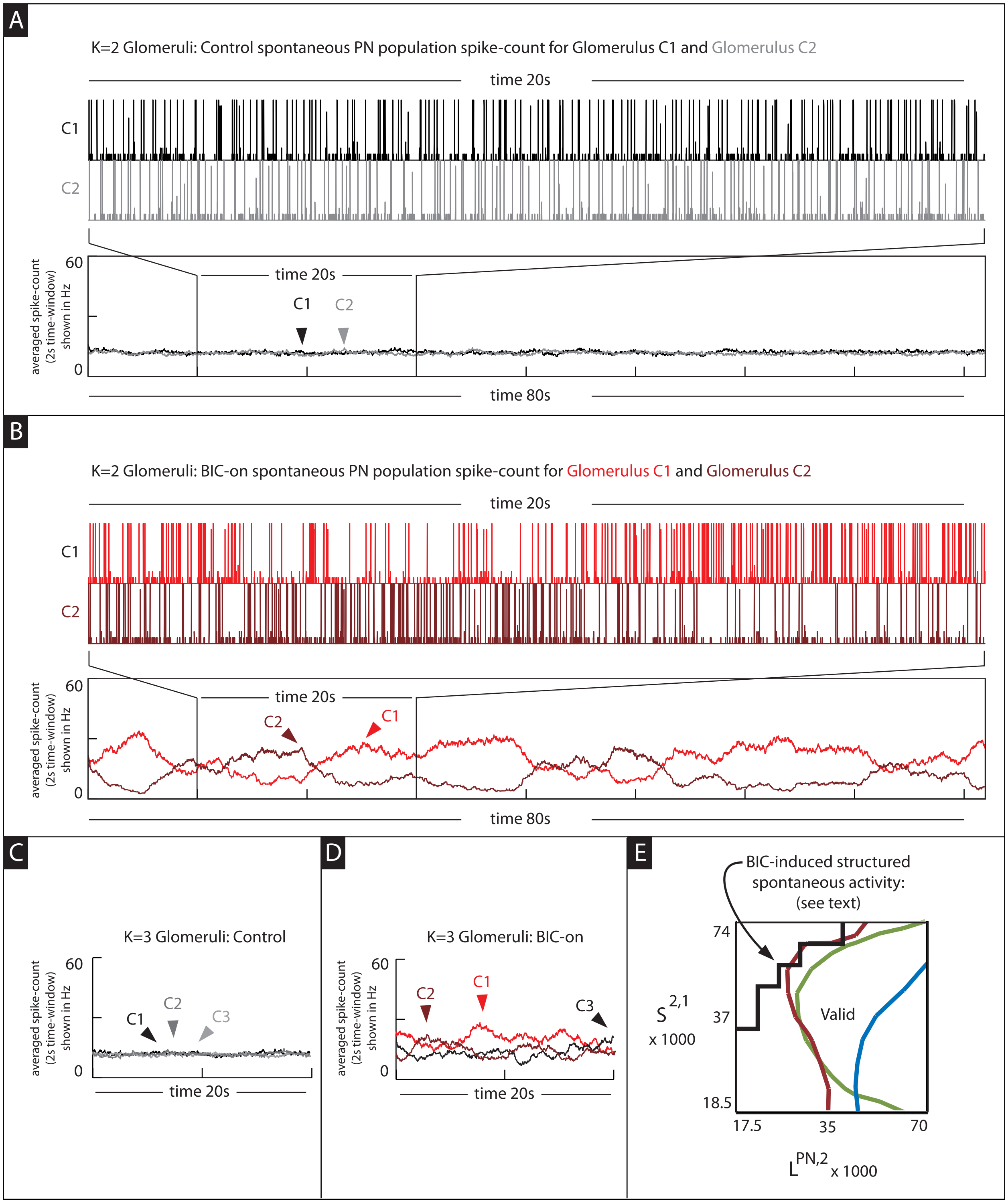

Figure 9: Here we summarize the 2-dimensional parameter-sweep shown in earlier figures; indicating the valid region both in terms of contours (left) and points in parameter-space that we actually simulated (right). Note that, for each simulated point, we have accumulated statistics across many randomly-generated networks.

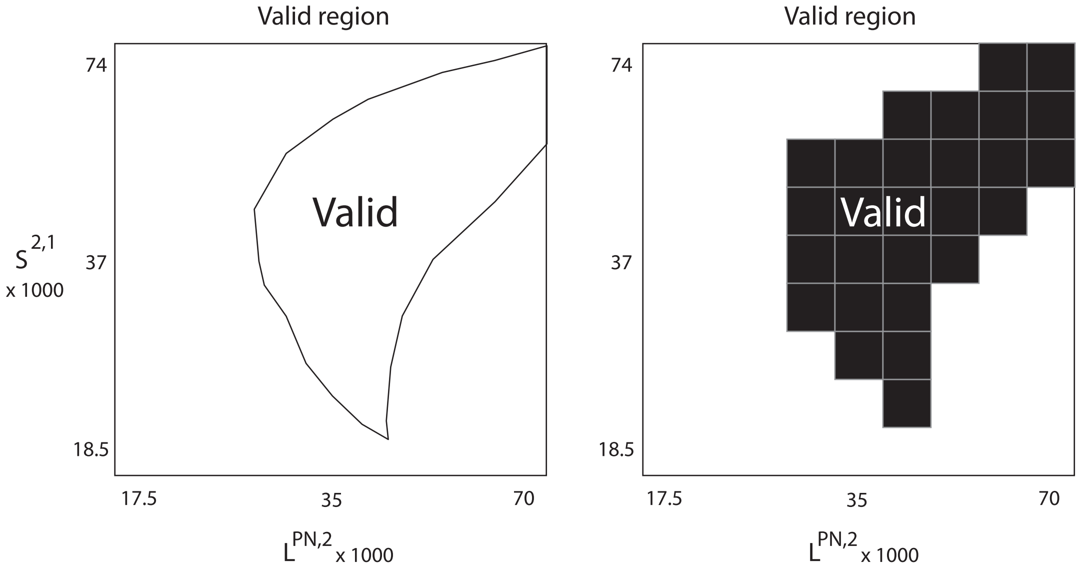

Supplement: Supplementary file 1 [file Presentation1.pdf]
